# Computer vision-based phenotyping for improvement of plant productivity: a machine learning perspective

**DOI:** 10.1093/gigascience/giy153

**Published:** 2018-12-06

**Authors:** Keiichi Mochida, Satoru Koda, Komaki Inoue, Takashi Hirayama, Shojiro Tanaka, Ryuei Nishii, Farid Melgani

**Affiliations:** 1 Bioproductivity Informatics Research Team, RIKEN Center for Sustainable Resource Science, 1-7-22 Suehiro-cho, Tsurumi-ku, Yokohama, Kanagawa 230-0045, Japan; 2 Microalgae Production Control Technology Laboratory, RIKEN Baton Zone Program, RIKEN Cluster for Science, Technology and Innovation Hub, 1-7-22 Suehiro-cho, Tsurumi-ku, Yokohama, Kanagawa 230-0045, Japan; 3 Institute of Plant Science and Resources, Okayama University, 2-20-1 Chuo, Kurashiki, Okayama 710-0046, Japan; 4 Kihara Institute for Biological Research, Yokohama City University, 641-12 Maioka-cho, Totsuka-ku, Yokohama, Kanagawa 244–0813, Japan; 5 Graduate School of Nanobioscience, Yokohama City University, 22-2 Seto, Kanazawa-ku, Yokohama, Kanagawa 236-0027, Japan; 6 Graduate School of Mathematics, Kyushu University, 744 Motooka, Nishi-ku, Fukuoka 819-0395, Japan; 7 Hiroshima University of Economics, 5-37-1, Gion, Asaminami, Hiroshima-shi Hiroshima 731-0138, Japan; 8 Institute of Mathematics for Industry, Kyushu University, 744 Motooka, Nishi-ku, Fukuoka 819-0395, Japan; 9 Department of Information Engineering and Computer Science, University of Trento, Via Sommarive 9, 38123 Trento, Italy

**Keywords:** machine learning, deep neural network, unmanned aerial vehicles, noninvasive plant phenotyping, hyperspectral camera

## Abstract

Employing computer vision to extract useful information from images and videos is becoming a key technique for identifying phenotypic changes in plants. Here, we review the emerging aspects of computer vision for automated plant phenotyping. Recent advances in image analysis empowered by machine learning-based techniques, including convolutional neural network-based modeling, have expanded their application to assist high-throughput plant phenotyping. Combinatorial use of multiple sensors to acquire various spectra has allowed us to noninvasively obtain a series of datasets, including those related to the development and physiological responses of plants throughout their life. Automated phenotyping platforms accelerate the elucidation of gene functions associated with traits in model plants under controlled conditions. Remote sensing techniques with image collection platforms, such as unmanned vehicles and tractors, are also emerging for large-scale field phenotyping for crop breeding and precision agriculture. Computer vision-based phenotyping will play significant roles in both the nowcasting and forecasting of plant traits through modeling of genotype/phenotype relationships.

## Background

Computer vision that extracts useful information from plant images and videos is rapidly becoming an essential technique in plant phenomics [1]. Phenomics approaches to plant science aim to identify the relationships between genetic diversities and phenotypic traits in plant species using noninvasive and high-throughput measurements of quantitative parameters that reflect traits and physiological states throughout a plant's life [2]. Recent advances in DNA sequencing technologies have enabled us to rapidly acquire a map of genomic variations at the population scale [3, 4]. Combining high-throughput analytical platforms for DNA sequencing and plant phenotyping has provided opportunities for exploring genetic factors for complex quantitative traits in plants, such as growth, environmental stress tolerance, disease resistance [5], and yield, by mapping genotypes to phenotypes using statistical genetics methods, including quantitative trait locus (QTL) analysis and genome-wide association studies (GWASs) [6]. Moreover, a model of the relationship between the genotype/phenotype map of individuals in a breeding population can be used to compute genome-estimated breeding values to select the best parents for new crosses in genomic selection in crop breeding [7, 8]. Thus, high-throughput phenotyping aided by computer vision with various sensors and algorithms for image analysis will play a crucial role for crop yield improvement in scenarios related to population demography and climate change [9].

**Figure 1:**
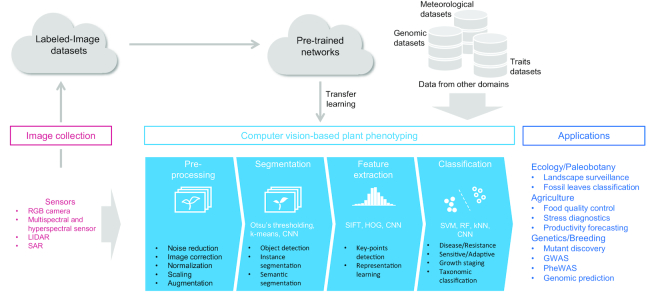
Schematic representation of a typical example scenario in computer vision-based plant phenotyping. Various sensors are used for collection of plant images. Large-scale collections of labeled image data are useful to design pretrained network models. A typical step of computer vision-based image analysis consists of the following steps: preprocessing, segmentation, feature extraction, and classification. Various ML-based algorithms, including convolutional neural network, are applied to the steps, such as segmentation, feature extraction, and classification. Pretrained networks are often adapted to reduce computational costs through fine-tuning. The classification step represents case-control phenotypes in plants; disease-resistance, sensitive-adaptive, morphological phenotypes; growth stages; and taxonomic classification. Exploration of associations among the classification results and genetic polymorphisms, agronomic traits, and meteorological observations will expand applications to areas such as ecology/paleobotany, agriculture, and genetics and breeding.

Machine learning (ML), an area of computer science, offers us data-driven prediction in various applications, including image analysis, which can aid typical steps of image analysis (i.e., preprocessing, segmentation, feature extraction, and classification) [10]. ML accelerates and automates image analysis, which improves throughput when handling labor-intensive sensor data. Algorithms based on deep learning, an emerging subfield of ML, often show more accurate performance compared with traditional approaches to computer vision-based tasks, including plant identification, such as PlantCLEF [11]. Moreover, ML-based algorithms often provide discriminative features associated with outputs extracted through their training process, which may enable us to dissect complex traits and determine visual signatures related to traits in plants. These outcomes of ML offer us opportunities for revitalizing methodologies in plant phenomics to improve throughput, accuracy, and resolution (Fig. 1).

In this review, we provide an overview of recent advances in computer vision-based plant phenotyping, which can contribute to our understanding of genotype/phenotype relationships in plants. Specifically, we summarize sensors and platforms recently developed for high-throughput plant image collection. Then, we also address recent challenges in computer vision-based plant image analysis and the typical image analysis process (e.g., segmentation, feature extraction, and classification), as well as its applications to large-scale phenotyping in genetic studies in plants, by highlighting ML-based approaches. Moreover, we showcase datasets and software tools that are useful to plant image analysis. Then, we discuss perspectives and opportunities for computer vision in plant phenomics.

## Review

### High-throughput image collection for large-scale plant phenotyping: sensors and platforms

#### Sensors for plant phenotyping

Various types of sensors can be encountered to acquire morphological and physiological information from plants [10] (Fig. 1). The basic sensors are digital cameras that are typically adopted for quick color and/or texture-based phenotyping operations. In a previous study [12], the authors presented a plant phenotyping system for stereoscopic red-green-blue (RGB) imaging to evaluate the growth rate of tree seedlings during post-seed germination through calculation of the increase in seeding height and the rate of greenness. Multispectral and hyperspectral sensors enable us to capture richer spectral information about plants of interest, thus allowing more in-depth phenotyping. Moreover, in another study [13], a methodology to monitor the responses of plants to stress by inspecting the hyperspectral features of diseased plants was established, describing a hyperspectral image "wordification" concept, in which images are treated as text documents by means of probabilistic topic models, which enabled automatic tracking of the growth of three foliar diseases in barley. An interesting analysis of vegetation-specific crop indices acquired by a multispectral camera mounted on an unmanned aerial vehicle (UAV) surveyed over a pilot trial of 30 plots was conducted in another prior analysis [14]. In this study, the authors exploited multiple indices to estimate canopy cover and leaf area index; they reported that the significant correlations among the normalized difference vegetation index, enhanced vegetation index, and normalized difference red edge index, which estimates leaf chlorophyll content, were useful for characterizing leaf area senescence features of contrasting genotypes to assess the senescence patterns of sorghum genotypes. Moreover, thermal infrared sensors offer additional complementary and useful information, particularly for determining the previsual and early response of the canopy to abiotic [15] and biotic stress [16] conditions. LiDAR (light detection and ranging)is another form of sensor characterized as a traditional remote sensing technique that is capable of yielding accurate three-dimensional (3D) data; this approach has been recently applied to plant phenotyping coupled with other sensors [17]. With these recent advancements, 3D reconstruction of plants enables us to identify phenotypic differences, including entire-plant and organ-level morphological changes, and combinatorial use of multiple sensors offers us opportunities to identify spectral markers associated with previsual signs of plant physiological responses.

#### Platforms

Plant phenotyping frameworks incorporate sensors with mobility systems, such as tray conveyors [18], aerial and ground vehicles [19], UAVs [20], and motorized gantries [21, 22], to continuously capture growth and physiology data from plants. An automated plant phenotyping system, called the phenome high-throughput investigator (PHI), allowed noninvasive tracking of plant growth under controlled conditions using an imaging station with various camera-based imaging units coupled with two growth rooms for growth of different types of plants (∼200 crop plants and ∼3,500 *Arabidopsis*, respectively) [23]. A computational pipeline for single leaf-based analysis with PHI was used to monitor leaf senescence and its progression in *Arabidopsis*. A high-throughput hyperspectral imaging system was designed for indoor phenotyping of rice plants [24] and was applied to quantifying agronomic traits based on hyperspectral signatures in a global rice collection of 529 accessions [24]. More recently, the RIKEN Integrated Plant Phenotyping System has been used owing to its accurate quantification of *Arabidopsis* growth responses and water use efficiency in the context of various water conditions [25]. PhenoTrac 4, a mobile platform for phenotyping under field conditions that is equipped with multiple passive and active sensors, was used to perform canopy-scale phenotyping of barley and wheat [26]. Another mobile platform, the Phenomobile system equipped with multiple sensors [27], has been investigated for its potential in field-phenotyping applications to examine agronomically important traits, such as stay-green [28]. These platforms for high-throughput plant phenotyping monitor plant growth noninvasively and continuously and evaluate phenotypic differences quantitatively throughout the life cycle at the population scale; this facilitates the identification of genetic factors associated with traits related to growth and development.

### Computer vision-based plant phenotyping

In this section, we discuss recent advances in image analysis methodologies for plant phenotyping; these methodologies consist of four major steps, i.e., preprocessing, segmentation, feature extraction, and classification. In each of the following subsections, we highlight ML-based approaches discussed in recently published literature.

#### Preprocessing

Preprocessing is a preliminary step of image analysis that aims to organize data properties to facilitate subsequent steps and even derive reasonable final outcomes. Particularly when we target images acquired under field conditions, unlike in controlled environments, image preprocessing contributes to enhancement of image processing quality. A simple preprocessing step is image cropping, which extracts rectangles containing target objects out of an image. Data transformation techniques, such as gray scale conversion, normalization, standardization, and contrast enhancement, are also adopted during preprocessing. Data augmentation is another example of preprocessing whose underlying goal is to increase variations in images in datasets, resulting in making pattern analysis more robust and generalized. Various techniques, such as image scaling, rotation, flipping, and noise addition, are often used for data augmentation.

#### Segmentation

Segmentation represents a first important step to extract information of targets from preprocessed image data by separating a set of pixels including objects of interest in images (Fig. 1), enabling the identification and quantification of areas corresponding to particular organs in plants automatically. To develop a pipeline to automatically count maize tassels, a deep convolutional neural network (CNN) model, resulting from learning of the Maize Tassels Counting dataset [29], was applied, and plausible results were obtained with an absolute error of 6.6 and a mean squared error of 9.6 [29]. To automatically count tomato fruits, a deep CNN based on the Inception-ResNet was applied through training on synthetic data and tested on real data; 91% counting accuracy was obtained [30]. In addition to these model-driven approaches, various image-driven approaches have been applied for autosegmentation of plant organs. For example, in a previous study [31] in which images were acquired by X-ray micro-computed tomography, a method for accurate extraction and measurement of spike and grain morphometric parameters of wheat plants was established based on combinatorial use of adaptive threshold and morphology algorithm and applied to examine spike and grain growth of wheat exposed to high temperatures under two different water treatments. Another study [32] proposed a method for resegmentation and assimilated details that were missed in the *a priori* segmentation, which was useful to improve the accuracy of determination of sharp features, such as leaf tips, twists, and axils of plants. Moreover, hybrid approaches integrating model-based and image-based approaches have been applied for segmentation of plant shape and organs. For example, in a previous study [33], a decision tree-based ML method with multiple color space and a method combining mean shift and threshold based on the hue, saturation, and value color space, were applied for segmentation of top- and side-view images in maize, yielding an accuracy of 86% in estimation of ear position in 60 maize hybrids. In wheat, researchers used an improved color index method for plant segmentation, followed by a neural network-based method with Laws texture energy; this method enabled them to detect spikes with an accuracy of more than 80% [34]. Rzanny et al. [35] reported systematic guidelines for workloads of image acquisition (perspective, illumination, and background) and preprocessing (nonprocessed, cropped, and segmented) and assessed the impact of segmentation and other preprocessing techniques on recognition performances. These recent attempts to improve the accuracy of segmentation enabled us to automatically identify and quantify plant organs and evaluate the biomass and yields of fruits and grains. We were also able to improve reproducibility in phenotyping by replacing conventional human-based phenotyping, which is often time consuming and labor intensive.

#### Feature extraction

Feature extraction is a step to create a set of significant and nonredundant information that can sufficiently represent images. Because pattern recognition performance in computer vision heavily depends on the quality of the extracted features, a number of approaches have been attempted in various areas, including plant phenotyping.

Typically, features are hand-chosen based on characteristics of objects in images, such as pixel intensities, gradient, texture, and shape. For example, in a previous study [36, 37], the authors extracted features such as shape, color, and texture (contrast, correlation, homogeny, entropy) from wheat grains to classify their accessions. Moreover, in another study [38, 39], the authors used an elliptic Fourier descriptor and the texture feature set called Haralick's texture descriptors to characterize seeds of plants for taxonomic classification. With a representative feature extraction tool, Scale Invariant Features Transforms (SIFT), which acts as an invariant feature descriptor not only to scale but also rotation, illumination, and viewpoint, Wilf et al. [40] generated codebooks for dictionary learning, and their results demonstrated the effectiveness of their approach on taxonomic classification through leaves. The bag-of-keypoints/bag-of-visual-words method, an analogy to the bag-of-words method for text categorization using keywords [41–44], has also been used as a feature representation tool in image analysis in which the SIFT algorithm is used for keypoint detection and local feature description [45, 46]. The bag-of-keypoints method and the SIFT algorithm were applied to RGB color images of wheat under field conditions for growth stage identification [47].

Recently, CNN-based approaches have shown remarkable advancement, and their applications have been expanded to myriad areas, including computer vision [48–50], which can automatically extract features from images and classify them. Therefore, unlike hand-chosen feature-based algorithms, CNNs create and train classifiers without explicit feature extraction steps. Moreover, pretrained CNNs can be used as a simple feature extractor [51]. Based on these advantages of CNNs, many CNN-based strategies have been developed and are now widely used for pattern-recognition and image-classification tasks, even for plant phenotyping. Notably, the authors of previous studies [52, 53] illustrated feature extraction processes based on CNNs, which learn hierarchical features through network training for taxonomic classification tasks on leaf image datasets. Recent outcomes of CNN-based classification in plant phenotyping are discussed in the following sections.

#### Classification

In classification steps, outcomes from the previous three steps are obtained. Here, we address classification techniques, including ML-based techniques, recently applied in plant phenotyping, highlighting two major applications: taxonomic classification and classification of plant physiological states.

##### Taxonomic classification

Computer vision-based taxonomic classification plays an essential role in plant phenotyping to automatically distinguish target species for phenotyping from other plants, which is particularly important for images from real fields. Wäldchen and Mäder have thoroughly summarized the literature on computer vision-based species identification published by 2016 [54]. In recent years, because techniques for computer vision-based species identification have shown dramatically improved accuracy and expanded applications for various plant groups through handcrafted feature-based and CNN-based approaches, we highlight studies describing plant taxonomic classification by means of these two distinctive approaches (Table 1).

**Table 1:** Examples of taxonomic classification approaches

Approach	Object	Features/feature extractor	Classifier	Reference
Custom feature-based approach	Seed	Elliptic Fourier descriptor, Haralick's texture descriptor, morpho-colorimetric feature	LDA	[38, 39]
	Grain	Shape, color, texture features	MLP	[36]
			ANFIS	[37]
		SIFT, sparse coding	SVM	[40]
		Fourier descriptor, leaf shapes, vein structure		[55]
	Leaf	Pretrained CNN		[35]
		Ffirst		[56]
		Texture features	LWSRC	[57]
	Bark	Ffirst	SVM	[56]
	Tree	Reflectance, minimum noise fraction transformation, narrowband vegetation indices, airborne imaging spectroscopy features	SVM, RF	[58]
CNN-based approach	Grain			[59]
	Ear, spike, spikelet			[60]
	Leaf	CNN		[52, 53, 61, 62]
	Root			[62]
	Various organs			[63, 61, 56]

Ffirst: Fast Features Invariant to Rotation and Scale of Texture.

In a custom feature-based approach, Wilf et al. [40] attempted to classify leaf images into labels of major groups (such as families and orders) in the taxonomic category. They used SIFT and a sparse coding approach to extract the discriminative features of leaf shapes and venation patterns, followed by a multiclass support vector machine (SVM) classifier for grouping. A sparse representation was also used by Zhang et al. [57] as a part of their processes for classifying plant species from RGB color leaf images; they demonstrated the superiority of their approach in identification on leaf image datasets. As a case study, a Turkish research group investigated the capability of computer vision algorithms to classify wheat grains into bread wheat and durum wheat based on grain images captured by high-resolution cameras [36, 37]. They used two types of neural networks: a multilayer perceptron (MLP) with a single hidden layer and an adaptive neuro-fuzzy inference system (ANFIS). They selected seven discriminative grain features, incorporating aspects of shape, color, and texture, and achieved greater than 99% accuracy on the grain classification task. Another group examined two taxonomic classification tasks: the *Malva* alliance taxa and genus *Cistus* taxa [38, 39]. They acquired digital images of seeds using a flatbed scanner; extracted morphometric, colorimetric, and textural seed features; and then performed taxonomic classification with stepwise linear discriminant analysis (LDA). Species identification from herbarium specimens with computer vision approaches was first presented in 2016, in which Unger et al. classified German trees into tens of classes with images of herbarium specimens photographed at a high resolution [55]. Their analytical processes were composed of preprocessing, normalization, and feature extraction with Fourier descriptors, leaf shape parameters, and vein texture, followed by SVM classification. In this study, they demonstrated the potential of computer visions for taxonomic identification, even when using discolored leaf images of herbarium specimens. Using rather different data for species classification, Piiroinen et al. [58] attempted tree species identifications with airborne laser scanning and hyperspectral imaging in a diverse agroforestry area in Africa, where a few exotic tree species are dominant and most native species occur less frequently. Despite this challenge, they demonstrated that ML-based analytical approaches using SVMs and random forests (RFs) could achieve reasonable tree species identification based on airborne-sensor images.

In the last few years, many CNN-based approaches have been developed for the taxonomic classification of plants [52, 53]. Using a dataset of accurately annotated images of wheat lines, the authors in a previous study [60] applied a CNN-based model to perform feature location regression to identify spikes and spikelets and carried out image-level classification of wheat awns, suggesting the feasibility of employing CNN-based models in multiple tasks by coordinating their network architecture. In this study, the authors also suggested that the images of wheat in the training dataset, which were acquired using a consumer-grade 12 MP camera, could be favorable for training the CNN-based model. A comparative assessment between CNN-based and custom feature-based approaches was performed in a rice kernel classification task [59]. In this assessment, the authors compared a deep CNN with *k*-nearest neighbor (kNN) algorithms and SVMs, along with custom features, such as a pyramid histogram of oriented gradients and GIST, and showed that the CNN surpassed the kNN and SVM algorithms in classification accuracy.

Although CNNs usually require large amounts of data and extensive computational load and time, transfer learning (i.e., the reuse and fine-tuning of pretrained networks for other tasks) is a promising technique for mitigating these costs [56, 63, 61]. Ghazi et al. [63] fine-tuned the three deep neural networks that performed well in the ImageNet Large-Scale Visual Recognition Challenge, i.e., AlexNet [64], GoogLeNet [65], and VGGNet [66], for a large classification dataset of 1,000 species from PlantCLEF2015, aiming to construct a neural network model for taxonomic classification. In this study, the authors compared approaches based on fine-tuning and training from scratch and demonstrated that the fine-tuning approach had a slight edge in species identification. Carranza-Rojas et al. [61] applied a pretrained CNN to herbarium species classification. Sulc and Matas [56] utilized a pretrained 152-layer residual network model [67] and the Inception-ResNet-v2 model [68] for plant recognition in nature, in which views of plants or their organs differ significantly and in which the background is often cluttered. Moreover, the authors proposed the use of a textual feature, called Fast Features Invariant to Rotation and Scale of Texture (Ffirst), to computationally recognize bark and leaves from segmented images. They demonstrated improved recognition rates with this feature for a small computational cost. Pound et al. [62] applied CNNs to two types of identification tasks, classification and localization, with megapixel images taken by multiple cameras. In this classification task, the authors succeeded in identifying root tips and leaf-ear tips with accuracies of 98.4% and 97.3%, respectively, with deep CNNs and extended trained classifiers for localizing plant root and shoot features. Rzanny et al. [35] summarized workloads of image acquisition and the impact of preprocessing on accuracy in image classification and concluded that images taken from the top sides of leaves were most effective for processing of nondestructive leaf images. Interestingly, in this study, the authors recorded leaf images using a smartphone (an iPhone 6) in diverse situations, including natural background conditions, followed by feature extraction with the pretrained ResNet-50 CNN and classification with an SVM.

##### Classification of plant physiological states

The applications of computer vision-based image classification have been expanding to include description of developmental stages, physiological states, and qualities of plants (Table 2). Autonomous phenotyping systems equipped with multiple sensors for data acquisition have enabled us to collect information associated with internal and surface changes in plants [69–71]. Through exploration of the relationships between multidimensional spectral signatures and the physiological properties of plants, we may be able to identify novel spectral markers that can reflect various plant physiological states [69, 72–74]. Moreover, noninvasive data acquisition enables us to continuously monitor phenotypic changes over time in plant life courses [75]. Therefore, computer vision-based plant phenotyping provides opportunities for early identification and detection of fine changes in plant growth, assisting crop diagnostics in precision agriculture.

**Table 2:** Examples of approaches for classification of physiological states

Approach	Object	Features/feature extractor	Classifier	Reference
Custom feature-based approach	Ear (growth stages)	SIFT + bag of keypoints	SVM	[47]
	Grain (quality assessment)	Weibull distribution model parameter features	SVM	[76]
	Leaf	Spectral vegetation indices	Spectral Angle Mapper	[77]
CNN-based approach	Leaf	CNN		[78, 79]

ML-based and statistical algorithms have been used to extract structural features from plant images for tasks such as tissue segmentation, growth stage classification, and quality evaluation in plants [80]. Multiple ML-based algorithms, such as kNN, naive Bayes classifier, and SVM algorithms, have been examined in segmentation processes for detecting aerial parts of plants, and the findings suggested that different algorithms would be preferable for segmenting images of the visible and near infrared spectra [81]. The bag-of-keypoints method was recently applied to RGB color images of wheat under field conditions and demonstrated its ability to identify growth stages from heading to flowering [47]. Quality inspection of harvested crop grains can also be assisted by computer vision-based approaches to describe the relationships between the visual appearance and qualities of grains. A method based on omnidirectional Gaussian derivative filtering was proposed to extract visual features from images of granulated products (e.g., cereal grains) and applied to automated rice quality classification [76].

Computer vision-based image classification techniques have also been widely used to identify symptoms of disease in plants. Hyperspectral imaging was applied to detect and quantify downy mildew symptoms caused by *Plasmopara viticola* in grapevine plants [77]. Recent deep learning-based techniques have led to improvements in throughput and accuracy for detecting disease symptoms in plants. Mohanty et al. [78] demonstrated the feasibility of using a deep CNN to detect 26 diseases in 14 crop species by fine-tuning popular pretrained deep CNN architectures, such as AlexNet [64] and GoogLeNet [65], with a publicly available 54,306-image dataset of diseased and healthy plants from PlantVillage. Transfer learning was also used to train CNN models for detecting of disease symptoms in crops, such as olives [79].

#### Deep neural network-based image analysis with end-to-end learning

Beyond applications in each of the typical steps in computer vision-based image analysis, deep CNNs have automated approaches to directly identify biological instances from image data through end-to-end training. Faster region-based CNN (R-CNN) is a CNN-based region proposal network that enables representation of high-quality region proposals through end-to-end training [82]. Jin et al. demonstrated the performance of a faster R-CNN-based model for segmentation of maize plants from terrestrial LiDAR data [83]. In addition to faster R-CNN, Fuentes et al. examined two other CNN-based end-to-end frameworks for object detection: region-based fully convolutional network (FCN) and single shot multibox detector to detect diseases and pests in tomatoes [84]. Shelhamer et al. proposed an FCN that enables end-to-end training for sematic image segmentation by pixel-wise object labeling [85], which has been applied to generate weed distribution maps from UAV images [86, 87]. Moreover, FCN has also been applied to segment a particular region of an image into each instance (pixel-wise instance segmentation) for computer vision-based image and scene understanding, which should facilitate various instance segmentation tasks in plant phenotyping, such as the Leaf Segmentation and Counting Challenges [88].

### Application of computer vision-assisted plant phenotyping for gene discovery

Modern techniques in computer vision can aid digital quantification of various morphological and physiological parameters in plants and are expected to improve the throughput and accuracy of plant phenotyping for population-scale analyses [89, 90]. Combined with recent advances in high-throughput DNA sequencing, the automated acquisition of plant phenotypic data followed by computer vision-based extraction of phenotypic features provides opportunities for genome-scale exploration of useful genes and modeling of the molecular networks underlying complex traits related to plant productivity, such as growth, stress tolerance, disease resistance, and yield [9, 75, 91–93].

#### Autoscreening of mutants

Large-scale mutant resources have played crucial roles in reverse genetics approaches in plants, and computer vision-assisted phenotype analyses can provide new insights into gene functions and molecular networks related to traits in plants. A computer vision-based tracking approach to organ development revealed temperature-compensated cell production rates and elongation zone lengths in roots through comparative image analysis of wild-type *Arabidopsis* and a phytochrome-interacting factor 4- and 5*-*double mutant of *Arabidopsis* [94]. A new clustering technique, nonparametric modeling, was applied to a high-throughput photosynthetic phenotype dataset and showed efficiency for discriminating *Arabidopsis* chloroplast mutant lines [95]. In rice, a large-scale T-DNA insertional mutant resource was developed and applied to phenotyping 68 traits belonging to 11 categories and 3 quantitative traits, screened by well-trained breeders under field conditions [96]. These findings led us to question whether using computer vision-based phenotyping to digitize growth patterns may bridge physiological features detected by machines and agronomically important traits observed by breeders.

#### Phenotyping for genetic mapping and prediction of agronomic traits

Phenotyping a set of accessions provides a dataset beneficial for exploring novel interactions between genetic factors that influence productivity [97]. In several instances, automated plant phenotyping systems have been applied for characterizing the growth patterns of diverse crop accessions grown under controlled conditions. An automated plant phenotyping system, the rice automatic plant phenotyping platform, also assisted in quantifying 106 traits in a maize population composed of 167 recombinant inbred lines across 16 developmental stages and identified 998 QTLs for all investigated traits [98]. In another study using a high-throughput phenotyping system, PhenoArch [99] represented differences in daily growth among 254 maize hybrids in different soil and water conditions and revealed genetic loci affecting stomatal conductance through a genome-wide association study using the phenomic dataset [100]. A study using multiple sensors, such as hyperspectral, fluorescence, and thermal infrared sensors, demonstrated a time course heritability of traits found in a set of 32 maize inbred lines in greenhouse conditions [101]. These examples indicate that noninvasive phenotyping, unlike destructive measurement, enables us to characterize growth trajectories to identify phenotypic differences in development and phenological responses over time that may influence eventual traits, such as biomass and yield [102].

For phenotyping crops under field conditions, the combined use of multiple sensors and techniques for image analysis has proven to be efficient for comprehensively identifying genetic and environmental factors related to phenotypic traits. With a dataset of 14 photosynthetic parameters and 4 morphological traits in a diverse rice population grown in different environments, a stepwise feature-selection approach based on linear regression models assisted in identifying physiological parameters related to the variance of biomass accumulation in rice [103]. In a study of poplar trees, UAV-based thermal imaging of a full-sib F_2_ population across water conditions showed the potential of UAV-based imaging for field phenotyping in tree genetic improvements [104]. In a genetic study of iron deficiency chlorosis using an association panel of soybeans, supervised machine learning-based image classification allowed identification of genetic loci harboring a gene involved in iron acquisition, suggesting that computer vision-based plant phenotyping provides a promising framework for genomic prediction in crops [105]. In sorghum, UAV-based remote sensing was used to measure plant height for genomic prediction modeling, demonstrating that UAV-based phenotyping with multiple sensors is efficient for generating datasets for genomic prediction modeling [106].

### Datasets and software tools for plant phenotyping

#### Datasets

Public datasets from various platforms for plant phenotyping will provide data for developing analytical methods in computer vision-based plant phenotyping. In a recent Kaggle competition, an image dataset of approximately 960 unique plants belonging to 12 species was used to create a classifier for plant taxonomic classification from a photograph of a plant seedling [107]. In a previous study [108], the authors introduced the first dataset for computer vision-based plant phenotyping, which was made available in a separate report [109].

A comprehensive phenotype dataset is available in *Arabidopsis* and will be useful as a reference image-set for the growth and development of model plant species when assessing methods in computer vision-based plant phenotyping [110]. In maize, the datasets used in two previous studies [33, 111] are available in other reports [112]. Moreover, the PlantCV website has provided image datasets acquired in grass species, such as rice, *Setaria*, and sorghum [113]. Additionally, the importance of integrating traits, phenotypes, and gene functions based on ontologies has increased dramatically; plant ontology, plant trait ontology, plant experimental conditions ontology, and gene ontology can facilitate semantic integration of data and corpuses rapidly generated from plant genomics and phenomics [114].

#### Software tools

**Figure 2:**
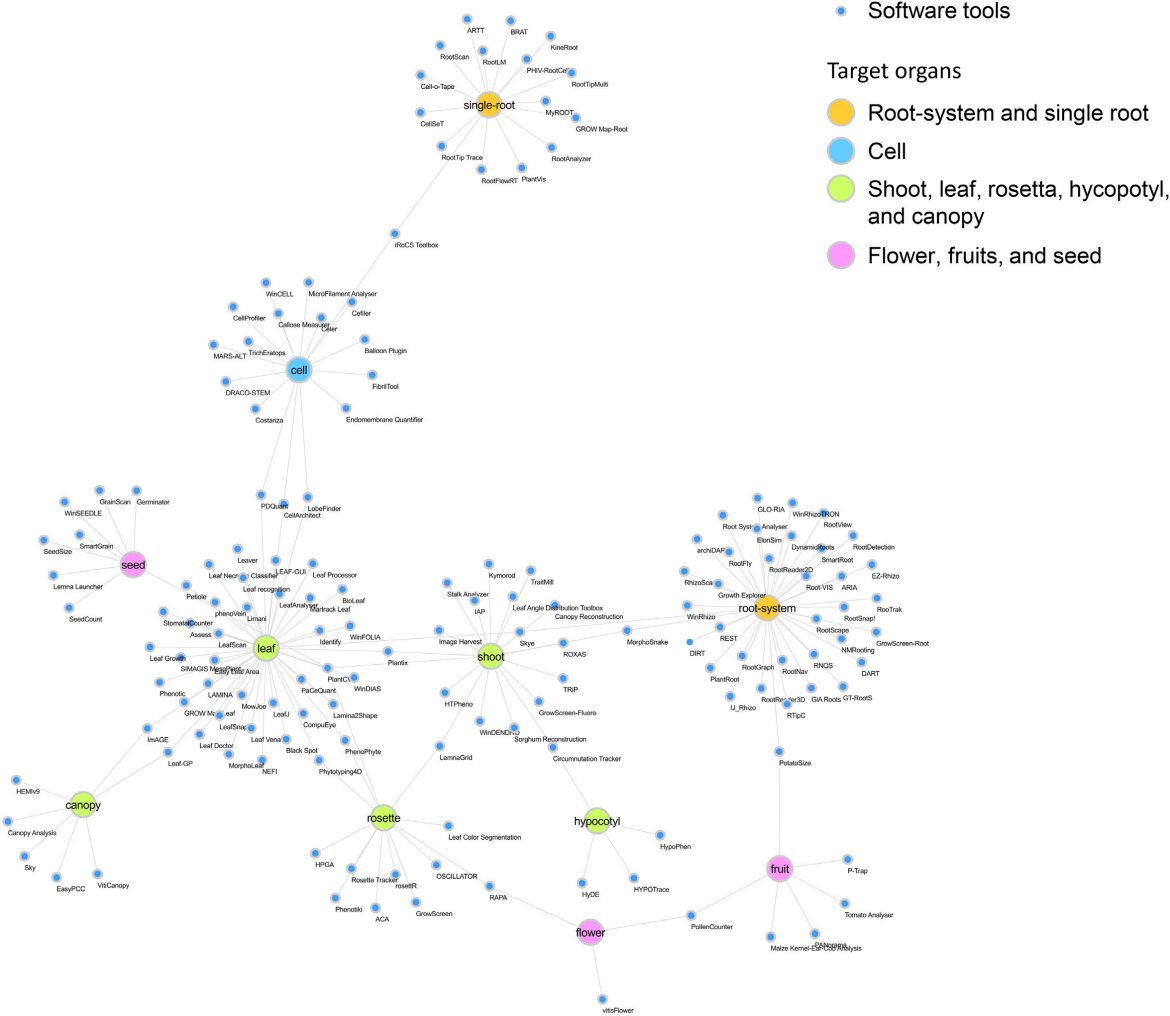
An ecosystem map of software tools for plant image analysis. The network-formed map consists of 169 software tools whose targets are particular plant organs based on the plant image analysis database [117]. The nodes represent the software tools and their target plant organs represented using Cytoscape 3.0 [123].

Various types of software tools have been established to aid steps of image analysis in plant phenotyping. The Plant Image Analysis website [115] showcases 172 software tools and 28 datasets (as of 9 August 2018) for analysis of plant image datasets, aiming to provide a user-friendly interface to find solutions and promote communication between users and developers [116, 117]. Figure 2 shows the ecosystem of software tools for plant phenotyping based on the plant image analysis database, in which software tools are connected to plant organs of an analytical target, indicating that the ecosystem is growing, particularly for images from leaves, shoots, and roots. Table 3 shows examples of software tools recently developed for plant phenotyping by image processing, which take advantage of ML-based algorithms. Leaf Necrosis Classifier supports detection of leaf areas that show necrotic symptoms with combinatorial use of MLP and self-organizing maps [118]. EasyPCC evaluates the ground coverage ratio accurately through image data acquired under field conditions and uses a pixel-based segmentation method that applies a decision-tree-based segmentation model [119]. Leaf-GP is a software tool that is used for quantification of various growth phenotypes from large image series, applying Python-based machine learning libraries, which were used to analyze the growth of *Arabidopsis* and wheat [120]. A deep CNN-based approach was applied to develop StomataCounter for detection of stomatal pores in microscopic images [121]. Moreover, the mobile app Plantix enables diagnosis and customized options for detection of plant diseases, pests, and nutrient deficiencies to users who send a picture of a plant [122], in which it synergistically uses a deep learning, crowd-sourced database to identify plant diseases on various crops worldwide.

**Table 3:** Software tools recently developed for plant image analysis that use machine learning-based algorithms

Name	Algorithms	Functionalities	Reference, URL
Leaf Necrosis Classifier	Multilayer perceptron and self-organizing maps	Detection of leaf areas showing necrotic symptoms	[118]
EasyPCC	Decision-tree-based segmentation model	Quantification of ground coverage ratio from image data acquired under field conditions	[119, 124]
Leaf-GP	Python-based machine learning libraries	Quantification of multiple growth phenotypes from large image series	[120, 125]
StomataCounter	Deep CNN	Counting stomate pores	[121]
Plantix	Deep learning	Diagnosing plant diseases, pest damage, and nutrient deficiencies	[122]

## Conclusions and Perspectives

In recent years, computer vision-based plant phenotyping has rapidly grown as a multidisciplinary area that integrates knowledge from plant science, ML, spectral sensing, and mechanical engineering. With large-scale plant image datasets and successful CNN-based algorithms, the tools available for computer vision-based plant phenotyping have shown remarkable advancements in plant recognition and taxonomic classification. Repositories for pretrained models for plant identification play significant roles in rapidly implementing models for new phenotyping frameworks through fine-tuning. Moreover, these models aid in the further improvement of recognition accuracy in more challenging tasks, such as multilabel segmentation of multiple organs and species under natural environments. These efforts to improve accuracy, throughput, and computational costs for automated plant identification will provide an analytical basis for computer vision-based plant phenotyping beyond the capacity of human vision-based observation.

Computer vision-based plant phenotyping has already played important roles in monitoring the physiological states of plants for agricultural applications, such as disease symptoms and grain quality. Meta-analysis of the spectral signatures of crops associated with growth stage, physiological states, and environmental conditions will provide useful clues for preventive interventions in farming. Moreover, spectral signatures observed during earlier growth stages of crops, which are associated with eventual agronomic traits such as yield and quality, will be beneficial phenotypes for dissecting the interactions between genetic and environmental factors and for increasing genetic gain in crop breeding.

Assorted sensors have assisted plant phenotyping under both controlled and field conditions and will aid our discovery of genes involved in agronomic traits and our understanding of their functions through statistical explorations of genome-phenome relationships, such as GWASs and phenome-wide association studies [126, 127] in plants. High-throughput automated phenotyping will allow common garden experiments to be performed with diverse genetic resources in order to elucidate the genetic bases of adaptive traits in plants [128]. Noninvasive and population-scale plant phenotyping will provide us opportunities to investigate interactions between internal and external factors related to plant growth and development, dissecting the effects of earlier life-course exposures onto later agronomic outcomes. Moreover, with the recent success of ML-based approaches in predicting individual traits in genomic prediction [129] and cohort studies [130, 131], computer vision-based phenotyping will play significant roles in both nowcasting and forecasting of plant traits through modeling genotype/phenotype relationships.

## 

### Abbreviations

3D: three-dimensional; ANFIS: adaptive neuro-fuzzy inference system; CNN: convolutional neural network; FCN: fully convolutional network; Ffirst: Fast Features Invariant to Rotation and Scale of Texture; GWAS: genome-wide association study ; kNN: k-nearest neighbor; LDA: linear discriminant analysis; ML: machine learning; MLP: multilayer perceptron; PHI: phenome high-throughput investigator; QTL: quantitative trait locus; R-CNN: region-based convolutional neural network; RF: random forest; RGB: red-green-blue; SIFT: Scale Invariant Features Transforms; SVM: support vector machine; UAV: unmanned aerial vehicle.

### Competing interests

The authors declare that they have no competing interests.

### Funding

The work was supported by Core Research for Evolutionary Sciecne and Technology (CREST) of the Japan Science and Technology Agency (JST).

### Author contributions

Conceptualization: K.M. and F.M. Supervision: T.H. and R.N. Funding acquisition: K.M. and T.H. Writing—original draft preparation: K.M., S.K., K.I., T.H., S.T., R.N., and F.M. Writing—review and editing: K.M., S.K., K.I., and R.N. Visualization: K.M., S.K., and K.I.

## Supplementary Material

GIGA-D-18-00215_Original_Submission.pdf

GIGA-D-18-00215_Revision_1.pdf

GIGA-D-18-00215_Revision_2.pdf

Response_to_Reviewer_Comments_Original_Submission.pdf

Response_to_Reviewer_Comments_Revision_1.pdf

Reviewer_1_Report_1_(Original_Submission) -- Andrew French 7/3/2018 Reviewed

Reviewer_1_Report_1_Revision_1 -- Andrew French 9/25/2018 Reviewed
