# Supplementary material for: Computer vision-based phenotyping for improvement of plant productivity: a machine learning perspective
Source: Gigascience. 2018 Dec 6;8(1):giy153. doi: 10.1093/gigascience/giy153 (PMC6312910; doi:10.1093/gigascience/giy153)

GigaScience

Plant phenotyping accelerated by computer vision with machine leaning

--Manuscript Draft--

**Manuscript Number:**	GIGA-D-18-00215
**Full Title:**	Plant phenotyping accelerated by computer vision with machine leaning
**Article Type:**	Review
**Funding Information:**	Core Research for Evolutional Science and Technology — Dr. Keiichi Mochida; Dr Takashi Hirayama
**Abstract:**	Employing computer vision to extract useful information from images and videos is becoming a key technique in identifying phenotypic changes in plants. Recent advances in image analysis empowered by machine learning-based techniques, including convolutional neural network-based modeling, have expanded their application to object segmentation and classification tasks, which assist high-throughput plant phenotyping. Combinatorial use of multiple sensors to acquire various spectra allow us to noninvasively obtain series of datasets along with developments and physiological responses throughout a plant's life. Automated phenotyping platforms accelerate the elucidation of gene functions that associate with traits in model plants under controlled conditions. Remote sensing techniques with unmanned vehicles and tractors are also emerging for large-scale field phenotyping for crop breeding, as well as precision agriculture. From these perspectives, we review the emerging aspects of computer vision that are rapidly blooming for automated plant phenotyping.
**Corresponding Author:**	Keiichi Mochida<br><br>JAPAN
**Corresponding Author Secondary Information:**	
**Corresponding Author's Institution:**	
**Corresponding Author's Secondary Institution:**	
**First Author:**	Keiichi Mochida
**First Author Secondary Information:**	
**Order of Authors:**	Keiichi Mochida
	Satoru Koda
	Komaki Inoue
	Takashi Hirayama
	Shojiro Tanaka
	Ryuei Nishii
	Farid Melgani
**Order of Authors Secondary Information:**	
**Additional Information:**	
**Question**	**Response**
Are you submitting this manuscript to a special series or article collection?	No
**Experimental design and statistics**	No

Powered by Editorial Manager® and ProduXion Manager® from Aries Systems Corporation

Full details of the experimental design and statistical methods used should be given in the Methods section, as detailed in our Minimum Standards Reporting Checklist. Information essential to interpreting the data presented should be made available in the figure legends.<br><br>Have you included all the information requested in your manuscript?	
If not, please give reasons for any omissions below.<br><br>as follow-up to "**Experimental design and statistics**<br><br>Full details of the experimental design and statistical methods used should be given in the Methods section, as detailed in our Minimum Standards Reporting Checklist. Information essential to interpreting the data presented should be made available in the figure legends.<br><br>Have you included all the information requested in your manuscript?<br><br>"	There are no experiments and statistical analyses used in this review.
**Resources**<br><br>A description of all resources used, including antibodies, cell lines, animals and software tools, with enough information to allow them to be uniquely identified, should be included in the Methods section. Authors are strongly encouraged to cite Research Resource Identifiers (RRIDs) for antibodies, model organisms and tools, where possible.<br><br>Have you included the information requested as detailed in our Minimum Standards Reporting Checklist?	Yes
**Availability of data and materials**<br><br>All datasets and code on which the conclusions of the paper rely must be either included in your submission or deposited in publicly available repositories (where available and ethically appropriate), referencing such data using	No

a unique identifier in the references and in the “Availability of Data and Materials” section of your manuscript.<br><br>Have you have met the above requirement as detailed in our Minimum Standards Reporting Checklist?	
If not, please give reasons for any omissions below.<br><br>as follow-up to "**Availability of data and materials**<br><br>All datasets and code on which the conclusions of the paper rely must be either included in your submission or deposited in publicly available repositories (where available and ethically appropriate), referencing such data using a unique identifier in the references and in the “Availability of Data and Materials” section of your manuscript.<br><br>Have you have met the above requirement as detailed in our Minimum Standards Reporting Checklist?<br><br>"	There are no datasets and code on which the conclusions of this review.

Manuscript

Click here to download Manuscript Review_with_Dr._Farid.for_submit.docx

Plant phenotyping accelerated by computer vision with machine leaning

Keiichi Mochida[1,2,3,4,5*], Satoru Koda[6], Komaki Inoue[1], Takashi Hirayama[3], Shojiro Tanaka[7], Ryuei Nishii[8] and Farid Melgani[9]

[1]Bioproductivity Informatics Research Team, RIKEN Center for Sustainable Resource Science, 1-7-22 Suehiro-cho, Tsurumi-ku, Yokohama, Kanagawa 230-0045, Japan.

[2]Microalgae Production Control Technology Laboratory, RIKEN Baton Zone Program, RIKEN Cluster for Science, Technology and Innovation Hub, 1-7-22 Suehiro-cho, Tsurumi-ku, Yokohama, Kanagawa 230-0045, Japan.

[3]Institute of Plant Science and Resources, Okayama University, 2-20-1 Chuo, Kurashiki, Okayama 710-0046, Japan.

[4]Kihara Institute for Biological Research, Yokohama City University, 641-12 Maioka-cho, Totsuka-ku, Yokohama, Kanagawa 244-0813, Japan.

[5]Graduate School of Nanobioscience, Yokohama City University, 22-2 Seto, Kanazawa-ku, Yokohama, Kanagawa 236-0027, Japan.

[6]Graduate School of Mathematics, Kyushu University, 744 Motooka, Nishi-ku, Fukuoka 819-0395, Japan.

[7]Hiroshima University of Economics, 5-37-1, Gion, Asaminami, Hiroshima-shi Hiroshima, 731-0138 Japan.

[8]Institute of Mathematics for Industry, Kyushu University, 744 Motooka, Nishi-ku, Fukuoka 819-0395, Japan.

[9]Department of Information Engineering and Computer Science, University of Trento, Via Sommarive 9, 38123 Trento, Italy.

E-mail addresses

Keiichi Mochida keiichi.mochida@riken.jp

Satoru Koda s-kouda@math.kyushu-u.ac.jp

Komaki Inoue komaki.inoue@riken.jp

Takashi Hirayama hira-t@okayama-u.ac.jp

Shojiro Tanaka sh-tanaka@hue.ac.jp

Ryuei Nishii nishii@math.kyushu-u.ac.jp

Farid Melgani farid.melgani@unitn.it

***Corresponding author**

Keiichi Mochida, Cellulose Production Research Team, Biomass Engineering Research Division, RIKEN Center for Sustainable Resource Science, 1-7-22 Suehiro-cho, Tsurumi-ku, Yokohama, Kanagawa 230-0045, Japan. Tel: +81-45-503-9111, E-mail: keiichi.mochida@riken.jp

Abstract

Employing computer vision to extract useful information from images and videos is becoming a key technique in identifying phenotypic changes in plants. Recent advances in image analysis empowered by machine learning-based techniques, including convolutional neural network-based modeling, have expanded their application to object segmentation and classification tasks, which assist high-throughput plant phenotyping. Combinatorial use of multiple sensors to acquire various spectra allow us to noninvasively obtain series of datasets along with developments and physiological responses throughout a plant's life. Automated phenotyping platforms accelerate the elucidation of gene functions that associate with traits in model plants under controlled conditions. Remote sensing techniques with unmanned vehicles and tractors are also emerging for large-scale field phenotyping for crop breeding, as well as precision agriculture. From these perspectives, we review the emerging aspects of computer vision that are rapidly blooming for automated plant phenotyping.

Keywords

Machin learning, Deep neural network, Unmanned aerial vehicles (UAV), Noninvasive plant phenotyping, Hyperspectral camera

Background

Computer vision that extracts useful information from images and videos is rapidly becoming an essential technique in plant phenomics [1]. Phenomics approaches to plant science aim to identify the relationships between genetic diversities and phenotypic traits in plant species using noninvasive and high-throughput measurements of quantitative parameters that reflect traits and physiological states throughout a plant's life [2]. Recent advances in DNA sequencing technologies have enabled us to rapidly acquire a map of genomic variation at a population scale [3, 4]. Combining high-throughput analytical platforms for DNA sequencing and plant phenotyping has provided opportunities for exploring genetic factors for complex quantitative traits in plants, such as growth, environmental stress tolerance, disease resistance [5] and yield, by mapping genotypes to phenotypes using statistical genetics methods such as quantitative trait locus (QTL) analysis, genome-wide association study (GWAS) [6]. Moreover, a model of the relationship between the genotype–phenotype map of individuals in a breeding population can be used to compute genome-estimated breeding values (GEBVs) to select the best parents for new crosses in genomic selection (GS) in crop breeding [7, 8]. Thus, high-throughput phenotyping aided by computer vision with various sensors and algorithms for imagery analysis will play a crucial role for crop improvement concerning to the scenarios in population demography and climate change [9].

In this context, unmanned aerial vehicles (UAVs), also known as drones, can play an

important role for data acquisition thanks to their interesting features. UAVs are pilotless aircrafts that can be launched and steered remotely in an autonomous or a semiautonomous manner, notwithstanding the fact that they can be maneuvered precisely at various speed, orientation and altitude levels, which suits them to various applications. Moreover, they can be deployed much faster, removing thereby the need for a laborious setting in advance. They are less costly to operate (i.e., in terms of manufacturing, maintenance and power consumption alike), and allow acquiring highly qualitative data (e.g., extremely high-resolution imagery) since they can be flown at low attitudes. One of the areas that has been benefiting from UAVs is crop phenotyping thanks to their field-friendly property. In this respect, the review that was carried out in [10] surveys comprehensively the late advances on UAV-oriented crop phenotyping and related sensors. In brief, recalling the lack of convenient methods (i.e., normally manual) in plant phenotyping since they are rather time consuming, laborious, and often costly, the paper provides an up-to-date survey of recent contributions where UAVs have been promoted as a promising alternative to mitigate the earlier gaps. In this respect, several phenotyping UAV platforms are envisioned such blimps, which are characterized by a plausible hovering ability and a load-effective property. However, blimps remain relatively slow in motion and sensitive to windy conditions. Unmanned helicopters, on the other hand, offer the advantage of carrying large and heavy sensors, which enables multitasking operations. However, besides being noisy, they are somewhat costly to run and maintain. Fixed-wing UAV is another

platform that is characterized by high velocity and long autonomy, which are essential to carry out field surveying especially when the field surface is quite large. Nevertheless, they remain short of free hover option. Multirotor UAVs are less costly, very flexible and easily customizable. However, one of their main drawbacks refer to the rather short autonomy and limited payload. Despite such shortcomings, multirotor UAVs remain the most commonly used platform. The plant phenotyping endeavor dictates not only what type of UAV platform to be adopted but also what kind of sensors suit the requirements. A wide range of sensors can be encountered in this respect, the basic ones are digital cameras that are typically adopted for quick color and/or texture-based phenotyping operations. Multispectral and hyperspectral sensors can capture richer spectral information about the plants of interest, allowing thus a more in-depth phenotyping. Beyond these latter, thermal infrared sensors offer another complementary and useful information especially when it comes to determining the response of canopy to stress. LIDAR is another form of sensors, which however has not received much attention in crop phenotyping. Synthetic aperture radar (SAR) is an imaging sensor that can acquire high resolution images, which is most useful for crop identification, monitoring and yield estimation. While each of the aforementioned sensors may be adopted depending on the phenotyping demands, a combination of two or more sensors thereof is often a valid option.

Machine learning (ML), an area of computer science in which algorithm design is improved automatically using experience, which aids typical steps of image analysis: preprocessing,

segmentation, feature extraction, and classification [11]. ML accelerates and automates image analysis, which improves throughput when handling labor-intensive sensor data. Algorithms based on deep learning, an emerging subfield of ML, often show more accurate performance compared to traditional approaches to computer vision-based tasks, including plant identification such as PlantCLEF[12]. Moreover, ML-based algorithms often provide deeper insights into discriminative features associated with outputs extracted through their training process, which may enable us to dissect complex traits and determine visual signatures related to traits in plants. These outcomes of ML offer us opportunities for revitalizing methodologies in plant phenomics to improve throughput, accuracy, and resolution (Figure 1).

In this review, we provide an overview of recent advances in computer vision-based approaches to plant phenotyping. Specifically, we highlight recent challenges in computer vision-assisted plant phenotyping for organ segmentation and species and physiological state classification, as well as their applications to large-scale phenotyping in genetic studies. We also showcase recently developed tools and resources for image analyses in plant phenotyping. Then, we discuss perspectives and opportunities for computer vision in plant phenomics.

Main text

Segmentation of plant organs

Segmentation represents a first, important and useful tool for information extraction from image data. The analysis of plant organs is particularly interested in this tool (Table 1). For instance, in [13], a method is presented for tracking, on a daily basis, the development of ear and silk in maize. Initially, based on images acquired by means of a commanded mobile camera that is placed at about 30 cm from the ear of interest, the method proceeds by selecting the plant side view that is most adequate for the detection of ear positions. Subsequently, the captured images are segmented, and the stems therein are labelled, and the ear position can be determined by observing changes in width along the stems. Validated on 60 maize hybrids, the proposed pipeline scored an accuracy of 86%. Motivated by the fact that counting maize tassels (which is deemed an important step towards keeping up with the growth of maize plants) is still carried out manually, this paper puts forth a computer vision pipeline to address the counting problem. First, a novel Maize Tassels Counting (MTC) dataset was created and manually annotated from 361 field images across China within 2010-2015. Second, a deep convolutional neural network is applied on the MTC dataset, and turned out to achieve plausible results with respect to relevant state-of-the-art, with an absolute error of 6.6 and a mean squared error of 9.6 [14]. Yield estimation is one of the critical aspects in agriculture. In this context, traditional manual crop counting remains rather limited especially when it comes to very large orchards, besides the fact that it is costly and time-consuming. This paper suggests a deep convolutional neural network (based

on the Inception-ResNet) for yield estimation, which is trained on synthetic data and tested on real data, and revealed a 91% of counting accuracy [15]. The paper in [16] details a method for accurate extraction as well as measurement of spike and grain morphometric parameters from images acquired by X-ray micro-computed tomography (μCT). The proposed method was applied to analyze the spikes from an ensemble of wheat plants exposed to high temperatures under two different water regimes. Interestingly, it was found out that temperature exhibits a negative impact on spike height and grain number. It was also noticed that grain volume growth goes against grain number under mild stress. In [17], a spike detection method in wheat plants is presented, which consists mainly of two stages. First, plant segmentation is addressed via an improved color index scheme. Next, spike detection is carried out by means of a neural network. Moreover, area and height thresholds were adopted for noise removal, which has incurred an improvement in spike detection score, which amounted to over 80%. The work in [18] puts forth a re-segmentation framework for wheat leaves. Precisely, it departs from an already segmented image and turns out an improved segmentation. The underlying idea of the proposed technique is that it relies on the shape of plant leaves and local orientations in order to assimilate details that have been missed in the a priori segmentation. The proposed method can accurately determine sharp features (e.g., leaf tips, twists and axils).

Taxonomic classification assisted by computer vision

Computer vision with ML-based algorithms has significantly accelerated and improved the accuracy

of taxonomic classification tasks. Deep neural network-based approaches have advanced remarkably and expanded their applications to myriad areas, including computer vision [19-21]. Specifically, the effectiveness of convolutional neural networks (CNNs) has attracted notice from researchers; the significant advantages of CNNs can be attributed not only to their highly accurate recognition abilities, but also to their automation of the processes of learning discriminative features. Thus, CNNs no longer follow explicit feature extraction steps, unlike handcrafted feature-based algorithms. Depending on tasks, however, handcrafted feature-based approaches can achieve more precise outcomes and are often more efficient in their computational costs than CNNs. Because Wäldchen and Mäder (2017) have thoroughly summarized the literature related to computer vision-based species identification approaches published before 2016 [22], we mainly focus on studies published since then (Table 2).

In a handcrafted feature-based approach, Wilf et al. [23] attempted to classify leaf images into labels of major groups (such as families and orders) in the taxonomic category. They used Scale-invariant feature transform (SIFT) and a sparse coding approach to extract the discriminative features of leaf shapes and venation patterns, followed by a multiclass support vector machine (SVM) classifier for grouping. A sparse representation was also used by Zhang et al. [24] as part of their process for classifying plant species from RGB color leaf images, in which they used basis vectors prechosen by Euclidian distances between a test sample and its nearest training samples. This selection step aimed to reduce the computational cost of the sparse coding, and the authors demonstrated its superiority in

identification on leaf image datasets. As a case study, a Turkish research group investigated the capability of computer vision algorithms in classifying wheat grains into bread wheat and durum wheat based on grain images captured by high-resolution cameras [25, 26]. They used two types of neural networks: a multilayer perceptron (MLP) with a single hidden layer and an adaptive neuro-fuzzy inference system (ANFIS). They selected seven discriminative grain features, incorporating aspects of shape, color, and texture, and achieved greater than 99% accuracy on the grain classification task. Another group examined two taxonomic classification tasks: the *Malva* alliance taxa and genus *Cistus* taxa [27, 28]. They acquired digital images of seeds using a flatbed scanner and extracted morphometric, colorimetric, and textural seed features, before performing taxonomic classification with stepwise linear discriminant analysis (LDA). Species identification from herbarium specimens with computer vision approaches was first presented in 2016, in which Unger et al. classified German trees into tens of classes with images of herbarium specimens photographed at a high resolution [29]. Their analytical processes were composed of preprocessing, normalization, and feature extraction with Fourier descriptors, leaf shape parameters, and vein texture, followed by SVM classification. In this study, they demonstrated the potential of computer visions for taxonomic identification even when using discolored leaf images of herbarium specimens. Using rather different data for species classification, Piiroinen et al.[30] attempted tree species identification with airborne laser scanning and hyperspectral imaging in a diverse agroforestry area in Africa, where a few exotic tree species are

dominant and most native species occur less frequently. Despite this challenge, they demonstrated that the ML-based analytical approaches using SVMs and random forests (RFs) can achieve reasonable tree species identification based on airborne-sensor images.

In the last few years, many CNN-based approaches have been developed for the taxonomic classification of plants [31, 32]. Dissection of trained artificial neural networks has shown that CNNs can hierarchically and simultaneously learn low-, mid-, and high-level features during training [31, 32]. Using a dataset of accurately annotated images of wheat lines, the authors in [33] applied a CNN-based model to perform feature location regression to identify spikes and spikelets, as well as image-level classification of wheat awns, suggesting the feasibility of employing CNN-based models in multiple tasks by coordinating their network architecture. In this study, the authors also suggested that the images of wheat in the training dataset, which were acquired using a consumer-grade 12 MP camera, can be even favorable for training the CNN-based model. A comparative assessment between CNN-based and handcrafted feature-based approaches was performed in a rice kernel classification task [34] In this assessment, the authors compared a deep CNN with *k*-nearest neighbor (kNN) algorithms and SVMs, along with handcrafted features such as a pyramid histogram of oriented gradients (HOG) and GIST, and showed that the CNN surpassed the kNN and SVM algorithms in classification accuracy.

Although CNNs usually require large amounts of data and extensive computational load and time,

transfer learning (i.e., the reuse and fine-tuning of pretrained networks for other tasks) is a promising technique for mitigating these costs [35-37]. Ghazi et al.[35] fine-tuned the three deep neural networks that performed well in the ImageNet Large-Scale Visual Recognition Challenge (ILSVRC)—AlexNet, GoogLeNet, and VGGNet—for a large classification dataset of 1000 species from PlantCLEF2015, aiming to construct a neural network model for taxonomic classification. In this study, the authors compared fine-tuning with training from scratch, and demonstrated that the fine-tuning approach had a slight edge in species identification. Carranza-Rojas et al. [36] applied a pretrained CNN to herbarium species classification. Sulc and Matas [37] utilized a pretrained 152-layer residual network model [38] and the Inception-ResNet-v2 model [39], for plant recognition “in the wild,” where views on plants or their organs differ significantly and the background is cluttered. In this study, the authors also proposed a textual feature, called Fast Features Invariant to Rotation and Scale of Texture (Ffirst), to computationally recognize bark and leaves from segmented images. They demonstrated improved recognition rates with this feature for a small computational cost. Pound et al. [40] applied CNNs to two types of identification tasks, classification and localization, with megapixel images taken by multiple cameras. In this classification task, the authors succeeded in identifying root tips and leaf-ear tips with accuracies of 98.4% and 97.3%, respectively, with deep CNNs, as well as extending the trained classifiers to the task of localizing plant root and shoot features. To provide a systematic guideline for plant image classification tasks, Rzanny et al. [41] examined combinations of parameters

in image acquisition (perspective, illumination, and background) and preprocessing steps (nonprocessed, cropped, and segmented), as well as manual efforts in these steps, and reported that images taken from top-side of leaves were most effective in processing of nondestructive leaf images. Interestingly in this study, the authors recorded leaf images using a smartphone, the iPhone 6, in diverse situations, including natural background conditions, followed by feature extraction with the pretrained ResNet-50 CNN and classification with a SVM.

Classification of plant physiological states

Computer vision-based image classification has been expanding its application to describing the developmental stages, physiological states, and quality of plants. Autonomous phenotyping systems equipped with multiple sensors for data acquisition enable us to collect information associated with internal and surface changes in plants [42-44]. Through explorations of the relations between multidimensional spectral signatures and the physiological properties of plants, we may be able to identify novel spectral markers that can reflect various plant physiological states [42, 45-47]. Moreover, noninvasive data acquisition enables us to continuously monitor phenotypic changes over time in plant life courses [48]. Therefore, computer vision-based plant phenotyping provides opportunities to identify early and fine changes in plant growth, assisting crop diagnostics in precision agriculture.

ML-based and statistical algorithms have been used to extract structural features from plant images for tasks such as tissue segmentation, growth stage classification, and of quality evaluation in

plants [49]. Multiple ML-based algorithms such as kNN, naive Bayes classifier (NBC), and SVM algorithms, have been examined in segmentation processes for detect aerial parts of plants, and it was suggested that different algorithms would be preferable for segmenting images of the visible and near infrared (NIR) spectra, respectively [50]. The bag-of-keypoints/bag-of-visual-words method is often used for computer vision-based image classification using quantized feature vectors as keypoints, which are an analogy to the bag-of-words method for text categorization using keywords [51]. In the bag-of-keypoints method, the SIFT algorithm is often used for keypoint detection and local feature description when constructing image classifiers [52, 53]. The bag-of-keypoints method was recently applied to RGB color images of wheat under field conditions, and demonstrated its ability to identify growth stages from heading to flowering [54]. Quality inspection of harvested crop grains can also be assisted by a computer-vision based approach to describe the relationship between grains' visual appearance and their qualities. A method based on omnidirectional Gaussian derivative filtering was proposed to extract visual features from images of granulated products cereal grains and applied to automated rice quality classification [55].

Computer vision-based image classification techniques have been also widely used to identify symptoms of disease in plants. It was demonstrated that hyperspectral imaging of *Pseudomonas cichorii* infected tomato plants could detect changes in infected plants prior to symptom appearance [56]. Hyperspectral imaging was also applied to detecting and quantifying downy mildew symptoms

caused by *Plasmopara viticola* in grapevine plants [57]. Recent deep learning-based techniques have innovated throughput and accuracy in detecting disease symptoms in plants. Mohanty et al. [58] demonstrated the feasibility of using a deep CNN to detect 26 diseases in 14 crop species, by fine-tuning popular pretrained deep CNN architectures such as AlexNet [59] and GoogLeNet [60] with a publicly available 54,306-image dataset of diseased and healthy plants from PlantVillage. Transfer learning was also used to train CNN models for detecting of disease symptoms in crops such as olive [61].

Computer-vision assisted gene discovery

Modern techniques in computer vision can aid digital quantification of various morphological and physiological parameters in plants, and are expected to improve the throughput and accuracy of plant phenotyping for population-scale analyses [62, 63]. Combined with recently advances in high-throughput DNA sequencing, the automated acquisition of plant phenotypic data followed by computer vision-based extraction of phenotypic features provides opportunities for genome-scale exploration of useful genes and modeling of the molecular networks underlying complex traits related to plant productivity such as growth, stress tolerance, disease resistance, and yield [9, 48, 64-66].

Large-scale mutant resources have played crucial roles in reverse genetics approaches in plants, and computer vision-assisted phenome analyses can provide new insights into gene functions and molecular networks related to plant phenotypic traits. A computer vision-based tracking approach to

organ development revealed temperature-compensated cell production rates and elongation zone length in roots through comparative image analysis of a wild type and a *phytochrome-interacting factor 4 and 5* double mutant of *Arabidopsis* [67]. A new clustering technique, nonparametric modeling (NPM), was applied to a high-throughput photosynthetic phenotype dataset and demonstrated its efficiency for discriminating *Arabidopsis* chloroplast mutant lines [68]. In rice, a large-scale T-DNA insertional mutant resource was developed and applied to phenotyping 68 traits belonging to 11 categories and 3 quantitative traits, screened by well-trained breeders under field conditions [69], which leads us to inquire whether using computer vision-based phenotyping to digitize growth patterns may bridge physiological features detected by machines and agronomically important traits observed by breeders.

Phenotyping a set of accessions provides a dataset beneficial for exploring novel interactions between genetic factors that influence productivity [70]. In several instances, automated plant phenotyping systems have been applied to characterizing the growth patterns of diverse crop accessions grown under controlled conditions. An automated plant phenotyping system, the rice automatic plant phenotyping platform (RAP), assisted also in quantifying 106 traits in a maize population composed of 167 recombinant inbred lines across 16 developmental stages, and identified 998 QTLs for all investigated traits [71]. In another study using a high-throughput phenotyping system, PhenoArch [72] represented differences in daily growth among 254 maize hybrids in different soil and

water conditions and revealed genetic loci affecting stomatal conductance through a genome-wide association study using the phenomic dataset [73]. A study using multiple sensors, such as hyperspectral, fluorescence, and thermal infrared sensors, demonstrated a time course heritability of traits found in a set of 32 maize inbred lines in greenhouse conditions [74]. These examples represent that noninvasive phenotyping, unlike destructive measurement, enables us to characterize growth trajectories to identify phenotypic differences in development and phenological responses over time that may influence eventual traits such as biomass and yield [75].

For phenotyping crops under field conditions, the combinatorial use of multiple sensors and techniques for image analysis has proven to be efficient for comprehensively identifying genetic and environmental factors related to phenotypic traits. With a dataset of 14 photosynthetic parameters and four morphological traits in a diverse rice population grown under different environments, a stepwise feature-selection approach based on linear regression models assisted in identifying physiological parameters related to the variance of biomass accumulation in rice [76]. In a study of poplar trees, unmanned aerial vehicle (UAV)-based thermal imaging of a full-sib F_2 population across water conditions showed the potential of UAV-based imaging for field phenotyping in tree genetic improvements [77]. In a genetic study of iron deficiency chlorosis using an association panel of soybeans, supervised machine leaning-based image classification allowed to identify genetic loci harboring a gene involved in iron acquisition, suggesting that computer vision-based plant

phenotyping provides a promising framework for genomic prediction in crops [78]. In sorghum, UAV-based remote sensing was used to measure plant height for genomic prediction modeling, demonstrating that UAV-based phenotyping with multiple sensors is efficient for generating datasets for genomic prediction modeling [79].

New tools and resources for plant phenomics

A robotic architecture for plant phenotyping is presented in [80]. The design of the proposed solution is composed of (i) an autonomous ground vehicle that takes charge of collecting data pertaining to individual plants, and (ii) a mobile observation tower to capture the whole orchard of interest and determine specific plants that require further inspection. Preliminary results show that the proposed solution is cost effective, reliable, versatile, and extendable In [81], a methodology for plant phenotyping in order to monitor the response of plants to stress is introduced. With the aim of inspecting the hyperspectral features of diseased plants, the paper presents a hyperspectral image 'wordification' concept, where the images are treated as text documents by means of probabilistic topic models. In turn, this enabled the automatic tracking of the growth of three foliar disease in barley. The authors in [82] present a plant phenotyping framework to evaluate growth rate of containerized tree seedlings during the precultivation phase post seed germination. Stereoscopic red-green-blue (RGB) images of the seeding were acquired by means of an optical system. Comparative study of these latter enabled the calculation of the rise of seeding height as

well as the rate of their greenness. Furthermore, height feature can be exploited to estimate seeding growth for needle-leaved plant species, whilst the greenness can be used for broad-leaved plant species. An interesting analysis of vegetation-specific crop indices acquired by a multispectral camera mounted on an unmanned aerial vehicle surveyed over a pilot trial of 30 plots is conducted in [83]. The adopted indices were exploited to estimate canopy cover and leaf area index. A significant correlation between the Normalized Difference Vegetation Index (NDVI) and the Enhanced Vegetation Index (EVI) was observed. As to assess the senescence pattern of sorghum genotypes, it was found that the Normalized Difference Red Edge (NDRE) index, which estimates leaf chlorophyll content, was most useful in characterizing the leaf area senescence features of contrasting genotypes. The work in [84] studies the application of UAVs for plant phenotyping of wheat breeding. In particular, it presents a semi-automated image processing scheme for deducing plot level data from the acquired UAV imagery, which was processed by means of a photogrammetric pipeline via image orientation and radiometric calibration in order to make up orthomosaic images. Additionally, the paper assesses the relationship between vegetation indices obtained from high spatial resolution multispectral imagery acquired with two different UAV platforms and ground-truth spectral data from hand-held spectroradiometer. A comparison of various remote sensing approaches via UAVs operated at low attitudes, against that of proximal sensing and satellite imagery is provided in [84]. On this point, two physiological features, namely

canopy temperature and vegetation index (NDVI), were availed to find out the most suitable approaches for large scale crop genetic crop improvement. Thus, findings suggest that UAV-oriented methodologies are encouraged for high-throughput phenotyping. In [85], a portable imaging system is described for root counting in soil cores in real time in the field. With respect to the correlation scored by a human operator (i.e., 0.57) with the root length density of the soil, that of the proposed system achieved 0.68. Furthermore, the automated system can cover more cores. It was 16% more labor efficient, 19% more efficient, and 12% cheaper, which paves the way for potential on-field phenotyping options.

Plant phenotyping frameworks incorporate sensors with mobility systems, such as tray conveyors [86], vehicles [87], or motorized gantries [88, 89] , to continuously capture growth and physiology data from plants. An automated plant phenotyping system, called the plant high-throughput investigator (PHI) allowed noninvasive tracking of plant growth under controlled conditions using its imaging station with various camera-based imaging units [90]. The computational pipeline for single leaf-based analysis with PHI was used to monitor leaf senescence and its progression in Arabidopsis. A high-throughput hyperspectral imaging system (HHIS) was designed for indoor phenotyping of rice plants [91] and was applied to quantifying agronomic traits based on hyperspectral signatures in a global rice collection of 529 accessions [91]. PhenoTrac 4, a mobile platform for phenotyping under field conditions that is equipped with multiple passive and active sensors, was used to perform canopy-

scale phenotyping of barley and wheat [92]. Another mobile platform, the Phenomobile system equipped with multiple sensors [93] has been investigated for its potential in field-phenotyping applications to examine agronomically important traits such as stay-green [94].

Public datasets from various frameworks will provide a playground for developing analytical methods in computer vision-based plant phenotyping. A comprehensive phenome dataset has been available in *Arabidopsis*, which will be useful as a reference image-set of growth and development in the model plant species when assessing methods in computer vision-based plant phenotyping [95]. Moreover, the importance of integrating traits, phenotypes, and gene functions based on ontologies has increased dramatically; the Plant Ontology, Plant Trait Ontology and the Plant Experimental Conditions Ontology, and the Gene Ontology can facilitate semantic integration of data and corpuses rapidly generated from plant genomics and phenomics [96].

Several software tools have been developed to aid steps of image analysis in plant phenotyping. Table 3 shows summary of software tools recently developed for plant phenotyping by image processing. Image Harvest supports morphological phenotyping of plants based on its functions for image acquisition, image correction, and plant growth assessment, which uses can be applied to Open Science Grid to process large number of big plant images for morophological phenotyping [97]. More recent Plant CV v2 [98] and Leaf-GP [99] equip with IPython Notebook (Jupyter) interface for sophisticated calculations and extensible utilities including machine learning and parallel processing.

PhenoCurve [100] is a curve fitting algorism that runs on MATLAB, which was applied to identify photosynthesis hysteresis pattern in plants. PRIMAL, Pipeline of Root Image analysis using MAchine Learning [101] provides an R-based application using the Shiny framework for image analysis of plant root systems [102]. Finally, the plant image analysis database [103] showcases 163 software tools and 22 datasets (as of April 25, 2018) for analysis of plant image datasets, aiming to provide user friendly interface to find solutions as well as promote communication between users and developers [104].

Conclusions and perspectives

In recent years, computer vision-based plant phenotyping has rapidly grown as a multidisciplinary area that integrates knowledge from plant science, ML, spectral sensing, and mechanical engineering. With large-scale plant image datasets and successful CNN-based algorithms, the tools available for computer vision-based plant phenotyping have advanced remarkably in plant recognition and taxonomic classification. Repositories for pretrained models for plant identification play significant roles in rapidly implementing models for new phenotyping frameworks through fine-tuning; moreover, they aid the further improvement of recognition accuracy in more challenging tasks, such as multilabel segmentation of multiple organs and species under natural environments. These efforts to improve accuracy, throughput, and computational costs for automated plant identification will provide the analytical basis for computer vision-based plant phenotyping beyond the capacity of human vison-based observation.

Computer vision-based plant phenotyping has already played important roles in monitoring the physiological states of plants for agricultural applications, such as disease symptoms and grain quality. Meta-analysis of the spectral signatures of crops associated with growth stage, physiological states, and environmental conditions will provide useful clues for preventive intervention in farming. Moreover, spectral signatures observed during earlier growth stages of crops, which are associated with eventual agronomic traits such as yield and quality, will be beneficial phenotypes for dissecting the interactions between genetic and environmental factors and for increasing genetic gain in crop breeding.

Assorted sensors have assisted plant phenotyping under both controlled and field conditions, and will aid our discovery of genes involved in agronomic traits and our understanding of their functions through statistical explorations of genome-phenome relations such as GWAS and phenome-wide association studies (PheWAS) [105, 106] in plants. High-throughput automated phenotyping will allow us to carry out common garden experiments with diverse genetic resources to understand the genetic bases of adaptive traits in plants [107]. Noninvasive and population-scale plant phenotyping will provide us opportunities to investigate interactions between internal and external factors related to plant growth and development, dissecting their effects of earlier life-course exposures onto later agronomic outcomes. Moreover, with the recent success of ML-based approaches in predicting individual traits in genomic prediction [108] and cohort studies [109, 110],

computer vision-based phenotyping will play significant roles not only for nowcasting but also forecasting plant traits through modeling genome–phenome relations.

Declarations

Ethics approval and consent to participate

Not applicable

Consent for publication

Not applicable

Availability of data and material

Not applicable

Competing interests

The authors declare that they have no competing interests.

Funding

The work was supported by CREST of the Japan Science and Technology Agency (JST).

Authors' contributions

K. M., R. N., and F. M. conceived the project. All authors drafted and edited the manuscript. K. M., S.

K. and K. I. edited the tables and the figure. All authors read and approved the final manuscript.

Acknowledgements

The authors gratefully thank to Nobuko Kimura and Kyoko Ikebe for their excellent assistance in this study.

References

1. Tardieu F, Cabrera-Bosquet L, Pridmore T and Bennett M. Plant Phenomics, From Sensors to Knowledge. Curr Biol. 2017;27 15:R770-R83.
2. Crisp PA, Ganguly D, Eichten SR, Borevitz JO and Pogson BJ. Reconsidering plant memory: Intersections between stress recovery, RNA turnover, and epigenetics. Sci Adv. 2016;2 2:e1501340.
3. Onda Y and Mochida K. Exploring Genetic Diversity in Plants Using High-Throughput Sequencing Techniques. Curr Genomics. 2016;17 4:358-67.
4. Sharma TR, Devanna BN, Kiran K, Singh PK, Arora K, Jain P, et al. Status and Prospects of Next Generation Sequencing Technologies in Crop Plants. Curr Issues Mol Biol. 2018;27:1-36.
5. Simko I, Jimenez-Berni JA and Sirault XR. Phenomic Approaches and Tools for Phytopathologists. Phytopathology. 2017;107 1:6-17.
6. Bazakos C, Hanemian M, Trontin C, Jimenez-Gomez JM and Loudet O. New Strategies and Tools in Quantitative Genetics: How to Go from the Phenotype to the Genotype. Annu Rev Plant Biol. 2017;68:435-55.
7. Crossa J, Perez-Rodriguez P, Cuevas J, Montesinos-Lopez O, Jarquin D, de Los Campos G, et al. Genomic Selection in Plant Breeding: Methods, Models, and Perspectives. Trends Plant Sci. 2017;22 11:961-75.
8. Cabrera-Bosquet L, Crossa J, von Zitzewitz J, Serret MD and Araus JL. High-throughput phenotyping and genomic selection: the frontiers of crop breeding converge. J Integr Plant Biol. 2012;54 5:312-20.
9. Araus JL, Kefauver SC, Zaman-Allah M, Olsen MS and Cairns JE. Translating High-Throughput Phenotyping into Genetic Gain. Trends in Plant Science. 2018;23 5:451-66.
10. Yang G, Liu J, Zhao C, Li Z, Huang Y, Yu H, et al. Unmanned Aerial Vehicle Remote Sensing for Field-Based Crop Phenotyping: Current Status and Perspectives. Front Plant Sci. 2017;8.
11. Perez-Sanz F, Navarro PJ and Egea-Cortines M. Plant phenomics: an overview of image acquisition technologies and image data analysis algorithms. Gigascience. 2017;6 11:1-18.
12. Department of Information Studies UoS: ImageCLEF. http://www.imageclef.org/lifeclef/2017/plant (2003). Accessed 11 June 2018.
13. Brichet N, Fournier C, Turc O, Strauss O, Artzet S, Pradal C, et al. A robot-assisted imaging pipeline for tracking the growths of maize ear and silks in a high-throughput phenotyping platform. Plant Methods. 2017;13 1:96.
14. Lu H, Cao Z, Xiao Y, Zhuang B and Shen C. TasselNet: counting maize tassels in the wild via local counts regression network. Plant Methods. 2017;13.
15. Rahnemoonfar M and Sheppard C. Deep Count: Fruit Counting Based on Deep Simulated

Learning. Sensors (Basel). 2017;17 4.

16. Hughes N, Askew K, Scotson CP, Williams K, Sauze C, Corke F, et al. Non-destructive, high-content analysis of wheat grain traits using X-ray micro computed tomography. Plant Methods. 2017;13.

17. Li QY, Cai JH, Berger B, Okamoto M and Miklavcic SJ. Detecting spikes of wheat plants using neural networks with Laws texture energy. Plant Methods. 2017;13.

18. Chopin J, Laga H and Miklavcic SJ. A Hybrid Approach for Improving Image Segmentation: Application to Phenotyping of Wheat Leaves. Plos One. 2016;11 12.

19. LeCun Y, Bengio Y and Hinton G. Deep learning. Nature. 2015;521 7553:436-44.

20. Kriegeskorte N. Deep Neural Networks: A New Framework for Modeling Biological Vision and Brain Information Processing. Annu Rev Vis Sc. 2015;1:417-46.

21. Sharma P and Singh A. Era of deep neural networks: A review. In: *International Conference on Computing, Communication and Networking Technologies (ICCCNT)* Delhi, India, 2017.

22. Wäldchen J and Mäder P. Plant Species Identification Using Computer Vision Techniques: A Systematic Literature Review. Archives of Computational Methods in Engineering. 2017.

23. Wilf P, Zhang SP, Chikkerur S, Little SA, Wing SL and Serre T. Computer vision cracks the leaf code. P Natl Acad Sci USA. 2016;113 12:3305-10.

24. Zhang SW, Wang H and Huang WZ. Two-stage plant species recognition by local mean clustering and Weighted sparse representation classification. Cluster Comput. 2017;20 2:1517-25.

25. Sabanci K, Kayabasi A and Toktas A. Computer vision-based method for classification of wheat grains using artificial neural network. J Sci Food Agr. 2017;97 8:2588-93.

26. Sabanci K, Toktas A and Kayabasi A. Grain classifier with computer vision using adaptive neuro-fuzzy inference system. J Sci Food Agr. 2017;97 12:3994-4000.

27. Lo Bianco M, Grillo O, Escobar Garcia P, Mascia F, Venora G and Bacchetta G. Morpho-colorimetric characterisation of Malva alliance taxa by seed image analysis. Plant Biol (Stuttg). 2017;19 1:90-8.

28. Lo Bianco M, Grillo O, Canadas E, Venora G and Bacchetta G. Inter- and intraspecific diversity in Cistus L. (Cistaceae) seeds, analysed with computer vision techniques. Plant Biology. 2017;19 2:183-90.

29. Unger J, Merhof D and Renner S. Computer vision applied to herbarium specimens of German trees: testing the future utility of the millions of herbarium specimen images for automated identification. Bmc Evol Biol. 2016;16.

30. Piiroinen R, Heiskanen J, Maeda E, Viinikka A and Pellikka P. Classification of Tree Species in a Diverse African Agroforestry Landscape Using Imaging Spectroscopy and Laser Scanning. Remote Sens-Basel. 2017;9 9.

31. Lee SH, Chan CS, Mayo SJ and Remagnino P. How deep learning extracts and learns leaf features

for plant classification. Pattern Recogn. 2017;71:1-13.

32. Barre P, Stover BC, Muller KF and Steinhage V. LeafNet: A computer vision system for automatic plant species identification. Ecol Inform. 2017;40:50-6.

33. Pound MP, Atkinson JA, Wells DM, Pridmore TP and French AP. Deep Learning for Multi-Task Plant Phenotyping. In: *International Conference on Computer Vision (ICCV)* Venice, Italy, 2017.

34. Lin P, Li XL, Chen YM and He Y. A Deep Convolutional Neural Network Architecture for Boosting Image Discrimination Accuracy of Rice Species. Food Bioprocess Tech. 2018;11 4:765-73.

35. Ghazi MM, Yanikoglu B and Aptoula E. Plant identification using deep neural networks via optimization of transfer learning parameters. Neurocomputing. 2017;235:228-35.

36. Carranza-Rojas J, Goeau H, Bonnet P, Mata-Montero E and Joly A. Going deeper in the automated identification of Herbarium specimens. Bmc Evol Biol. 2017;17:1-14.

37. Sulc M and Matas J. Fine-grained recognition of plants from images. Plant Methods. 2017;13.

38. He K, Zhang X, Ren S and Sun J. Deep Residual Learning for Image Recognition. In: *Computer Vision and Pattern Recognition (CVPR)* Las Vegas, NV, USA, 2016.

39. Szegedy C, Ioffe S, Vanhoucke V and Alemi A. Inception-v4, Inception-ResNet and the Impact of Residual Connections on Learning. In: *Proceedings of the Thirty-First AAAI Conference on Artificial Intelligence* 2016.

40. Pound MP, Atkinson JA, Townsend AJ, Wilson MH, Griffiths M, Jackson AS, et al. Deep machine learning provides state-of-the-art performance in image-based plant phenotyping. Gigascience. 2017;6 10:1–10.

41. Rzanny M, Seeland M, Waldchen J and Mader P. Acquiring and preprocessing leaf images for automated plant identification: understanding the tradeoff between effort and information gain. Plant Methods. 2017;13.

42. Singh A, Ganapathysubramanian B, Singh AK and Sarkar S. Machine Learning for High-Throughput Stress Phenotyping in Plants. Trends in Plant Science. 2016;21 2:110-24.

43. Mahlein AK. Plant Disease Detection by Imaging Sensors - Parallels and Specific Demands for Precision Agriculture and Plant Phenotyping. Plant Dis. 2016;100 2:241-51.

44. Liew OW, Chong PC, Li B and Asundi AK. Signature Optical Cues: Emerging Technologies for Monitoring Plant Health. Sensors (Basel). 2008;8 5:3205-39.

45. Maimaitiyiming M, Ghulam A, Bozzolo A, Wilkins JL and Kwasniewski MT. Early Detection of Plant Physiological Responses to Different Levels of Water Stress Using Reflectance Spectroscopy. Remote Sens-Basel. 2017;9 7.

46. Altangerel N, Ariunbold GO, Gorman C, Alkahtani MH, Borrego EJ, Bohlmeyer D, et al. REPLY TO DONG AND ZHAO: Plant stress via Raman spectroscopy. P Natl Acad Sci USA. 2017;114 28:E5488-E90.

47. Pandey P, Ge YF, Stoerger V and Schnable JC. High Throughput In vivo Analysis of Plant Leaf Chemical Properties Using Hyperspectral Imaging. Frontiers in Plant Science. 2017;8.

48. Shakoor N, Lee S and Mockler TC. High throughput phenotyping to accelerate crop breeding and monitoring of diseases in the field. Curr Opin Plant Biol. 2017;38:184-92.

49. Blasco J, Munera S, Aleixos N, Cubero S and Molto E. Machine Vision-Based Measurement Systems for Fruit and Vegetable Quality Control in Postharvest. Adv Biochem Eng Biotechnol. 2017;161:71-91.

50. Navarro PJ, Perez F, Weiss J and Egea-Cortines M. Machine Learning and Computer Vision System for Phenotype Data Acquisition and Analysis in Plants. Sensors (Basel). 2016;16 5.

51. Zhu QQ, Zhong YF, Zhao B, Xia GS and Zhang LP. Bag-of-Visual-Words Scene Classifier With Local and Global Features for High Spatial Resolution Remote Sensing Imagery. Ieee Geosci Remote S. 2016;13 6:747-51.

52. Wang JY, Li YP, Zhang Y, Wang C, Xie HL, Chen GL, et al. Bag-of-Features Based Medical Image Retrieval via Multiple Assignment and Visual Words Weighting. Ieee T Med Imaging. 2011;30 11:1996-2011.

53. Inoue N and Shinoda K. Fast Coding of Feature Vectors Using Neighbor-to-Neighbor Search. Ieee T Pattern Anal. 2016;38 6:1170-84.

54. Sadeghi-Tehran P, Sabermanesh K, Virlet N and Hawkesford MJ. Automated Method to Determine Two Critical Growth Stages of Wheat: Heading and Flowering. Front Plant Sci. 2017;8:252.

55. Liu JP, Tang ZH, Zhang J, Chen Q, Xu PF and Liu WZ. Visual Perception-Based Statistical Modeling of Complex Grain Image for Product Quality Monitoring and Supervision on Assembly Production Line. Plos One. 2016;11 3.

56. Rajendran DK, Park E, Nagendran R, Hung NB, Cho BK, Kim KH, et al. Visual Analysis for Detection and Quantification of Pseudomonas cichorii Disease Severity in Tomato Plants. Plant Pathology J. 2016;32 4:300-10.

57. Oerke EC, Herzog K and Toepfer R. Hyperspectral phenotyping of the reaction of grapevine genotypes to Plasmopara viticola. J Exp Bot. 2016;67 18:5529-43.

58. Mohanty SP, Hughes DP and Salathe M. Using Deep Learning for Image-Based Plant Disease Detection. Frontiers in Plant Science. 2016;7.

59. Krizhevsky A, Sutskever I and Hinton GE. ImageNet Classification with Deep Convolutional Neural Networks. Commun Acm. 2017;60 6:84-90.

60. Szegedy C, Liu W, Jia Y, Sermanet P, Reed S and Anguelov D. Going deeper with convolutions. In: *Proceedings of the IEEE Conference on Computer Vision and Pattern Recognition* 2015.

61. Cruz AC, Luvisi A, De Bellis L and Ampatzidis Y. X-FIDO: An Effective Application for Detecting Olive Quick Decline Syndrome with Deep Learning and Data Fusion. Front Plant Sci.

2017;8:1741.

62. Ghanem ME, Marrou H and Sinclair TR. Physiological phenotyping of plants for crop improvement. Trends in Plant Science. 2015;20 3:139-44.

63. Araus JL and Cairns JE. Field high-throughput phenotyping: the new crop breeding frontier. Trends in Plant Science. 2014;19 1:52-61.

64. Fernandez MGS, Bao Y, Tang L and Schnable PS. A High-Throughput, Field-Based Phenotyping Technology for Tall Biomass Crops. Plant Physiology. 2017;174 4:2008-22.

65. Valliyodan B, Ye H, Song L, Murphy M, Shannon JG and Nguyen HT. Genetic diversity and genomic strategies for improving drought and waterlogging tolerance in soybeans. J Exp Bot. 2017;68 8:1835-49.

66. Chen D, Shi R, Pape JM, Neumann K, Arend D, Graner A, et al. Predicting plant biomass accumulation from image-derived parameters. Gigascience. 2018;7 2.

67. Yang X, Dong G, Palaniappan K, Mi G and Baskin TI. Temperature-compensated cell production rate and elongation zone length in the root of Arabidopsis thaliana. Plant Cell Environ. 2017;40 2:264-76.

68. Gao Q, Ostendorf E, Cruz JA, Jin R, Kramer DM and Chen J. Inter-functional analysis of high-throughput phenotype data by non-parametric clustering and its application to photosynthesis. Bioinformatics. 2016;32 1:67-76.

69. Wu HP, Wei FJ, Wu CC, Lo SF, Chen LJ, Fan MJ, et al. Large-scale phenomics analysis of a T-DNA tagged mutant population. Gigascience. 2017;6 8:1-7.

70. Al-Tamimi N, Brien C, Oakey H, Berger B, Saade S, Ho YS, et al. Salinity tolerance loci revealed in rice using high-throughput non-invasive phenotyping. Nat Commun. 2016;7.

71. Zhang X, Huang C, Wu D, Qiao F, Li W, Duan L, et al. High-Throughput Phenotyping and QTL Mapping Reveals the Genetic Architecture of Maize Plant Growth. Plant Physiol. 2017;173 3:1554-64.

72. Cabrera-Bosquet L, Fournier C, Brichet N, Welcker C, Suard B and Tardieu F. High-throughput estimation of incident light, light interception and radiation-use efficiency of thousands of plants in a phenotyping platform. New Phytol. 2016;212 1:269-81.

73. Prado SA, Cabrera-Bosquet L, Grau A, Coupel-Ledru A, Millet EJ, Welcker C, et al. Phenomics allows identification of genomic regions affecting maize stomatal conductance with conditional effects of water deficit and evaporative demand. Plant Cell Environ. 2018;41 2:314-26.

74. Liang ZK, Pandey P, Stoerger V, Xu YH, Qiu YO, Ge YF, et al. Conventional and hyperspectral time-series imaging of maize lines widely used in field trials. Gigascience. 2017;7 2.

75. Mochida K, Saisho D and Hirayama T. Crop improvement using life cycle datasets acquired under field conditions. Frontiers in Plant Science. 2015;6.

76. Qu M, Zheng G, Hamdani S, Essemine J, Song Q, Wang H, et al. Leaf Photosynthetic Parameters

Related to Biomass Accumulation in a Global Rice Diversity Survey. Plant Physiol. 2017;175 1:248-58.
77. Ludovisi R, Tauro F, Salvati R, Khoury S, Mugnozza GS and Harfouche A. UAV-Based Thermal Imaging for High-Throughput Field Phenotyping of Black Poplar Response to Drought. Frontiers in Plant Science. 2017;8.
78. Zhang J, Naik HS, Assefa T, Sarkar S, Reddy RV, Singh A, et al. Computer vision and machine learning for robust phenotyping in genome-wide studies. Sci Rep. 2017;7:44048.
79. Watanabe K, Guo W, Arai K, Takanashi H, Kajiya-Kanegae H, Kobayashi M, et al. High-Throughput Phenotyping of Sorghum Plant Height Using an Unmanned Aerial Vehicle and Its Application to Genomic Prediction Modeling. Frontiers in Plant Science. 2017;8.
80. Shafiekhani A, Kadam S, Fritschi FB and DeSouza GN. Vinobot and Vinoculer: Two Robotic Platforms for High-Throughput Field Phenotyping. Sensors-Basel. 2017;17 1.
81. Wahabzada M, Mahlein AK, Bauckhage C, Steiner U, Oerke EC and Kersting K. Plant Phenotyping using Probabilistic Topic Models: Uncovering the Hyperspectral Language of Plants. Sci Rep-Uk. 2016;6.
82. Montagnoli A, Terzaghi M, Fulgaro N, Stoew B, Wipenmyr J, Ilver D, et al. Non-destructive Phenotypic Analysis of Early Stage Tree Seedling Growth Using an Automated Stereovision Imaging Method. Frontiers in Plant Science. 2016;7.
83. Potgieter AB, George-Jaeggli B, Chapman SC, Laws K, Cadavid LAS, Wixted J, et al. Multi-Spectral Imaging from an Unmanned Aerial Vehicle Enables the Assessment of Seasonal Leaf Area Dynamics of Sorghum Breeding Lines. Frontiers in Plant Science. 2017;8.
84. Haghighattalab A, Perez LG, Mondal S, Singh D, Schinstock D, Rutkoski J, et al. Application of unmanned aerial systems for high throughput phenotyping of large wheat breeding nurseries. Plant Methods. 2016;12.
85. Wasson A, Bischof L, Zwart A and Watt M. A portable fluorescence spectroscopy imaging system for automated root phenotyping in soil cores in the field. J Exp Bot. 2016;67 4:1033-43.
86. Frolov K, Fripp J, Nguyen CV, Furbank R, Bull G, Kuffner P, et al. Automated Plant and Leaf Separation: Application in 3D Meshes of Wheat Plants. In: *Digital Image Computing: Techniques and Applications (DICTA)* Gold Coast, QLD, Australia, 2016.
87. Underwood J, Wendel A, Schofield B, McMurray L and Kimber R. Efficient in-field plant phenomics for row-crops with an autonomous ground vehicle. J Field Robot. 2017;34 6:1061-83.
88. Virlet N, Sabermanesh K, Sadeghi-Tehran P and Hawkesford MJ. Field Scanalyzer: An automated robotic field phenotyping platform for detailed crop monitoring. Funct Plant Biol. 2017;44 1:143-53.
89. Reference Phenotyping System Team: TERRA-REF: ADVANVED FIELD CROP ANALYTICS. http://terraref.org. Accessed 11 on June 2018.

90. Lyu JI, Baek SH, Jung S, Chu H, Nam HG, Kim J, et al. High-Throughput and Computational Study of Leaf Senescence through a Phenomic Approach. Frontiers in Plant Science. 2017;8:1-8.

91. Feng H, Guo ZL, Yang WN, Huang CL, Chen GX, Fang W, et al. An integrated hyperspectral imaging and genome-wide association analysis platform provides spectral and genetic insights into the natural variation in rice. Sci Rep-Uk. 2017;7.

92. Barmeier G and Schmidhalter U. High-Throughput Field Phenotyping of Leaves, Leaf Sheaths, Culms and Ears of Spring Barley Cultivars at Anthesis and Dough Ripeness. Frontiers in Plant Science. 2017;8.

93. Deery D, Jimenez-Berni J, Jones H, Sirault X and Furbank R. Proximal Remote Sensing Buggies and Potential Applications for Field-Based Phenotyping. Agronomy. 2014;4 4:349-79.

94. Rebetzke GJ, Jimenez-Berni JA, Bovill WD, Deery DM and James RA. High-throughput phenotyping technologies allow accurate selection of stay-green. J Exp Bot. 2016;67 17:4919-24.

95. Arend D, Lange M, Pape JM, Weigelt-Fischer K, Arana-Ceballos F, Mucke I, et al. Quantitative monitoring of Arabidopsis thaliana growth and development using high-throughput plant phenotyping. Sci Data. 2016;3.

96. Cooper L, Meier A, Laporte MA, Elser JL, Mungall C, Sinn BT, et al. The Planteome database: an integrated resource for reference ontologies, plant genomics and phenomics. Nucleic Acids Res. 2018;46 D1:D1168-D80.

97. Knecht AC, Campbell MT, Caprez A, Swanson DR and Walia H. Image Harvest: an open-source platform for high-throughput plant image processing and analysis. J Exp Bot. 2016;67 11:3587-99.

98. Gehan MA, Fahlgren N, Abbasi A, Berry JC, Callen ST, Chavez L, et al. PlantCV v2: Image analysis software for high-throughput plant phenotyping. Peerj. 2017;5.

99. Zhou J, Applegate C, Alonso AD, Reynolds D, Orford S, Mackiewicz M, et al. Leaf-GP: an open and automated software application for measuring growth phenotypes for arabidopsis and wheat. Plant Methods. 2017;13.

100. Yang YF, Xu L, Feng ZY, Cruz JA, Savage LJ, Kramer DM, et al. PhenoCurve: capturing dynamic phenotype-environment relationships using phenomics data. Bioinformatics. 2017;33 9:1370-8.

101. Atkinson JA, Lobet G, Noll M, Meyer PE, Griffiths M and Wells DM: PRIMAL. https://plantmodelling.github.io/primal/ (2017). Accessed 11 June 2018.

102. Atkinson JA, Lobet G, Noll M, Meyer PE, Griffiths M and Wells DM. Combining semi-automated image analysis techniques with machine learning algorithms to accelerate large-scale genetic studies. Gigascience. 2017;6 10:1-7.

103. Lobet G, Draye X and Perilleux C. An online database for plant image analysis software tools. Plant Methods. 2013;9 1:38. doi:10.1186/1746-4811-9-38.

104. Lobet G. Image Analysis in Plant Sciences: Publish Then Perish. Trends in Plant Science. 2017;22

7:559-66.

105. Pendergrass SA, Brown-Gentry K, Dudek S, Frase A, Torstenson ES, Goodloe R, et al. Phenome-Wide Association Study (PheWAS) for Detection of Pleiotropy within the Population Architecture using Genomics and Epidemiology (PAGE) Network. Plos Genet. 2013;9 1.

106. Verma A and Ritchie MD. Current Scope and Challenges in Phenome-Wide Association Studies. Curr Epidemiol Rep. 2017;4 4:321-9.

107. de Villemereuil P, Gaggiotti OE, Mouterde M and Till-Bottraud I. Common garden experiments in the genomic era: new perspectives and opportunities. Heredity. 2016;116 3:249-54.

108. Liu Y and Wang D. Application of deep learning in genomic selection. In: *Bioinformatics and Biomedicine (BIBM)* Kansas City, MO, USA, 2017.

109. Kim BJ and Kim SH. Prediction of inherited genomic susceptibility to 20 common cancer types by a supervised machine-learning method. Proc Natl Acad Sci U S A. 2018;115 6:1322-7.

110. Lippert C, Sabatini R, Maher MC, Kang EY, Lee S, Arikan O, et al. Identification of individuals by trait prediction using whole-genome sequencing data. P Natl Acad Sci USA. 2017;114 38:10166-71.

111. Affonso C, Rossi ALD, Vieira FHA and de Carvalho ACPDF. Deep learning for biological image classification. Expert Syst Appl. 2017;85:114-22.

112. University of Nebraska-Lincoln: Image Harvest. http://www.plant-image-analysis.org/software/image-harvest (2013). Accessed 11 June 2018.

113. Donald Danforth Plant Science Center: PlantCV https://plantcv.danforthcenter.org (2014). Accessed 11 June 2018.

114. Crop-Phenomics-Group: Leaf-GP. http://www.plant-image-analysis.org/software/leaf-gp (2017). Accessed 11 June 2018.

115. Yang Y, Xu L, Feng Z, Cruz JA, Savage LJ, Kramer DM, et al.: PhenoCurve. http://phenomics.uky.edu/PhenoCurve/ (2016). Accessed 11 June 2018.

Figure

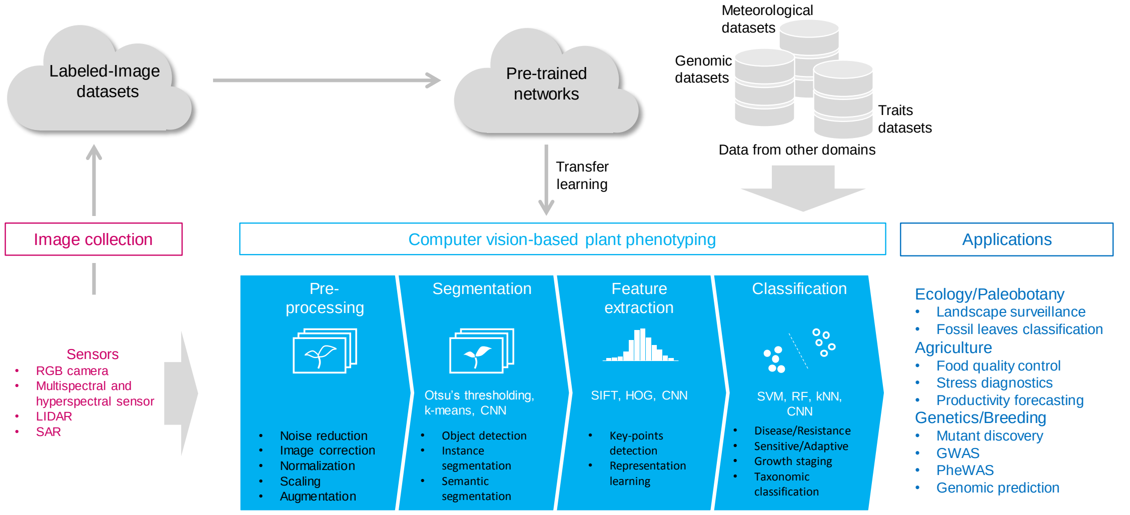

Figure 1. Schematic representation of computer vision-based plant phenotyping. Various sensors are used for collection of plant images. Large-scale collections of labeled image data are useful to design pre-trained network models. A typical step of computer vision-based image analysis consists of the following steps: pre-processing, segmentation, feature extraction, and classification. Various ML-based algorithms including CNN are applied to the steps such as segmentation, feature extraction, and classification. Pre-trained networks are often adapted to reduce computational costs through fine tuning. The classification step represents case-control phenotypes in plants; disease-resistance and sensitive-adaptive as well as morphological phenotypes; growth stages and taxonomic classification. Exploration of associations between the classification results and genetic polymorphisms, agronomic traits, and meteorological observations provides applications in areas such as ecology/paleobotany, agriculture, and genetics and breeding.

 Click here to download Table Table1.docx

Table 1. Summary of methods for plant image segmentation.

Approach	Object	Method	Reference
Model driven approaches	Fruits	CNN	[15]
	Maize ear, silk	Decision tree (top view image segmentation)	[13]
	Maize Tassel	CNN	[14]
	Wheat spike	Neural network based Laws texture energy method (spike detection)	[17]
Image driven approaches	Leaves	Hybrid algorithm	[18]
	Maize ear, silk	Mean shift + HSV threshold (side view image segmentation)	[13]
	Wheat spike, grain	Adaptive threshold + Morphology algorithm	[16]
	Wheat spike	Color indices + Morphology algorithm (foreground segmentation)	[17]

 Click here to download Table Table2.docx

Table 2. Summaries of taxonomic classification approaches.

Approach	Object	Feature extractor	Classifier	Reference
Handcrafted feature-based approach	Seed	Elliptic Fourier descriptor, Haralick's texture descriptor, morpho-colorimetric feature	LDA	[27, 28]
	Grain	Shape, color, texture features	MLP	[25]
			ANFIS	[26]
	Leaf	SIFT, sparse coding	SVM	[23]
		Fourier descriptor, leaf shapes, vein structure		[29]
		CNN		[41]
		Fast Features Invariant to Rotation and Scale of Texture (Ffirst)		[37]
		Local similarity-based-classification learning (LSCL)	LWSRC	[24]
	Bark	*Fast Features Invariant to Rotation and Scale of Texture (Ffirst)*	SVM	[37]
	Wood	Haralick´s texture descriptor	DT, kNN, MLP, NN, SVM	[111]
	Tree	Airborne imaging spectroscopy (IS) and laser scanning (ALS)	SVM, RF	[30]
CNN-based approach	Grain	CNN		[34]
	Ear, spike, spikelet			[33]
	Leaf			[31, 32, 36, 40]
	Root			[40]
	Various organs			[35-37]

Table3 Click here to download Table Table3.docx

Table 3. Summaries of tools recently developed for plant image analysis.

Name	Type	Functionalities	Reference, URL
Image Harvest	Python library, integrated with the Open Science Grid, a grid computing resources	Providing tools for image analysis to describe plant growth, morphology, and physiological responses	[97, 112]
Plant CV v2	Python library	Providing tools for image processing and normalization, leaf segmentation, landmark identification for morphometrics, and modules for machine learning.	[98, 113]
Leaf-GP	GUI-based software	Quantification of multiple growth phenotypes from large image series	[99, 114]
PhenoCurve	Matlab code	Curve fitting algorithm to identify relations between plant phenotypes and environments	[100, 115]
PRIMAL	R code	Pipeline for root image analysis in plants with random forest-based machine learning techniques.	[101, 102]

Figure1

Click here to download Figure Figure.jpg

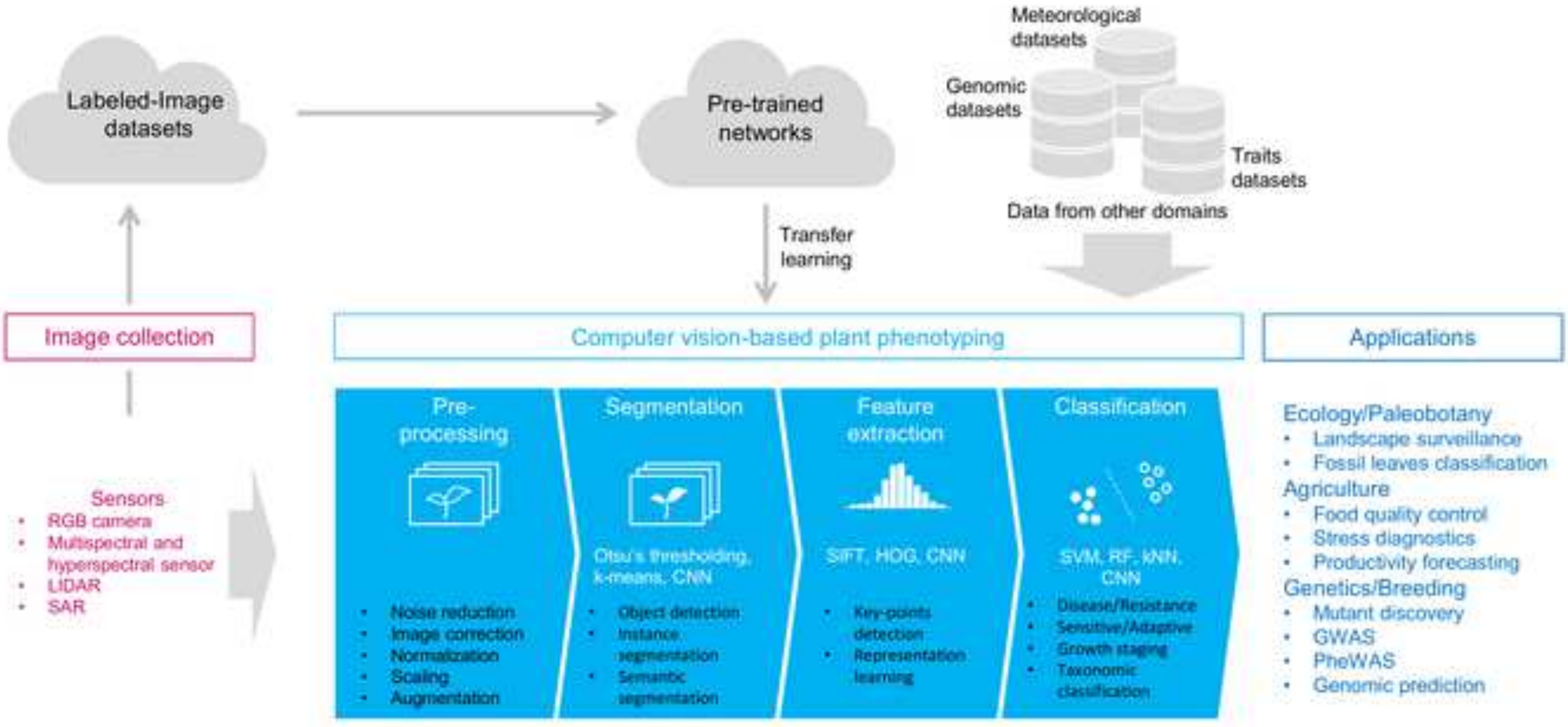

Supplement: GIGA-D-18-00215_Original_Submission.pdf [file giy153_giga-d-18-00215_original_submission.pdf]
