# Supplementary material for: Computer vision-based phenotyping for improvement of plant productivity: a machine learning perspective
Source: Gigascience. 2018 Dec 6;8(1):giy153. doi: 10.1093/gigascience/giy153 (PMC6312910; doi:10.1093/gigascience/giy153)
Supplement: GIGA-D-18-00215_Revision_1.pdf [file giy153_giga-d-18-00215_revision_1.pdf]

GigaScience

Computer vision-based phenotyping for improvement of plant productivity

--Manuscript Draft--

**Manuscript Number:**	GIGA-D-18-00215R1
**Full Title:**	Computer vision-based phenotyping for improvement of plant productivity
**Article Type:**	Review
**Funding Information:**	Core Research for Evolutional Science and Technology — Dr. Keiichi Mochida; Dr Takashi Hirayama
**Abstract:**	Employing computer vision to extract useful information from images and videos is becoming a key technique for identifying phenotypic changes in plants. In this report, we review the emerging aspects of computer vision for automated plant phenotyping. Recent advances in image analysis empowered by machine learning-based techniques, including convolutional neural network-based modeling, have expanded their application to assist high-throughput plant phenotyping. Combinatorial use of multiple sensors to acquire various spectra has allowed us to noninvasively obtain a series of datasets, including those related to the development and physiological responses of plants throughout their life. Automated phenotyping platforms accelerate the elucidation of gene functions associated with traits in model plants under controlled conditions. Remote sensing techniques with image collection platforms, such as unmanned vehicles and tractors, are also emerging for large-scale field phenotyping for crop breeding and precision agriculture. Computer vision-based phenotyping will play significant roles in both the nowcasting and forecasting of plant traits through modeling of genotype/phenotype relationships.
**Corresponding Author:**	Keiichi Mochida<br><br>JAPAN
**Corresponding Author Secondary Information:**	
**Corresponding Author's Institution:**	
**Corresponding Author's Secondary Institution:**	
**First Author:**	Keiichi Mochida
**First Author Secondary Information:**	
**Order of Authors:**	Keiichi Mochida
	Satoru Koda
	Komaki Inoue
	Takashi Hirayama
	Shojiro Tanaka
	Ryuei Nishii
	Farid Melgani
**Order of Authors Secondary Information:**	
**Response to Reviewers:**	September 6, 2018<br><br>Dear Dr. Nogoy:<br><br>We wish to express our appreciation to the editor and reviewer for their insightful comments on our manuscript. Their suggestions helped us to significantly improve the quality of the manuscript. To address the reviewers' concerns, we modified the structure of the main text, which clearly describes recent advances in computer vision-

Powered by Editorial Manager® and ProduXion Manager® from Aries Systems Corporation

based phenotyping techniques and supports machine learning-based approaches aiming to discover genes for improvement of plant productivity. We have addressed all the comments from the reviewer, as detailed in our point-by-point list below. We hope the revised manuscript is now acceptable for publication in GigaScience.

Thank you for your consideration.

Sincerely,
Keiichi Mochida
Center for Sustainable Resource Science, RIKEN

Reviewer #1: The paper is timely and interesting. However, I have some major concerns with the structure and organisation of the paper, as well as some issue with missing material. Substantial research has been done by the authors examining the literature, but I feel a stronger paper could be produced which perhaps focuses on a particular topic inside the broad remit given, or focuses on presenting to a particular audience. I hope my comments below are useful in this process.
Response: We appreciate the comments from the reviewer and agree with the reviewer's suggestions. We have reorganized the manuscript accordingly and have made substantial revisions to improve the text.

-The title is very general. Although earlier reviews in this area are cited (e.g. https://www.sciencedirect.com/science/article/pii/S1360138515002630) it is not clear where this paper is positioned in relation to these existing reviews.

Response: We appreciate this comment from the reviewer. Accordingly, we have revised the title of the manuscripts to "Computer vision-based phenotyping for improvement of plant productivity", to more clearly describe the context of our review, which focuses on recent advances in computer vision-based phenotyping techniques aiming to discover genes for improvement of plant productivity.

-Table 1 suggests a comprehensive summary of methods. Rather, these are a few sensible, but hand-picked examples of techniques. It is much better to present them as such (perhaps labeling them "Examples") rather than suggest these are the best/only approaches. It may even be better to remove the table and simply add the techniques to the literature review section.

Response: We thank the reviewer for this helpful comment and agree with the reviewer's suggestion. Accordingly, we have removed Table I and modified the section titled "Segmentation".

- There is a limited amount of narrative in the review. By which I mean, many of the papers seem to be presented one after another, without being linked together in some way. This process would help the reader form a more accurate picture of the landscape, rather than being presented with effectively a list of papers. I would recommend creating more of a narrative to structure the review. This comes back to my introductory point: what is the focus of this review, or who is it targeted towards? Knowing the answer to this will help form a strong structure to the narrative.

Response: We appreciate this comment from the reviewer. To improve the narrative in this review, we reorganized the structure of the manuscript and subheadings. Specifically, the revised structure follows the analytical process generally used in computer vision-based phenotyping described in Figure 1: image correction, preprocessing, segmentation, and classification. We also addressed recent examples of plant phenotyping that are particularly significant for plant gene discovery and highlighted the application of ML-based approaches. Next, we explained outcomes from recent attempts in plant phenotyping to identify genes related to plant traits.

-Subheadings: Some subheadings are used which is helpful, but I feel more could be used to break up the sections. This should tie in with a narrative structure mentioned in the above point.

Powered by Editorial Manager® and ProduXion Manager® from Aries Systems Corporation

Response: We thank the reviewer for this comment. To improve the structure of our manuscript, we revised the subheading of the main text as follows:
- High-throughput image collection for large-scale plant phenotyping: sensors and platforms
- Sensors
- Platforms
- Computer vision-based plant phenotyping
- Preprocessing
- Segmentation
- Feature extraction
- Classification
- Taxonomic classification
- Classification of plant physiological states
- Application of computer-vision assisted plant phenotyping for gene discovery
- Autoscreening of mutants
- Phenotyping for genetic mapping and prediction of agronomic traits
- Datasets and software tools for plant phenotyping
- Datasets
- Software tools
- Conclusions and perspectives

-Some of the review does not directly relate to machine learning, e.g. under the section starting 'L331 New tools and resources for plant phenomics' there are many items which do not seem related to machine learning, including some direct calculation of vegetative indices, e.g. NDVI. Do they belong in a machine learning review? If there is indeed a machine learning element to them, this needs to come across more clearly.

Response: We appreciate this helpful comment from the reviewer. When revising the manuscript, we have modified the text to include a section titled "High-throughput image collection for large-scale plant phenotyping" in order to discuss sensors and platforms for plant phenotyping and to introduce various indices from different sensors. We also summarize methods for data collection followed by image processing.

-Table 3 is inadequate, missing many important tools. Please see http://www.plant-image-analysis.org/ for a more comprehensive list.

Response: We thank the reviewer for this insightful suggestion. Accordingly, we revised Table 3 to describe more important software tools with ML-based algorithms; we believe this is more relevant to the context of the review. Moreover, to comprehensively evaluate the evolving ecosystem of software tools for plant image analysis, we added Figure 2 to explain the relationships between software tools in the plant-image-analysis database and target plant organs.

Some other minor comments:
L69 imagery->image, L72 aircrafts->aircraft, L76 thereby removing, L88 Do you need to also consider legislation of use for UAVs etc?, L89 *A* fixed-wing UAV..., L99 Beyond *the* latter

Response: We thank the reviewer for this comment. We have revised the manuscript and corrected these errors.

L106 I don't agree with your definition that ML is where algorithm design is improved automatically.

Response: We thank the reviewer for this comment. We corrected the definition of ML in the revised manuscript, as follows: "Machine learning (ML), an area of computer science, offers us data-driven prediction in various applications, including image analysis, which can aid typical steps of image analysis (i.e., preprocessing, segmentation, feature extraction, and classification)".

Powered by Editorial Manager® and ProduXion Manager® from Aries Systems Corporation

	L129 What is meant by a commanded mobile phone?, L134 wording: keeping up with the growth of... ?, L137 361 field images from across China in the period 2010-2015, L138 'turned out to' - informal, consider rewording, L130 are these errors rates good or bad? Always best to present the context too, so the reader can decide, L142 'This paper' - which paper? The one you are writing, or a cited one - if so, which one?<br><br>Response: We appreciate this comment from the reviewer. We have revised the text accordingly to address these issues.<br><br>L169 - 171 this is an usual decision to make for a literature review? Perhaps it needs rewording, or reconsidering? Perhaps focusing the review would mean you don't need to write this.<br><br>Response: We thank the reviewer for this comment. We revised the text at the beginning of the taxonomic classification part as follows (L359–367 in the revised manuscript): “Computer vision-based taxonomic classification plays an essential role in plant phenotyping to automatically distinguish target species for phenotyping from other plants, which is particularly important for images from real fields. Wäldchen and Mäder have thoroughly summarized the literature on computer vision-based species identification published by 2016 [22]. In recent years, because techniques for computer vision-based species identification have shown dramatically improved accuracy and expanded applications for various plant groups through hand-crafted feature-based and CNN-based approaches, we highlight studies describing plant taxonomic classification by means of these two distinctive approaches (Table 2).”<br><br>L181 to classify<br><br>Response: We appreciate this comment from the reviewer. Accordingly, we have revised the text to clarify this point (L371 in the revised manuscript).<br><br>L254 detect*ing*<br><br>Response: We thank the reviewer for this comment. We have revised the text accordingly (L439 in the revised manuscript).<br><br>L257 is bag of visual words really often used? If so I think you need a number of references supporting this statement.<br><br>Response: We appreciate this comment from the reviewer. We have added references to support the use of the phrase “bag of visual words” (L339 in the revised manuscript).
**Additional Information:**	
**Question**	**Response**
Are you submitting this manuscript to a special series or article collection?	No
**Experimental design and statistics**<br><br>Full details of the experimental design and statistical methods used should be given in the Methods section, as detailed in our Minimum Standards Reporting Checklist. Information essential to interpreting the data presented should be made available	No

in the figure legends.<br><br>Have you included all the information requested in your manuscript?	
If not, please give reasons for any omissions below.<br><br>as follow-up to "**Experimental design and statistics**<br><br>Full details of the experimental design and statistical methods used should be given in the Methods section, as detailed in our Minimum Standards Reporting Checklist. Information essential to interpreting the data presented should be made available in the figure legends.<br><br>Have you included all the information requested in your manuscript?<br><br>"	There are no experiments and statistical analyses used in this review.
**Resources**<br><br>A description of all resources used, including antibodies, cell lines, animals and software tools, with enough information to allow them to be uniquely identified, should be included in the Methods section. Authors are strongly encouraged to cite Research Resource Identifiers (RRIDs) for antibodies, model organisms and tools, where possible.<br><br>Have you included the information requested as detailed in our Minimum Standards Reporting Checklist?	Yes
**Availability of data and materials**<br><br>All datasets and code on which the conclusions of the paper rely must be either included in your submission or	No

deposited in publicly available repositories (where available and ethically appropriate), referencing such data using a unique identifier in the references and in the “Availability of Data and Materials” section of your manuscript.<br><br>Have you have met the above requirement as detailed in our Minimum Standards Reporting Checklist?	
If not, please give reasons for any omissions below.<br><br>as follow-up to "**Availability of data and materials**<br><br>All datasets and code on which the conclusions of the paper rely must be either included in your submission or deposited in publicly available repositories (where available and ethically appropriate), referencing such data using a unique identifier in the references and in the “Availability of Data and Materials” section of your manuscript.<br><br>Have you have met the above requirement as detailed in our Minimum Standards Reporting Checklist?<br><br>"	There are no datasets and code on which the conclusions of this review.

Manuscript
Click here to access/download;Manuscript;Review_with_Dr._Farid.ver.21_fo
Click here to view linked References

Computer vision-based phenotyping for improvement of plant productivity

Keiichi Mochida[1–5*], Satoru Koda[6], Komaki Inoue[1], Takashi Hirayama[3], Shojiro Tanaka[7], Ryuei Nishii[8], and Farid Melgani[9]

[1]Bioproductivity Informatics Research Team, RIKEN Center for Sustainable Resource Science, 1-7-22 Suehiro-cho, Tsurumi-ku, Yokohama, Kanagawa 230-0045, Japan

[2]Microalgae Production Control Technology Laboratory, RIKEN Baton Zone Program, RIKEN Cluster for Science, Technology and Innovation Hub, 1-7-22 Suehiro-cho, Tsurumi-ku, Yokohama, Kanagawa 230-0045, Japan

[3]Institute of Plant Science and Resources, Okayama University, 2-20-1 Chuo, Kurashiki, Okayama 710-0046, Japan

[4]Kihara Institute for Biological Research, Yokohama City University, 641-12 Maioka-cho, Totsuka-ku, Yokohama, Kanagawa 244-0813, Japan

[5]Graduate School of Nanobioscience, Yokohama City University, 22-2 Seto, Kanazawa-ku, Yokohama, Kanagawa 236-0027, Japan

[6]Graduate School of Mathematics, Kyushu University, 744 Motooka, Nishi-ku, Fukuoka 819-0395, Japan

[7]Hiroshima University of Economics, 5-37-1, Gion, Asaminami, Hiroshima-shi Hiroshima 731-0138,

Japan

[8]Institute of Mathematics for Industry, Kyushu University, 744 Motooka, Nishi-ku, Fukuoka 819-0395, Japan

[9]Department of Information Engineering and Computer Science, University of Trento, Via Sommarive 9, 38123 Trento, Italy

E-mail addresses

Keiichi Mochida keiichi.mochida@riken.jp

Satoru Koda s-kouda@math.kyushu-u.ac.jp

Komaki Inoue komaki.inoue@riken.jp

Takashi Hirayama hira-t@okayama-u.ac.jp

Shojiro Tanaka sh-tanaka@hue.ac.jp

Ryuei Nishii nishii@math.kyushu-u.ac.jp

Farid Melgani farid.melgani@unitn.it

***Corresponding author**

Keiichi Mochida, Cellulose Production Research Team, Biomass Engineering Research Division, RIKEN Center for Sustainable Resource Science, 1-7-22 Suehiro-cho, Tsurumi-ku, Yokohama,

Kanagawa 230-0045, Japan; Tel: +81-45-503-9111, E-mail: keiichi.mochida@riken.jp

Abstract

Employing computer vision to extract useful information from images and videos is becoming a key technique for identifying phenotypic changes in plants. In this report, we review the emerging aspects of computer vision for automated plant phenotyping. Recent advances in image analysis empowered by machine learning-based techniques, including convolutional neural network-based modeling, have expanded their application to assist high-throughput plant phenotyping. Combinatorial use of multiple sensors to acquire various spectra has allowed us to noninvasively obtain a series of datasets, including those related to the development and physiological responses of plants throughout their life. Automated phenotyping platforms accelerate the elucidation of gene functions associated with traits in model plants under controlled conditions. Remote sensing techniques with image collection platforms, such as unmanned vehicles and tractors, are also emerging for large-scale field phenotyping for crop breeding and precision agriculture. Computer vision-based phenotyping will play significant roles in both the nowcasting and forecasting of plant traits through modeling of genotype/phenotype relationships.

Keywords: machine learning, deep neural network, unmanned aerial vehicles, noninvasive plant

phenotyping, hyperspectral camera

Background

Computer vision that extracts useful information from plant images and videos is rapidly becoming an essential technique in plant phenomics [1]. Phenomics approaches to plant science aim to identify the relationships between genetic diversities and phenotypic traits in plant species using noninvasive and high-throughput measurements of quantitative parameters that reflect traits and physiological states throughout a plant's life [2]. Recent advances in DNA sequencing technologies have enabled us to rapidly acquire a map of genomic variations at the population scale [3, 4]. Combining high-throughput analytical platforms for DNA sequencing and plant phenotyping has provided opportunities for exploring genetic factors for complex quantitative traits in plants, such as growth, environmental stress tolerance, disease resistance [5], and yield, by mapping genotypes to phenotypes using statistical genetics methods, including quantitative trait locus (QTL) analysis and genome-wide association studies (GWASs) [6]. Moreover, a model of the relationship between the genotype/phenotype map of individuals in a breeding population can be used to compute genome-estimated breeding values to select the best parents for new crosses in genomic selection in crop breeding [7, 8]. Thus, high-throughput phenotyping aided by computer vision with various sensors and algorithms for image analysis will play a crucial role for crop yield improvement in scenarios

related to population demography and climate change [9].

Machine learning (ML), an area of computer science, offers us data-driven prediction in various applications, including image analysis, which can aid typical steps of image analysis (i.e., preprocessing, segmentation, feature extraction, and classification) [10]. ML accelerates and automates image analysis, which improves throughput when handling labor-intensive sensor data. Algorithms based on deep learning, an emerging subfield of ML, often show more accurate performance compared with traditional approaches to computer vision-based tasks, including plant identification, such as PlantCLEF [11]. Moreover, ML-based algorithms often provide deeper insights into discriminative features associated with outputs extracted through their training process, which may enable us to dissect complex traits and determine visual signatures related to traits in plants. These outcomes of ML offer us opportunities for revitalizing methodologies in plant phenomics to improve throughput, accuracy, and resolution (Figure 1).

In this review, we provide an overview of recent advances in computer vision-based plant phenotyping, which can contribute to our understanding of genotype/phenotype relationships in plants. Specifically, we summarize sensors and platforms recently developed for high-throughput plant image collection. Then, we also address recent challenges in computer vision-based plant image analysis and the typical image analysis process (e.g., segmentation, feature extraction, and classification), as well as its applications to large-scale phenotyping in genetic studies in plants, through highlighting ML-

based approaches. Moreover, we showcase datasets and software tools that are useful to plant image analysis. Then, we discuss perspectives and opportunities for computer vision in plant phenomics.

High-throughput image collection for large-scale plant phenotyping: sensors and platforms

Sensors for plant phenotyping

Various types of sensors can be encountered to acquire morphological and physiological information from plants [10] (Figure 1). The basic sensors are digital cameras that are typically adopted for quick color and/or texture-based phenotyping operations. In a previous study [12], the authors presented a plant phenotyping system for stereoscopic red-green-blue (RGB) imaging to evaluate the growth rate of tree seedlings during post-seed germination through calculation of the increase in seeding height and the rate of greenness. Multispectral and hyperspectral sensors enable us to capture richer spectral information about plants of interest, thus allowing more in-depth phenotyping. Moreover, in another previous study [13], a methodology to monitor the responses of plants to stress by inspecting the hyperspectral features of diseased plants was established, describing a hyperspectral image "wordification" concept, in which images are treated as text documents by means of probabilistic topic models, which enabled automatic tracking of the growth of three foliar disease in barley. An interesting analysis of vegetation-specific crop indices acquired by a multispectral camera mounted on an unmanned aerial vehicle (UAV) surveyed over a pilot trial of 30 plots was conducted in another prior

analysis [14]. In this study, the authors exploited multiple indices to estimate canopy cover and leaf area index; they reported that the significant correlations among the normalized difference vegetation index, enhanced vegetation index, and normalized difference red edge index, which estimates leaf chlorophyll content, were useful for characterizing leaf area senescence features of contrasting genotypes to assess the senescence patterns of sorghum genotypes. Moreover, thermal infrared sensors offer additional complementary and useful information, particularly for determining the previsual and early response of the canopy to abiotic [15] and biotic stress [16] conditions. LIDAR is another form of sensor characterized as a traditional remote sensing technique that is capable of yielding accurate three-dimensional (3D) data; this approach has been recently applied to plant phenotyping coupled with other sensors [17]. With these recent advancements, 3D reconstruction of plants enables us to identify phenotypic differences, including entire-plant and organ-level morphological changes, and combinatorial use of multiple sensors offers us opportunities to identify spectral markers associated with previsual signs of plant physiological responses.

Platforms

Plant phenotyping frameworks incorporate sensors with mobility systems, such as tray conveyors [18], aerial and ground vehicles [19], UAVs [20], and motorized gantries [21, 22], to continuously capture growth and physiology data from plants. An automated plant phenotyping system, called the plant high-throughput investigator (PHI), allowed noninvasive tracking of plant growth under controlled

conditions using an imaging station with various camera-based imaging units coupled with two growth rooms for growth of different types of plants (~200 crop plants and ~3500 *Arabidopsis*, respectively) [23]. A computational pipeline for single leaf-based analysis with PHI was used to monitor leaf senescence and its progression in *Arabidopsis*. A high-throughput hyperspectral imaging system was designed for indoor phenotyping of rice plants [24] and was applied to quantifying agronomic traits based on hyperspectral signatures in a global rice collection of 529 accessions [24]. More recently, the RIKEN Integrated Plant Phenotyping System has been used owing to its accurate quantification of *Arabidopsis* growth responses and water use efficiency in the context of various water conditions [25]. PhenoTrac 4, a mobile platform for phenotyping under field conditions that is equipped with multiple passive and active sensors, was used to perform canopy-scale phenotyping of barley and wheat [26]. Another mobile platform, the Phenomobile system equipped with multiple sensors [27], has been investigated for its potential in field-phenotyping applications to examine agronomically important traits, such as stay-green [28]. These platforms for high-throughput plant phenotyping monitor plant growth noninvasively and continuously and evaluate phenotypic differences quantitatively throughout the lifecycle at the population scale; this facilitates the identification of genetic factors associated with traits related to growth and development.

Computer vision-based plant phenotyping

In this section, we discuss recent advances in imagae analysis methodologies for plant phenotyping; these methodolodies consist of four major steps, i.e., preprocessing, segmentation, feature extraction, and classification. In each of the following subsections, we highlight ML-based approaches used in recently published literature.

Preprocessing

Preprocessing is a preliminary step of image analysis that aims to organize data properties to facilitate subsequent steps and even derive reasonable final outcomes. Particularly when we target images acquired under field conditions, unlike in controlled environments, image preprocessing contributes to enhancement of image processing quality. A simple preprocessing step is image cropping, which extracts rectangles containing target objects out of an image. Data transformation techniques, such as grayscale conversion, normalization, standardization, and contrast enhancement, are also adopted during preprocessing. Data augmentation is another example of preprocessing whose underlying goal is to increase variations in images in datasets, resulting in making pattern analysis more robust and generalized. Various techniques, such as image scaling, rotation, flipping, and noise addition, are often used for data augmentation.

Segmentation

Segmentation represents a first important step to extract information of targets from preprocessed image data by separating a set of pixels including objects of interest in images (Figure 1), enabling the identification and quantification of areas corresponding to particular organs in plants automatically. To develop a pipeline to automatically count maize tassels, a deep convolutional neural network (CNN) model, resulting from learning of the Maize Tassels Counting dataset [29], was applied, and plausible results were obtained with an absolute error of 6.6 and a mean squared error of 9.6 [29]. To automatically count tomato fruits, a deep CNN based on the Inception-ResNet was applied through training on synthetic data and tested on real data; 91% counting accuracy was obtained [30]. In addition to these model-driven approaches, various image-driven approaches have been applied for autosegmentation of plant organs. For example, in a previous study [31] in which images were acquired by X-ray micro-computed tomography, a method for accurate extraction and measurement of spike and grain morphometric parameters of wheat plants was established based on combinatorial use of adaptive threshold and morphology algorithm and applied to examine spike and grain growth of wheat exposed to high temperatures under two different water treatments. Another study [32] proposed a method for resegmentation and assimilated details that were missed in the a priori segmentation, which was useful to improve the accuracy of determination of sharp features, such as leaf tips, twists, and axils of plants. Moreover, hybrid approaches integrating model-based and image-

based approaches have been applied for segmentation of plant shape and organs. For example, in a previous study [33], a decision tree-based ML method with multiple color space and a method combining mean shift and threshold based on the hue, saturation, value color space were applied for segmentation of top and side view images in maize, yielding an accuracy of 86% in estimation of ear position in 60 maize hybrids. In wheat, researchers used an improved color index method for plant segmentation, followed by a neural network-based method with Laws texture energy; this method enabled them to detect spikes with an accuracy of over 80% [34]. Rzanny et al. [35] reported systematic guidelines for workloads of image acquisition (perspective, illumination, and background) and preprocessing (nonprocessed, cropped, and segmented) and assessed the impact of segmentation and other preprocessing techniques on recognition performances. These recent attempts to improve the accuracy of segmentation enabled us to automatically identify and quantify plant organs and evaluate the biomass and yields of fruits and grains. We were also able to improve reproducibility in phenotyping by replacing conventional human-based phenotyping, which is often time consuming and labor-intensive.

Feature extraction

Feature extraction is a step to create a set of significant and nonredundant information that can sufficiently represent images. Because pattern recognition performance in computer vision heavily depends on the quality of the extracted features, a number of approaches have been attempted in

various areas, including plant phenotyping.

Typically, features are hand-chosen based on characteristics of objects in images, such as pixel intensities, gradient, texture, and shape. For example, in a previous study [36, 37], the authors extracted features, such as shape, color, and texture (contrast, correlation, homogeny, entropy) from wheat grains to classify their accessions. Moreover, in another study [38, 39], the authors used an elliptic Fourier descriptor and the texture feature set called Haralick's texture descriptors to characterize seeds of plants for taxonomic classification. With a representative feature extraction tool, Scale Invariant Features Transforms (SIFT), which acts as an invariant feature descriptor not only to scale but also rotation, illumination, and viewpoint, Wilf et al. [40] generated codebooks for dictionary learning, and their results demonstrated the effectiveness of their approach on taxonomic classification through leaves. The bag-of-keypoints/bag-of-visual-words method, an analogy to the bag-of-words method for text categorization using keywords [41-44], has also been used as a feature representation tool in image analysis, in which the SIFT algorithm is used for keypoint detection and local feature description [45, 46]. The bag-of-keypoints method and the SIFT algorithm were applied to RGB color images of wheat under field conditions for growth stage identification [47].

Recently, CNN-based approaches have shown remarkable advancement, and their applications have been expanded to a myriad areas, including computer vision [48-50], which can automatically extract features from images and classify them. Therefore, unlike hand-chosen feature-

based algorithms, CNNs create and train classifiers without explicit feature extraction steps. Moreover, pretrained CNNs can be used as a simple feature extractor [51]. Based on these advantages of CNNs, many CNN-based strategies have been developed and are now widely used for pattern-recognition and image-classification tasks even for plant phenotyping. Notably, the authors of previous studies [52, 53] illustrated feature extraction processes based on CNNs, which learn hierarchical features through network training for taxonomic classification tasks on leaf image datasets. Recent outcomes of CNN-based classification in plant phenotyping are discussed in the following sections.

Classification

In classification steps, outcomes from the previous three steps are obtained. Here, we address classification techniques, including ML-based techniques, recently applied in plant phenotyping, highlighting two major applications: taxonomic classification and classification of plant physiological states.

Taxonomic classification Computer vision-based taxonomic classification plays an essential role in plant phenotyping to automatically distinguish target species for phenotyping from other plants, which is particularly important for images from real fields. Wäldchen and Mäder have thoroughly summarized the literature on computer vision-based species identification published by 2016 [54]. In recent years, because techniques for computer vision-based species identification have shown dramatically improved accuracy and expanded applications for various plant groups through hand-

crafted feature-based and CNN-based approaches, we highlight studies describing plant taxonomic classification by means of these two distinctive approaches (Table 1).

In a custom feature-based approach, Wilf et al. [40] attempted to classify leaf images into labels of major groups (such as families and orders) in the taxonomic category. They used SIFT and a sparse coding approach to extract the discriminative features of leaf shapes and venation patterns, followed by a multiclass support vector machine (SVM) classifier for grouping. A sparse representation was also used by Zhang et al. [55] as a part of their processes for classifying plant species from RGB color leaf images; they demonstrated the superiority of their approach in identification on leaf image datasets. As a case study, a Turkish research group investigated the capability of computer vision algorithms to classify wheat grains into bread wheat and durum wheat based on grain images captured by high-resolution cameras [36, 37]. They used two types of neural networks: a multilayer perceptron (MLP) with a single hidden layer and an adaptive neuro-fuzzy inference system (ANFIS). They selected seven discriminative grain features, incorporating aspects of shape, color, and texture, and achieved greater than 99% accuracy on the grain classification task. Another group examined two taxonomic classification tasks: the *Malva* alliance taxa and genus *Cistus* taxa [38, 39]. They acquired digital images of seeds using a flatbed scanner, extracted morphometric, colorimetric, and textural seed features, and then performed taxonomic classification with stepwise linear discriminant analysis (LDA). Species identification from herbarium specimens with computer vision approaches was first

presented in 2016, in which Unger et al. classified German trees into tens of classes with images of herbarium specimens photographed at a high resolution [56]. Their analytical processes were composed of preprocessing, normalization, and feature extraction with Fourier descriptors, leaf shape parameters, and vein texture, followed by SVM classification. In this study, they demonstrated the potential of computer visions for taxonomic identification, even when using discolored leaf images of herbarium specimens. Using rather different data for species classification, Piiroinen et al. [57] attempted tree species identifications with airborne laser scanning and hyperspectral imaging in a diverse agroforestry area in Africa, where a few exotic tree species are dominant and most native species occur less frequently. Despite this challenge, they demonstrated that ML-based analytical approaches using SVMs and random forests (RFs) could achieve reasonable tree species identification based on airborne-sensor images.

In the last few years, many CNN-based approaches have been developed for the taxonomic classification of plants [52, 53]. Using a dataset of accurately annotated images of wheat lines, the authors in a previous study [58] applied a CNN-based model to perform feature location regression to identify spikes and spikelets and carried out image-level classification of wheat awns, suggesting the feasibility of employing CNN-based models in multiple tasks by coordinating their network architecture. In this study, the authors also suggested that the images of wheat in the training dataset, which were acquired using a consumer-grade 12 MP camera, could be favorable for training the CNN-

based model. A comparative assessment between CNN-based and custom feature-based approaches was performed in a rice kernel classification task [59]. In this assessment, the authors compared a deep CNN with *k*-nearest neighbor (kNN) algorithms and SVMs, along with custom features, such as a pyramid histogram of oriented gradients and GIST, and showed that the CNN surpassed the kNN and SVM algorithms in classification accuracy.

Although CNNs usually require large amounts of data and extensive computational load and time, transfer learning (i.e., the reuse and fine-tuning of pretrained networks for other tasks) is a promising technique for mitigating these costs [60-62]. Ghazi et al. [60] fine-tuned the three deep neural networks that performed well in the ImageNet Large-Scale Visual Recognition Challenge, i.e., AlexNet [63], GoogLeNet [64], and VGGNet [65], for a large classification dataset of 1000 species from PlantCLEF2015, aiming to construct a neural network model for taxonomic classification. In this study, the authors compared fine-tuning with training from scratch and demonstrated that the fine-tuning approach had a slight edge in species identification. Carranza-Rojas et al. [61] applied a pretrained CNN to herbarium species classification. Sulc and Matas [62] utilized a pretrained 152-layer residual network model [66] and the Inception-ResNet-v2 model [67] for plant recognition in nature, in which views of plants or their organs differ significantly and in which the background is often cluttered. Moreover, the authors proposed the use of a textual feature, called Fast Features Invariant to Rotation and Scale of Texture (Ffirst), to computationally recognize bark and leaves from segmented images.

They demonstrated improved recognition rates with this feature for a small computational cost. Pound et al. [68] applied CNNs to two types of identification tasks, classification and localization, with megapixel images taken by multiple cameras. In this classification task, the authors succeeded in identifying root tips and leaf-ear tips with accuracies of 98.4% and 97.3%, respectively, with deep CNNs and extended trained classifiers for localizing plant root and shoot features. Rzanny et al. [35] summarized workloads of image acquisition and the impact of preprocessing on accuracy in image classification and concluded that images taken from the top sides of leaves were most effective for processing of nondestructive leaf images. Interestingly, in this study, the authors recorded leaf images using a smartphone (an iPhone 6) in diverse situations, including natural background conditions, followed by feature extraction with the pretrained ResNet-50 CNN and classification with a SVM.

Classification of plant physiological states The applications of computer vision-based image classification have been expanding to include description of developmental stages, physiological states, and qualities of plants (Table 2). Autonomous phenotyping systems equipped with multiple sensors for data acquisition have enabled us to collect information associated with internal and surface changes in plants [69-71]. Through exploration of the relationships between multidimensional spectral signatures and the physiological properties of plants, we may be able to identify novel spectral markers that can reflect various plant physiological states [69, 72-74]. Moreover, noninvasive data acquisition enables us to continuously monitor phenotypic changes over time in plant life courses [75]. Therefore,

computer vision-based plant phenotyping provides opportunities for early identification and detection of fine changes in plant growth, assisting crop diagnostics in precision agriculture.

ML-based and statistical algorithms have been used to extract structural features from plant images for tasks such as tissue segmentation, growth stage classification, and quality evaluation in plants [76]. Multiple ML-based algorithms, such as kNN, naive Bayes classifier, and SVM algorithms, have been examined in segmentation processes for detecting aerial parts of plants, and the findings suggested that different algorithms would be preferable for segmenting images of the visible and near infrared spectra [77]. The bag-of-keypoints method was recently applied to RGB color images of wheat under field conditions and demonstrated its ability to identify growth stages from heading to flowering [47]. Quality inspection of harvested crop grains can also be assisted by computer vision-based approaches to describe the relationships between the visual appearance and qualities of grains. A method based on omnidirectional Gaussian derivative filtering was proposed to extract visual features from images of granulated products (e.g., cereal grains) and applied to automated rice quality classification [78].

Computer vision-based image classification techniques have also been widely used to identify symptoms of disease in plants. Hyperspectral imaging was applied to detect and quantify downy mildew symptoms caused by *Plasmopara viticola* in grapevine plants [79]. Recent deep learning-based techniques have led to improvements in throughput and accuracy for detecting disease

symptoms in plants. Mohanty et al. [80] demonstrated the feasibility of using a deep CNN to detect 26 diseases in 14 crop species by fine-tuning popular pretrained deep CNN architectures, such as AlexNet [63] and GoogLeNet [64], with a publicly available 54,306-image dataset of diseased and healthy plants from PlantVillage. Transfer learning was also used to train CNN models for detecting of disease symptoms in crops, such as olives [81].

Application of computer vision-assisted plant phenotyping for gene discovery

Modern techniques in computer vision can aid digital quantification of various morphological and physiological parameters in plants and are expected to improve the throughput and accuracy of plant phenotyping for population-scale analyses [82, 83]. Combined with recent advances in high-throughput DNA sequencing, the automated acquisition of plant phenotypic data followed by computer vision-based extraction of phenotypic features provides opportunities for genome-scale exploration of useful genes and modeling of the molecular networks underlying complex traits related to plant productivity, such as growth, stress tolerance, disease resistance, and yield [9, 75, 84-86].

Autoscreening of mutants

Large-scale mutant resources have played crucial roles in reverse genetics approaches in plants, and computer vision-assisted phenotype analyses can provide new insights into gene functions and molecular networks related to traits in plants. A computer vision-based tracking approach to organ

development revealed temperature-compensated cell production rates and elongation zone lengths in roots through comparative image analysis of wild-type *Arabidopsis* and a phytochrome-interacting factor 4- and 5-double mutant of *Arabidopsis* [87]. A new clustering technique, nonparametric modeling, was applied to a high-throughput photosynthetic phenotype dataset and showed efficiency for discriminating *Arabidopsis* chloroplast mutant lines [88]. In rice, a large-scale T-DNA insertional mutant resource was developed and applied to phenotyping 68 traits belonging to 11 categories and three quantitative traits, screened by well-trained breeders under field conditions [89]. These findings led us to question whether using computer vision-based phenotyping to digitize growth patterns may bridge physiological features detected by machines and agronomically important traits observed by breeders.

Phenotyping for genetic mapping and prediction of agronomic traits

Phenotyping a set of accessions provides a dataset beneficial for exploring novel interactions between genetic factors that influence productivity [90]. In several instances, automated plant phenotyping systems have been applied for characterizing the growth patterns of diverse crop accessions grown under controlled conditions. An automated plant phenotyping system, the rice automatic plant phenotyping platform, also assisted in quantifying 106 traits in a maize population composed of 167 recombinant inbred lines across 16 developmental stages and identified 998 QTLs for all investigated traits [91]. In another study using a high-throughput phenotyping system, PhenoArch [92] represented

differences in daily growth among 254 maize hybrids in different soil and water conditions and revealed genetic loci affecting stomatal conductance through a genome-wide association study using a phenomic dataset [93]. A study using multiple sensors, such as hyperspectral, fluorescence, and thermal infrared sensors, demonstrated a time course heritability of traits found in a set of 32 maize inbred lines in greenhouse conditions [94]. These examples indicate that noninvasive phenotyping, unlike destructive measurement, enables us to characterize growth trajectories to identify phenotypic differences in development and phenological responses over time that may influence eventual traits, such as biomass and yield [95].

For phenotyping crops under field conditions, the combined use of multiple sensors and techniques for image analysis has proven to be efficient for comprehensively identifying genetic and environmental factors related to phenotypic traits. With a dataset of 14 photosynthetic parameters and four morphological traits in a diverse rice population grown under different environments, a stepwise feature-selection approach based on linear regression models assisted in identifying physiological parameters related to the variance of biomass accumulation in rice [96]. In a study of poplar trees, UAV-based thermal imaging of a full-sib F_2 population across water conditions showed the potential of UAV-based imaging for field phenotyping in tree genetic improvements [97]. In a genetic study of iron deficiency chlorosis using an association panel of soybeans, supervised machine leaning-based image classification allowed identification of genetic loci harboring a gene involved in iron

acquisition, suggesting that computer vision-based plant phenotyping provides a promising framework for genomic prediction in crops [98]. In sorghum, UAV-based remote sensing was used to measure plant height for genomic prediction modeling, demonstrating that UAV-based phenotyping with multiple sensors is efficient for generating datasets for genomic prediction modeling [99].

Datasets and software tools for plant phenotyping

Datasets

Public datasets from various platforms for plant phenotyping will provide data for developing analytical methods in computer vision-based plant phenotyping. In a recent Kaggle competition, an image dataset of approximately 960 unique plants belonging to 12 species was used to create a classifier for plant taxonomic classification from a photograph of a plant seedling [100]. In a previous study [101], the authors introduced the first dataset for computer vision-based plant phenotyping, which was made available in a separate report [102].

A comprehensive phenotype dataset is available in *Arabidopsis* and will be useful as a reference image-set for the growth and development of model plant species when assessing methods in computer vision-based plant phenotyping [103]. In maize, the datasets used in two previous studies [33, 104] are available in other reports [105, 106]. Moreover, the PlantCV web site has provided image datasets acquired in grass species, such as rice, *Setaria*, and sorghum [107]. Additionally, the

importance of integrating traits, phenotypes, and gene functions based on ontologies has increased dramatically; plant ontology, plant trait ontology, and plant experimental conditions ontology, and gene ontology can facilitate semantic integration of data and corpuses rapidly generated from plant genomics and phenomics [108].

Software tools

Various types of software tools have been established to aid steps of image analysis in plant phenotyping. The Plant Image Analysis website [109] showcases 172 software tools and 28 datasets (as of August 9, 2018) for analysis of plant image datasets, aiming to provide a user-friendly interface to find solutions and promote communication between users and developers [110, 111]. Figure 2 shows the ecosystem of software tools for plant phenotyping based on the plant image analysis database, in which software tools are connected to plant organs of an analytical target, indicating that the ecosystem is growing, particularly for images from leaves, shoots, and roots. Table 3 shows examples of software tools recently developed for plant phenotyping by image processing, which take advantage of ML-based algorithms. Leaf Necrosis Classifier supports detection of leaf areas that show necrotic symptoms with combinatorial use of MLP and self-organizing maps [112]. EasyPCC evaluates the ground coverage ratio accurately through image data acquired under field conditions and uses a pixel-based segmentation method that applies a decision-tree-based segmentation model [113]. Leaf-GP is a software tool that is used for quantification of various growth phenotypes from large image series,

applying Python-based machine learning libraries, which were used to analyze the growth of *Arabidopsis* and wheat [114]. A deep CNN-based approach was applied to develop StomataCounter for detection of stomatal pores in microscopic images [115]. Moreover, the mobile app Plantix enables diagnosis and customized options for detection of plant diseases, pests, and nutrient deficiencies to users who send a picture of a plant [116]; this approach synergistically uses a deep learning, crowd-sourced database to identify plant diseases on various crops worldwide.

Conclusions and perspectives

In recent years, computer vision-based plant phenotyping has rapidly grown as a multidisciplinary area that integrates knowledge from plant science, ML, spectral sensing, and mechanical engineering. With large-scale plant image datasets and successful CNN-based algorithms, the tools available for computer vision-based plant phenotyping have shown remarkable advancements in plant recognition and taxonomic classification. Repositories for pretrained models for plant identification play significant roles in rapidly implementing models for new phenotyping frameworks through fine-tuning; moreover, these models aid in the further improvement of recognition accuracy in more challenging tasks, such as multilabel segmentation of multiple organs and species under natural environments. These efforts to improve accuracy, throughput, and computational costs for automated plant identification will provide an analytical basis for computer vision-based plant phenotyping

beyond the capacity of human vision-based observation.

Computer vision-based plant phenotyping has already played important roles in monitoring the physiological states of plants for agricultural applications, such as disease symptoms and grain quality. Meta-analysis of the spectral signatures of crops associated with growth stage, physiological states, and environmental conditions will provide useful clues for preventive interventions in farming. Moreover, spectral signatures observed during earlier growth stages of crops, which are associated with eventual agronomic traits, such as yield and quality, will be beneficial phenotypes for dissecting the interactions between genetic and environmental factors and for increasing genetic gain in crop breeding.

Assorted sensors have assisted plant phenotyping under both controlled and field conditions, and will aid our discovery of genes involved in agronomic traits and our understanding of their functions through statistical explorations of genome-phenome relationships, such as GWASs and phenome-wide association studies [117, 118] in plants. High-throughput automated phenotyping will allow common garden experiments to be performed with diverse genetic resources in order to elucidate the genetic bases of adaptive traits in plants [119]. Noninvasive and population-scale plant phenotyping will provide us opportunities to investigate interactions between internal and external factors related to plant growth and development, dissecting the effects of earlier life-course exposures onto later agronomic outcomes. Moreover, with the recent success of ML-based

approaches in predicting individual traits in genomic prediction [120] and cohort studies [121, 122], computer vision-based phenotyping will play significant roles in both nowcasting and forecasting of plant traits through modeling genotype/phenotype relationships.

Declarations

Competing interests

The authors declare that they have no competing interests.

Funding

The work was supported by CREST of the Japan Science and Technology Agency (JST).

Authors' contributions

K.M., R.N., and F.M. conceived the project. All authors drafted and edited the manuscript. K.M., S.K., and K.I. edited the tables and the figure. All authors read and approved the final manuscript.

Acknowledgements

The authors gratefully thank to Nobuko Kimura and Kyoko Ikebe for their assistance with the preparation of this manuscript.

References

1. Tardieu F, Cabrera-Bosquet L, Pridmore T and Bennett M. Plant Phenomics, From Sensors to Knowledge. Curr Biol. 2017;27 15:R770-R83.

2. Crisp PA, Ganguly D, Eichten SR, Borevitz JO and Pogson BJ. Reconsidering plant memory: Intersections between stress recovery, RNA turnover, and epigenetics. Sci Adv. 2016;2 2:e1501340.

3. Onda Y and Mochida K. Exploring Genetic Diversity in Plants Using High-Throughput Sequencing Techniques. Curr Genomics. 2016;17 4:358-67.

4. Sharma TR, Devanna BN, Kiran K, Singh PK, Arora K, Jain P, et al. Status and Prospects of Next Generation Sequencing Technologies in Crop Plants. Curr Issues Mol Biol. 2018;27:1-36.

5. Simko I, Jimenez-Berni JA and Sirault XR. Phenomic Approaches and Tools for Phytopathologists. Phytopathology. 2017;107 1:6-17.

6. Bazakos C, Hanemian M, Trontin C, Jimenez-Gomez JM and Loudet O. New Strategies and Tools in Quantitative Genetics: How to Go from the Phenotype to the Genotype. Annu Rev Plant Biol. 2017;68:435-55.

7. Crossa J, Perez-Rodriguez P, Cuevas J, Montesinos-Lopez O, Jarquin D, de Los Campos G, et al. Genomic Selection in Plant Breeding: Methods, Models, and Perspectives. Trends Plant Sci.

2017;22 11:961-75.

8. Cabrera-Bosquet L, Crossa J, von Zitzewitz J, Serret MD and Araus JL. High-throughput phenotyping and genomic selection: the frontiers of crop breeding converge. J Integr Plant Biol. 2012;54 5:312-20.

9. Araus JL, Kefauver SC, Zaman-Allah M, Olsen MS and Cairns JE. Translating High-Throughput Phenotyping into Genetic Gain. Trends in Plant Science. 2018;23 5:451-66.

10. Perez-Sanz F, Navarro PJ and Egea-Cortines M. Plant phenomics: an overview of image acquisition technologies and image data analysis algorithms. Gigascience. 2017;6 11:1-18.

11. Department of Information Studies UoS: ImageCLEF. http://www.imageclef.org/lifeclef/2017/plant (2003). Accessed 11 June 2018.

12. Montagnoli A, Terzaghi M, Fulgaro N, Stoew B, Wipenmyr J, Ilver D, et al. Non-destructive Phenotypic Analysis of Early Stage Tree Seedling Growth Using an Automated Stereovision Imaging Method. Frontiers in Plant Science. 2016;7.

13. Wahabzada M, Mahlein AK, Bauckhage C, Steiner U, Oerke EC and Kersting K. Plant Phenotyping using Probabilistic Topic Models: Uncovering the Hyperspectral Language of Plants. Sci Rep-Uk. 2016;6.

14. Potgieter AB, George-Jaeggli B, Chapman SC, Laws K, Cadavid LAS, Wixted J, et al. Multi-Spectral Imaging from an Unmanned Aerial Vehicle Enables the Assessment of Seasonal Leaf

Area Dynamics of Sorghum Breeding Lines. Frontiers in Plant Science. 2017;8.

15. Poblete T, Ortega-Farias S and Ryu D. Automatic Coregistration Algorithm to Remove Canopy Shaded Pixels in UAV-Borne Thermal Images to Improve the Estimation of Crop Water Stress Index of a Drip-Irrigated Cabernet Sauvignon Vineyard. Sensors (Basel). 2018;18 2 doi:10.3390/s18020397.

16. Zarco-Tejada PJ, Camino C, Beck PSA, Calderon R, Hornero A, Hernandez-Clemente R, et al. Previsual symptoms of Xylella fastidiosa infection revealed in spectral plant-trait alterations. Nat Plants. 2018;4 7:432-9. doi:10.1038/s41477-018-0189-7.

17. Guo Q, Wu F, Pang S, Zhao X, Chen L, Liu J, et al. Crop 3D-a LiDAR based platform for 3D high-throughput crop phenotyping. Sci China Life Sci. 2018;61 3:328-39. doi:10.1007/s11427-017-9056-0.

18. Frolov K, Fripp J, Nguyen CV, Furbank R, Bull G, Kuffner P, et al. Automated Plant and Leaf Separation: Application in 3D Meshes of Wheat Plants. In: *Digital Image Computing: Techniques and Applications (DICTA)* Gold Coast, QLD, Australia, 2016.

19. Underwood J, Wendel A, Schofield B, McMurray L and Kimber R. Efficient in-field plant phenomics for row-crops with an autonomous ground vehicle. J Field Robot. 2017;34 6:1061-83.

20. Yang G, Liu J, Zhao C, Li Z, Huang Y, Yu H, et al. Unmanned Aerial Vehicle Remote Sensing for Field-Based Crop Phenotyping: Current Status and Perspectives. Front Plant Sci. 2017;8.

21. Virlet N, Sabermanesh K, Sadeghi-Tehran P and Hawkesford MJ. Field Scanalyzer: An automated robotic field phenotyping platform for detailed crop monitoring. Funct Plant Biol. 2017;44 1:143-53.

22. Reference Phenotyping System Team: TERRA-REF: ADVANVED FIELD CROP ANALYTICS. http://terraref.org. Accessed 11 June 2018.

23. Lyu JI, Baek SH, Jung S, Chu H, Nam HG, Kim J, et al. High-Throughput and Computational Study of Leaf Senescence through a Phenomic Approach. Frontiers in Plant Science. 2017;8:1-8.

24. Feng H, Guo ZL, Yang WN, Huang CL, Chen GX, Fang W, et al. An integrated hyperspectral imaging and genome-wide association analysis platform provides spectral and genetic insights into the natural variation in rice. Sci Rep-Uk. 2017;7.

25. Fujita M, Tanabata T, Urano K, Kikuchi S and Shinozaki K. RIPPS: A Plant Phenotyping System for Quantitative Evaluation of Growth under Controlled Environmental Stress Conditions. Plant Cell Physiol. 2018; doi:10.1093/pcp/pcy122.

26. Barmeier G and Schmidhalter U. High-Throughput Field Phenotyping of Leaves, Leaf Sheaths, Culms and Ears of Spring Barley Cultivars at Anthesis and Dough Ripeness. Frontiers in Plant Science. 2017;8.

27. Deery D, Jimenez-Berni J, Jones H, Sirault X and Furbank R. Proximal Remote Sensing Buggies and Potential Applications for Field-Based Phenotyping. Agronomy. 2014;4 4:349-79.

28. Rebetzke GJ, Jimenez-Berni JA, Bovill WD, Deery DM and James RA. High-throughput phenotyping technologies allow accurate selection of stay-green. J Exp Bot. 2016;67 17:4919-24.

29. Lu H, Cao Z, Xiao Y, Zhuang B and Shen C. TasselNet: counting maize tassels in the wild via local counts regression network. Plant Methods. 2017;13.

30. Rahnemoonfar M and Sheppard C. Deep Count: Fruit Counting Based on Deep Simulated Learning. Sensors (Basel). 2017;17 4.

31. Hughes N, Askew K, Scotson CP, Williams K, Sauze C, Corke F, et al. Non-destructive, high-content analysis of wheat grain traits using X-ray micro computed tomography. Plant Methods. 2017;13.

32. Chopin J, Laga H and Miklavcic SJ. A Hybrid Approach for Improving Image Segmentation: Application to Phenotyping of Wheat Leaves. Plos One. 2016;11 12.

33. Brichet N, Fournier C, Turc O, Strauss O, Artzet S, Pradal C, et al. A robot-assisted imaging pipeline for tracking the growths of maize ear and silks in a high-throughput phenotyping platform. Plant Methods. 2017;13 1:96.

34. Li QY, Cai JH, Berger B, Okamoto M and Miklavcic SJ. Detecting spikes of wheat plants using neural networks with Laws texture energy. Plant Methods. 2017;13.

35. Rzanny M, Seeland M, Waldchen J and Mader P. Acquiring and preprocessing leaf images for automated plant identification: understanding the tradeoff between effort and information gain.

Plant Methods. 2017;13.

36. Sabanci K, Kayabasi A and Toktas A. Computer vision-based method for classification of wheat grains using artificial neural network. J Sci Food Agr. 2017;97 8:2588-93.

37. Sabanci K, Toktas A and Kayabasi A. Grain classifier with computer vision using adaptive neuro-fuzzy inference system. J Sci Food Agr. 2017;97 12:3994-4000.

38. Lo Bianco M, Grillo O, Escobar Garcia P, Mascia F, Venora G and Bacchetta G. Morpho-colorimetric characterisation of Malva alliance taxa by seed image analysis. Plant Biol (Stuttg). 2017;19 1:90-8.

39. Lo Bianco M, Grillo O, Canadas E, Venora G and Bacchetta G. Inter- and intraspecific diversity in Cistus L. (Cistaceae) seeds, analysed with computer vision techniques. Plant Biology. 2017;19 2:183-90.

40. Wilf P, Zhang SP, Chikkerur S, Little SA, Wing SL and Serre T. Computer vision cracks the leaf code. P Natl Acad Sci USA. 2016;113 12:3305-10.

41. Zhu QQ, Zhong YF, Zhao B, Xia GS and Zhang LP. Bag-of-Visual-Words Scene Classifier With Local and Global Features for High Spatial Resolution Remote Sensing Imagery. Ieee Geosci Remote S. 2016;13 6:747-51.

42. Sonoyama S, Hirakawa T, Tamaki T, Kurita T, Raytchev B, Kaneda K, et al. Transfer learning for Bag-of-Visual words approach to NBI endoscopic image classification. Conf Proc IEEE Eng Med

Biol Soc. 2015;2015:785-8. doi:10.1109/EMBC.2015.7318479.

43. Yang W, Lu Z, Yu M, Huang M, Feng Q and Chen W. Content-based retrieval of focal liver lesions using bag-of-visual-words representations of single- and multiphase contrast-enhanced CT images. J Digit Imaging. 2012;25 6:708-19. doi:10.1007/s10278-012-9495-1.

44. Xu Y, Lin L, Hu H, Wang D, Zhu W, Wang J, et al. Texture-specific bag of visual words model and spatial cone matching-based method for the retrieval of focal liver lesions using multiphase contrast-enhanced CT images. Int J Comput Assist Radiol Surg. 2018;13 1:151-64. doi:10.1007/s11548-017-1671-9.

45. Wang JY, Li YP, Zhang Y, Wang C, Xie HL, Chen GL, et al. Bag-of-Features Based Medical Image Retrieval via Multiple Assignment and Visual Words Weighting. Ieee T Med Imaging. 2011;30 11:1996-2011.

46. Inoue N and Shinoda K. Fast Coding of Feature Vectors Using Neighbor-to-Neighbor Search. Ieee T Pattern Anal. 2016;38 6:1170-84.

47. Sadeghi-Tehran P, Sabermanesh K, Virlet N and Hawkesford MJ. Automated Method to Determine Two Critical Growth Stages of Wheat: Heading and Flowering. Front Plant Sci. 2017;8:252.

48. LeCun Y, Bengio Y and Hinton G. Deep learning. Nature. 2015;521 7553:436-44.

49. Kriegeskorte N. Deep Neural Networks: A New Framework for Modeling Biological Vision and

Brain Information Processing. Annu Rev Vis Sc. 2015;1:417-46.

50. Sharma P and Singh A. Era of deep neural networks: A review. In: *International Conference on Computing, Communication and Networking Technologies (ICCCNT)* Delhi, India, 2017.

51. Shin HC, Roth HR, Gao M, Lu L, Xu Z, Nogues I, et al. Deep Convolutional Neural Networks for Computer-Aided Detection: CNN Architectures, Dataset Characteristics and Transfer Learning. IEEE Trans Med Imaging. 2016;35 5:1285-98. doi:10.1109/TMI.2016.2528162.

52. Lee SH, Chan CS, Mayo SJ and Remagnino P. How deep learning extracts and learns leaf features for plant classification. Pattern Recogn. 2017;71:1-13.

53. Barre P, Stover BC, Muller KF and Steinhage V. LeafNet: A computer vision system for automatic plant species identification. Ecol Inform. 2017;40:50-6.

54. Wäldchen J and Mäder P. Plant Species Identification Using Computer Vision Techniques: A Systematic Literature Review. Archives of Computational Methods in Engineering. 2017.

55. Zhang SW, Wang H and Huang WZ. Two-stage plant species recognition by local mean clustering and Weighted sparse representation classification. Cluster Comput. 2017;20 2:1517-25.

56. Unger J, Merhof D and Renner S. Computer vision applied to herbarium specimens of German trees: testing the future utility of the millions of herbarium specimen images for automated identification. Bmc Evol Biol. 2016;16.

57. Piiroinen R, Heiskanen J, Maeda E, Viinikka A and Pellikka P. Classification of Tree Species in a

Diverse African Agroforestry Landscape Using Imaging Spectroscopy and Laser Scanning. Remote Sens-Basel. 2017;9 9.

58. Pound MP, Atkinson JA, Wells DM, Pridmore TP and French AP. Deep Learning for Multi-Task Plant Phenotyping. In: *International Conference on Computer Vision (ICCV)* Venice, Italy, 2017.

59. Lin P, Li XL, Chen YM and He Y. A Deep Convolutional Neural Network Architecture for Boosting Image Discrimination Accuracy of Rice Species. Food Bioprocess Tech. 2018;11 4:765-73.

60. Ghazi MM, Yanikoglu B and Aptoula E. Plant identification using deep neural networks via optimization of transfer learning parameters. Neurocomputing. 2017;235:228-35.

61. Carranza-Rojas J, Goeau H, Bonnet P, Mata-Montero E and Joly A. Going deeper in the automated identification of Herbarium specimens. Bmc Evol Biol. 2017;17:1-14.

62. Sulc M and Matas J. Fine-grained recognition of plants from images. Plant Methods. 2017;13.

63. Krizhevsky A, Sutskever I and Hinton GE. ImageNet Classification with Deep Convolutional Neural Networks. Commun Acm. 2017;60 6:84-90.

64. Szegedy C, Liu W, Jia Y, Sermanet P, Reed S and Anguelov D. Going deeper with convolutions. In: *Proceedings of the IEEE Conference on Computer Vision and Pattern Recognition* 2015.

65. Simonyan K and Zisserman A. Very Deep Convolutional Networks for Large-Scale Image Recognition. *International Conference on Learning Representations*. San Diego, CA2015.

66. He K, Zhang X, Ren S and Sun J. Deep Residual Learning for Image Recognition. In: *Computer Vision and Pattern Recognition (CVPR)* Las Vegas, NV, USA, 2016.

67. Szegedy C, Ioffe S, Vanhoucke V and Alemi A. Inception-v4, Inception-ResNet and the Impact of Residual Connections on Learning. In: *Proceedings of the Thirty-First AAAI Conference on Artificial Intelligence* 2016.

68. Pound MP, Atkinson JA, Townsend AJ, Wilson MH, Griffiths M, Jackson AS, et al. Deep machine learning provides state-of-the-art performance in image-based plant phenotyping. Gigascience. 2017;6 10:1–10.

69. Singh A, Ganapathysubramanian B, Singh AK and Sarkar S. Machine Learning for High-Throughput Stress Phenotyping in Plants. Trends in Plant Science. 2016;21 2:110-24.

70. Mahlein AK. Plant Disease Detection by Imaging Sensors - Parallels and Specific Demands for Precision Agriculture and Plant Phenotyping. Plant Dis. 2016;100 2:241-51.

71. Liew OW, Chong PC, Li B and Asundi AK. Signature Optical Cues: Emerging Technologies for Monitoring Plant Health. Sensors (Basel). 2008;8 5:3205-39.

72. Maimaitiyiming M, Ghulam A, Bozzolo A, Wilkins JL and Kwasniewski MT. Early Detection of Plant Physiological Responses to Different Levels of Water Stress Using Reflectance Spectroscopy. Remote Sens-Basel. 2017;9 7.

73. Altangerel N, Ariunbold GO, Gorman C, Alkahtani MH, Borrego EJ, Bohlmeyer D, et al. REPLY

TO DONG AND ZHAO: Plant stress via Raman spectroscopy. P Natl Acad Sci USA. 2017;114 28:E5488-E90.

74. Pandey P, Ge YF, Stoerger V and Schnable JC. High Throughput In vivo Analysis of Plant Leaf Chemical Properties Using Hyperspectral Imaging. Frontiers in Plant Science. 2017;8.

75. Shakoor N, Lee S and Mockler TC. High throughput phenotyping to accelerate crop breeding and monitoring of diseases in the field. Curr Opin Plant Biol. 2017;38:184-92.

76. Blasco J, Munera S, Aleixos N, Cubero S and Molto E. Machine Vision-Based Measurement Systems for Fruit and Vegetable Quality Control in Postharvest. Adv Biochem Eng Biotechnol. 2017;161:71-91.

77. Navarro PJ, Perez F, Weiss J and Egea-Cortines M. Machine Learning and Computer Vision System for Phenotype Data Acquisition and Analysis in Plants. Sensors (Basel). 2016;16 5.

78. Liu JP, Tang ZH, Zhang J, Chen Q, Xu PF and Liu WZ. Visual Perception-Based Statistical Modeling of Complex Grain Image for Product Quality Monitoring and Supervision on Assembly Production Line. Plos One. 2016;11 3.

79. Oerke EC, Herzog K and Toepfer R. Hyperspectral phenotyping of the reaction of grapevine genotypes to Plasmopara viticola. J Exp Bot. 2016;67 18:5529-43.

80. Mohanty SP, Hughes DP and Salathe M. Using Deep Learning for Image-Based Plant Disease Detection. Frontiers in Plant Science. 2016;7.

81. Cruz AC, Luvisi A, De Bellis L and Ampatzidis Y. X-FIDO: An Effective Application for Detecting Olive Quick Decline Syndrome with Deep Learning and Data Fusion. Front Plant Sci. 2017;8:1741.

82. Ghanem ME, Marrou H and Sinclair TR. Physiological phenotyping of plants for crop improvement. Trends in Plant Science. 2015;20 3:139-44.

83. Araus JL and Cairns JE. Field high-throughput phenotyping: the new crop breeding frontier. Trends in Plant Science. 2014;19 1:52-61.

84. Fernandez MGS, Bao Y, Tang L and Schnable PS. A High-Throughput, Field-Based Phenotyping Technology for Tall Biomass Crops. Plant Physiology. 2017;174 4:2008-22.

85. Valliyodan B, Ye H, Song L, Murphy M, Shannon JG and Nguyen HT. Genetic diversity and genomic strategies for improving drought and waterlogging tolerance in soybeans. J Exp Bot. 2017;68 8:1835-49.

86. Chen D, Shi R, Pape JM, Neumann K, Arend D, Graner A, et al. Predicting plant biomass accumulation from image-derived parameters. Gigascience. 2018;7 2.

87. Yang X, Dong G, Palaniappan K, Mi G and Baskin TI. Temperature-compensated cell production rate and elongation zone length in the root of Arabidopsis thaliana. Plant Cell Environ. 2017;40 2:264-76.

88. Gao Q, Ostendorf E, Cruz JA, Jin R, Kramer DM and Chen J. Inter-functional analysis of high-

throughput phenotype data by non-parametric clustering and its application to photosynthesis. Bioinformatics. 2016;32 1:67-76.

89. Wu HP, Wei FJ, Wu CC, Lo SF, Chen LJ, Fan MJ, et al. Large-scale phenomics analysis of a T-DNA tagged mutant population. Gigascience. 2017;6 8:1-7.

90. Al-Tamimi N, Brien C, Oakey H, Berger B, Saade S, Ho YS, et al. Salinity tolerance loci revealed in rice using high-throughput non-invasive phenotyping. Nat Commun. 2016;7.

91. Zhang X, Huang C, Wu D, Qiao F, Li W, Duan L, et al. High-Throughput Phenotyping and QTL Mapping Reveals the Genetic Architecture of Maize Plant Growth. Plant Physiol. 2017;173 3:1554-64.

92. Cabrera-Bosquet L, Fournier C, Brichet N, Welcker C, Suard B and Tardieu F. High-throughput estimation of incident light, light interception and radiation-use efficiency of thousands of plants in a phenotyping platform. New Phytol. 2016;212 1:269-81.

93. Prado SA, Cabrera-Bosquet L, Grau A, Coupel-Ledru A, Millet EJ, Welcker C, et al. Phenomics allows identification of genomic regions affecting maize stomatal conductance with conditional effects of water deficit and evaporative demand. Plant Cell Environ. 2018;41 2:314-26.

94. Liang ZK, Pandey P, Stoerger V, Xu YH, Qiu YO, Ge YF, et al. Conventional and hyperspectral time-series imaging of maize lines widely used in field trials. Gigascience. 2017;7 2.

95. Mochida K, Saisho D and Hirayama T. Crop improvement using life cycle datasets acquired under

field conditions. Frontiers in Plant Science. 2015;6.

96. Qu M, Zheng G, Hamdani S, Essemine J, Song Q, Wang H, et al. Leaf Photosynthetic Parameters Related to Biomass Accumulation in a Global Rice Diversity Survey. Plant Physiol. 2017;175 1:248-58.

97. Ludovisi R, Tauro F, Salvati R, Khoury S, Mugnozza GS and Harfouche A. UAV-Based Thermal Imaging for High-Throughput Field Phenotyping of Black Poplar Response to Drought. Frontiers in Plant Science. 2017;8.

98. Zhang J, Naik HS, Assefa T, Sarkar S, Reddy RV, Singh A, et al. Computer vision and machine learning for robust phenotyping in genome-wide studies. Sci Rep. 2017;7:44048.

99. Watanabe K, Guo W, Arai K, Takanashi H, Kajiya-Kanegae H, Kobayashi M, et al. High-Throughput Phenotyping of Sorghum Plant Height Using an Unmanned Aerial Vehicle and Its Application to Genomic Prediction Modeling. Frontiers in Plant Science. 2017;8.

100. Giselsson TM, Jørgensen RN, Jensen PK, Dyrmann M and Midtiby HS. A Public Image Database for Benchmark of Plant Seedling Classification Algorithms. 2017. https://vision.eng.au.dk/plant-seedlings-dataset/.

101. Minervini M, Fischbach A, Scharr H and Tsaftaris SA. Finely-grained annotated datasets for image-based plant phenotyping. Pattern Recogn Lett. 2016;81:80-9. doi:10.1016/j.patrec.2015.10.013.

102. Minervini M, Fischbach A, Scharr H and Tsaftaris SA: Plant Phenotyping Datasets. http://www.plant-phenotyping.org/datasets (2015). Accessed 14 August 2018.

103. Arend D, Lange M, Pape JM, Weigelt-Fischer K, Arana-Ceballos F, Mucke I, et al. Quantitative monitoring of Arabidopsis thaliana growth and development using high-throughput plant phenotyping. Sci Data. 2016;3.

104. Choudhury SD, Bashyam S, Qiu Y, Samal A and Awada T. Holistic and component plant phenotyping using temporal image sequence. Plant Methods. 2018;14:35. doi:10.1186/s13007-018-0303-x.

105. Brichet N and Cabrera-Bosquet L. Maize whole plant image dataset. 2017. http://doi.org/10.5281/zenodo.1002675.

106. Choudhury SD, Stoerger V, Samal A, Schnable JC, Liang Z and Yu J-G. Automated Vegetative Stage Phenotyping Analysis of Maize Plants using Visible Light Images DS-FEW. *KDD: Data Science for Food , Energy and Water*. San Francisco, CA2016.

107. Center DDPS: Public Image Datasets. https://plantcv.danforthcenter.org/pages/data.html (2014). Accessed 5 August 2018.

108. Cooper L, Meier A, Laporte MA, Elser JL, Mungall C, Sinn BT, et al. The Planteome database: an integrated resource for reference ontologies, plant genomics and phenomics. Nucleic Acids Res. 2018;46 D1:D1168-D80.

109. Lobet G, Draye X and Perilleux C. An online database for plant image analysis software tools. Plant Methods. 2013;9 1:38. doi:10.1186/1746-4811-9-38.

110. Lobet G. Image Analysis in Plant Sciences: Publish Then Perish. Trends in Plant Science. 2017;22 7:559-66.

111. Lobet G, Draye X and Périlleux C: Plants database. http://www.plant-image-analysis.org/dataset/plant-database. Accessed 9 August 2018.

112. Obořil M: Quantification of leaf necrosis by biologically inspired algorithms. https://lnc.proteomics.ceitec.cz/non_source_files/LNC_presentation_short.pdf (2017). Accessed 15 August 2018.

113. Guo W, Zheng B, Duan T, Fukatsu T, Chapman S and Ninomiya S. EasyPCC: Benchmark Datasets and Tools for High-Throughput Measurement of the Plant Canopy Coverage Ratio under Field Conditions. Sensors (Basel). 2017;17 4 doi:10.3390/s17040798.

114. Zhou J, Applegate C, Alonso AD, Reynolds D, Orford S, Mackiewicz M, et al. Leaf-GP: an open and automated software application for measuring growth phenotypes for arabidopsis and wheat. Plant Methods. 2017;13.

115. Fetter K, Eberhardt S, Barclay RS, Wing S and Keller SR. StomataCounter: a deep learning method applied to automatic stomatal identification and counting. bioRxiv. 2018.

116. PEAT: plantix. https://plantix.net (2017). Accessed 15 August 2018.

117. Pendergrass SA, Brown-Gentry K, Dudek S, Frase A, Torstenson ES, Goodloe R, et al. Phenome-Wide Association Study (PheWAS) for Detection of Pleiotropy within the Population Architecture using Genomics and Epidemiology (PAGE) Network. Plos Genet. 2013;9 1.

118. Verma A and Ritchie MD. Current Scope and Challenges in Phenome-Wide Association Studies. Curr Epidemiol Rep. 2017;4 4:321-9.

119. de Villemereuil P, Gaggiotti OE, Mouterde M and Till-Bottraud I. Common garden experiments in the genomic era: new perspectives and opportunities. Heredity. 2016;116 3:249-54.

120. Liu Y and Wang D. Application of deep learning in genomic selection. In: *Bioinformatics and Biomedicine (BIBM)* Kansas City, MO, USA, 2017.

121. Kim BJ and Kim SH. Prediction of inherited genomic susceptibility to 20 common cancer types by a supervised machine-learning method. Proc Natl Acad Sci U S A. 2018;115 6:1322-7.

122. Lippert C, Sabatini R, Maher MC, Kang EY, Lee S, Arikan O, et al. Identification of individuals by trait prediction using whole-genome sequencing data. P Natl Acad Sci USA. 2017;114 38:10166-71.

123. Guo W, Rage UK and Ninomiya S. Illumination invariant segmentation of vegetation for time series wheat images based on decision tree model. Computers and Electronics in Agriculture. 2013;96:58-66.

124. Crop-Phenomics-Group: Leaf-GP. http://www.plant-image-analysis.org/software/leaf-gp (2017).

Accessed 11 June 2018.

125. Shannon P, Markiel A, Ozier O, Baliga NS, Wang JT, Ramage D, et al. Cytoscape: a software environment for integrated models of biomolecular interaction networks. Genome Res. 2003;13 11:2498-504. doi:10.1101/gr.1239303.

Tables

Table 1. Examples of taxonomic classification approaches.

Approach	Object	Features/feature extractor	Classifier	Reference
Custom feature-based approach	Seed	Elliptic Fourier descriptor, Haralick's texture descriptor, morpho-colorimetric feature	LDA	[38, 39]
	Grain	Shape, color, texture features	MLP	[36]
			ANFIS	[37]
	Leaf	SIFT, sparse coding	SVM	[40]
		Fourier descriptor, leaf shapes, vein structure		[56]
		Pretrained CNN		[35]
		Fast Features Invariant to Rotation and Scale of Texture (Ffirst)		[62]

		Texture features	LWSRC	[55]
	Bark	Fast Features Invariant to Rotation and Scale of Texture (Ffirst)	SVM	[62]
	Tree	Reflectance, minimum noise fraction transformation, narrowband vegetation indices, airborne imaging spectroscopy features	SVM, RF	[57]
CNN-based approach	Grain	CNN		[59]
	Ear, spike, spikelet			[58]
	Leaf			[52, 53, 61, 68]
	Root			[68]
	Various organs			[60-62]

Table 2. Examples of approaches for classification of physiological states.

Approach	Object	Features/feature extractor	Classifier	Reference
Custom feature-based approach	Ear (growth stages)	SIFT + bag of keypoints	SVM	[47]
	Grain (quality assessment)	Weibull distribution model parameter features	SVM	[78]
	Leaf	Spectral vegetation indices	Spectral Angle Mapper	[79]
CNN-based approach	Leaf	CNN		[80, 81]

Table 3. Software tools recently developed for plant image analysis, which use machine learning-based algorithms.

Name	Algorithms	Functionalities	Reference, URL
Leaf Necrosis Classifier	Multilayer perceptron and self-organizing maps	Detection of leaf areas showing necrotic symptoms	[112]
EasyPCC	Decision-tree-based segmentation model	Quantification of ground coverage ratio from image data acquired under field conditions	[113, 123]
Leaf-GP	Python-based machine learning libraries	Quantification of multiple growth phenotypes from large image series	[114, 124]
StomataCounter	Deep CNN	Counting stomate pores	[115]
Plantix	Deep learning	Diagnosing plant diseases, pest damages and nutrient deficiencies	[116]

Figure

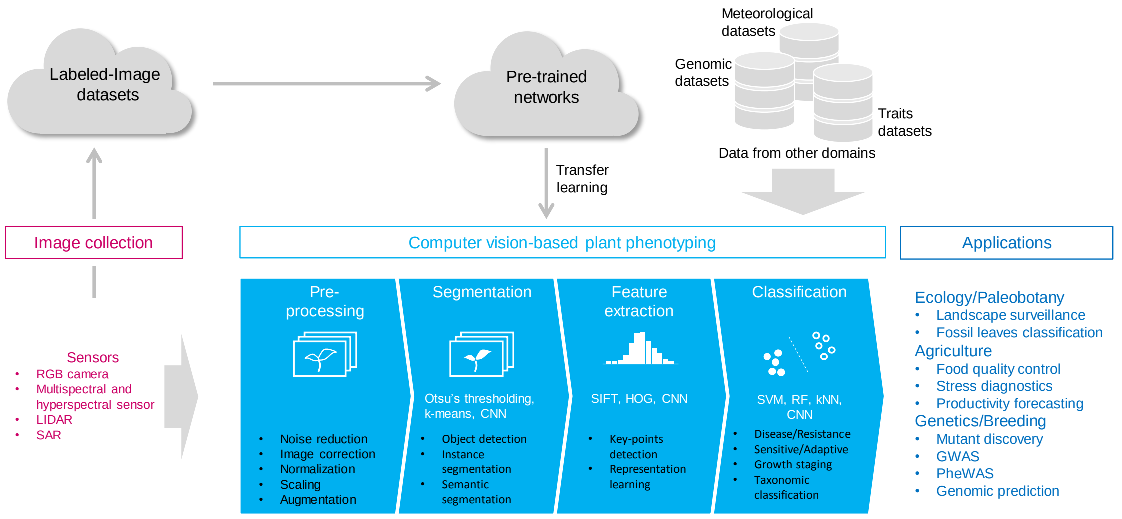

Figure 1. Schematic representation of computer vision-based plant phenotyping. Various sensors are used for collection of plant images. Large-scale collections of labeled image data are useful to design pretrained network models. A typical step of computer vision-based image analysis consists of the following steps: preprocessing, segmentation, feature extraction, and classification. Various ML-based algorithms, including CNN, are applied to the steps, such as segmentation, feature extraction, and classification. Pretrained networks are often adapted to reduce computational costs through fine tuning. The classification step represents case-control phenotypes in plants; disease-resistance, sensitive-adaptive, morphological phenotypes; growth stages; and taxonomic classification. Exploration of associations among the classification results and genetic polymorphisms, agronomic traits, and meteorological observations will expand applications to areas such as ecology/paleobotany, agriculture, and genetics and breeding.

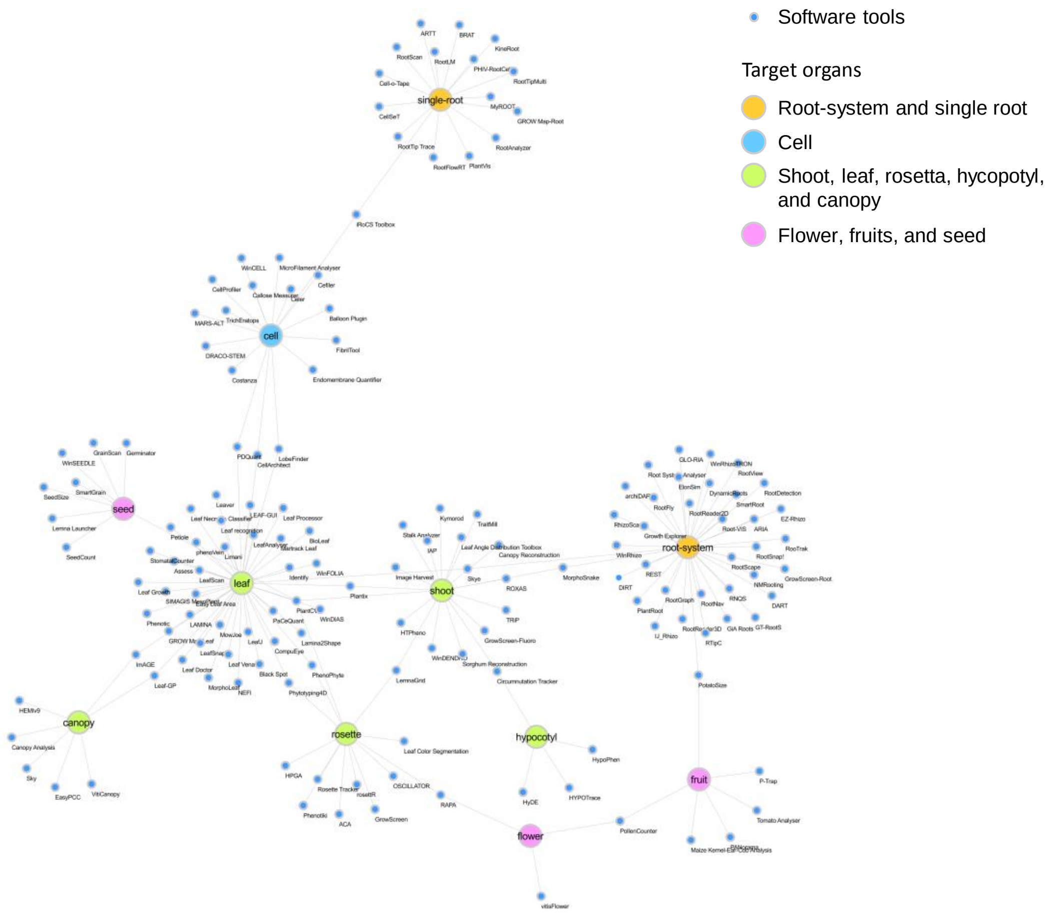

Figure 2. An ecosystem map of software tools for plant image analysis. The network-formed map consists of 169 software tools whose targets are particular plant organs based on the plant image analysis database [111]. The nodes represent the software tools and their target plant organs represented using Cytoscape 3.0 [125].

Table 1 Click here to access/download;Table;Table1.docx

Table 1. Examples of taxonomic classification approaches.

Approach	Object	Features/feature extractor	Classifier	Reference
Custom feature-based approach	Seed	Elliptic Fourier descriptor, Haralick's texture descriptor, morpho-colorimetric feature	LDA	[38, 39]
	Grain	Shape, color, texture features	MLP	[36]
			ANFIS	[37]
	Leaf	SIFT, sparse coding	SVM	[40]
		Fourier descriptor, leaf shapes, vein structure		[56]
		Pretrained CNN		[35]
		Fast Features Invariant to Rotation and Scale of Texture (Ffirst)		[62]
		Texture features	LWSRC	[55]

	Bark	Fast Features Invariant to Rotation and Scale of Texture (Ffirst)	SVM	[62]
	Tree	Reflectance, minimum noise fraction transformation, narrowband vegetation indices, airborne imaging spectroscopy features	SVM, RF	[57]
	Grain			[59]
	Ear, spike, spikelet			[58]
CNN-based approach	Leaf	CNN		[52, 53, 61, 68]
	Root			[68]
	Various organs			[60-62]

Table 2

Click here to access/download;Table;Table2.docx

Table 2. Examples of approaches for classification of physiological states.

Approach	Object	Features/feature extractor	Classifier	Reference
Custom feature-based approach	Ear (growth stages)	SIFT + bag of keypoints	SVM	[47]
	Grain (quality assessment)	Weibull distribution model parameter features	SVM	[78]
	Leaf	Spectral vegetation indices	Spectral Angle Mapper	[79]
CNN-based approach	Leaf	CNN		[80, 81]

Table 3 Click here to access/download;Table;Table3.docx

Table 3. Software tools recently developed for plant image analysis, which use machine learning-based algorithms.

Name	Algorithms	Functionalities	Reference, URL
Leaf Necrosis Classifier	Multilayer perceptron and self-organizing maps	Detection of leaf areas showing necrotic symptoms	[112]
EasyPCC	Decision-tree-based segmentation model	Quantification of ground coverage ratio from image data acquired under field conditions	[113, 123]
Leaf-GP	Python-based machine learning libraries	Quantification of multiple growth phenotypes from large image series	[114, 124]
StomataCounter	Deep CNN	Counting stomate pores	[115]
Plantix	Deep learning	Diagnosing plant diseases, pest damages and nutrient deficiencies	[116]

Figure 1

Click here to access/download;Figure;Figure1.jpg

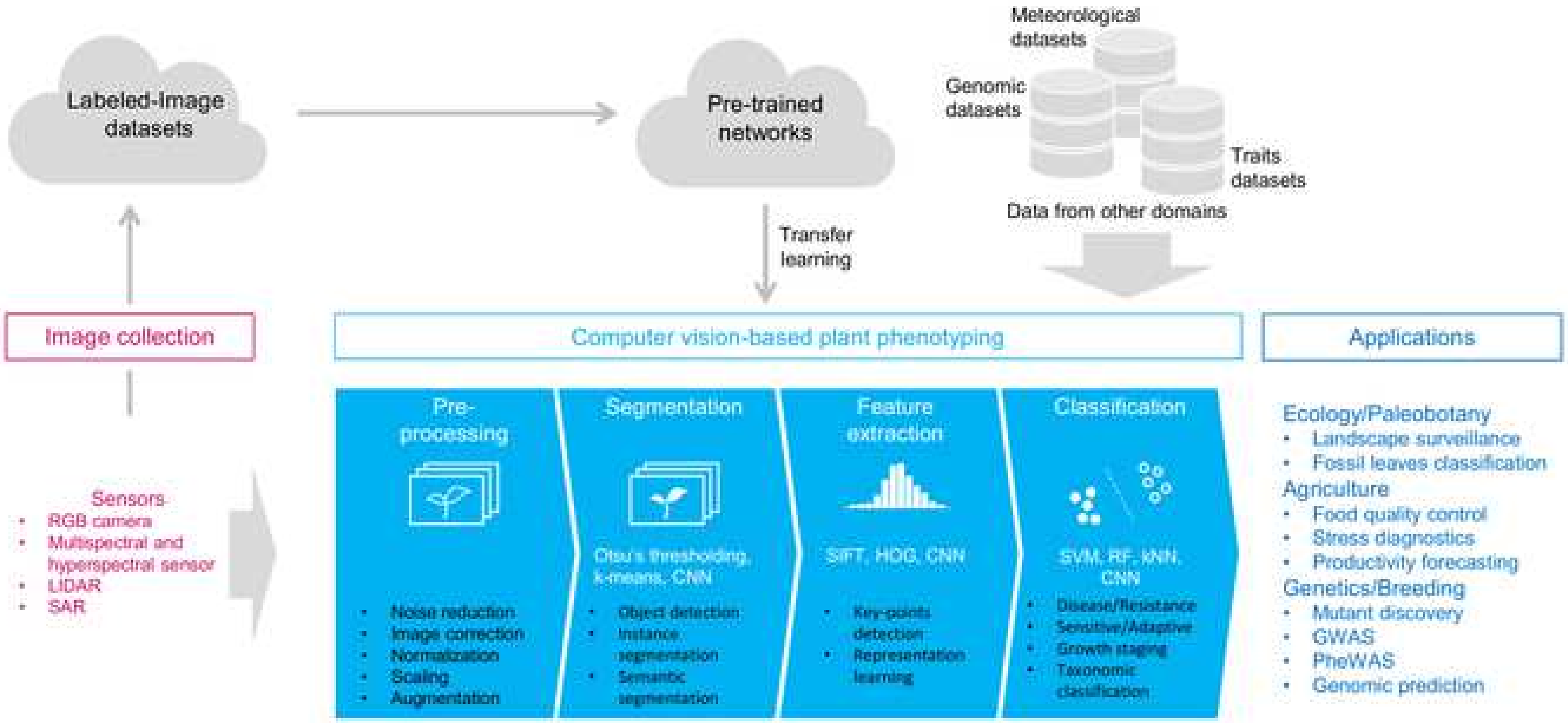

Figure 2

Click here to access/download;Figure;Figure2.jpg

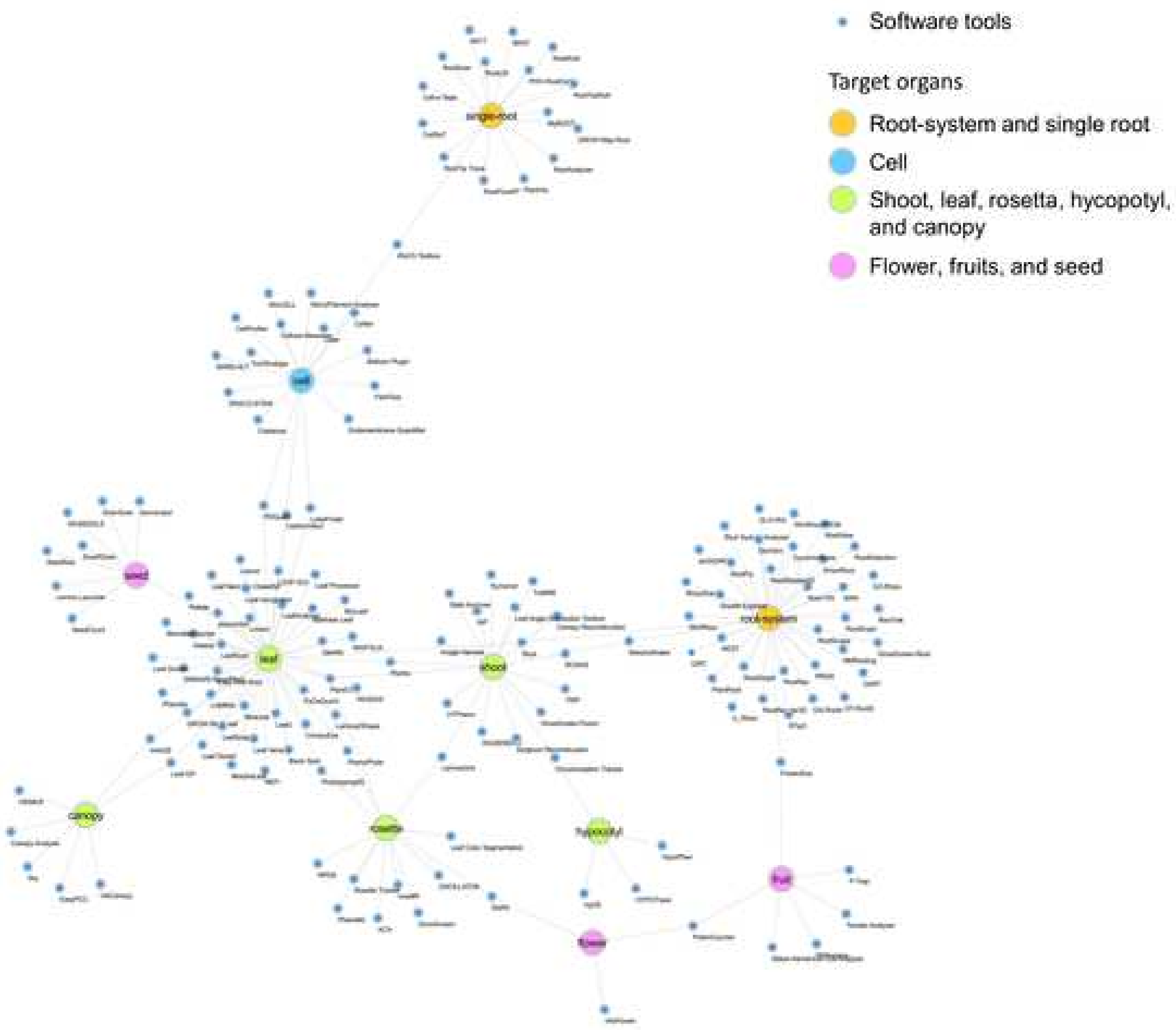

Response letter
Click here to access/download;Personal Cover;Response_letter_20180823.docx

September 6, 2018

Dear Dr. Nogoy:

We wish to express our appreciation to the editor and reviewer for their insightful comments on our manuscript. Their suggestions helped us to significantly improve the quality of the manuscript. To address the reviewers' concerns, we modified the structure of the main text, which clearly describes recent advances in computer vision-based phenotyping techniques and supports machine learning-based approaches aiming to discover genes for improvement of plant productivity. We have addressed all the comments from the reviewer, as detailed in our point-by-point list below. We hope the revised manuscript is now acceptable for publication in *GigaScience*.

Thank you for your consideration.

Sincerely,
Keiichi Mochida
Center for Sustainable Resource Science, RIKEN

Reviewer #1: The paper is timely and interesting. However, I have some major concerns with the structure and organisation of the paper, as well as some issue with missing material. Substantial research has been done by the authors examining the literature, but I feel a stronger paper could be produced which perhaps focuses on a particular topic inside the broad remit given, or focuses on presenting to a particular audience. I hope my comments below are useful in this process.
Response: We appreciate the comments from the reviewer and agree with the reviewer's suggestions. We have reorganized the manuscript accordingly and have made substantial revisions to improve the text.

-The title is very general. Although earlier reviews in this area are cited (e.g. https://www.sciencedirect.com/science/article/pii/S1360138515002630) it is not clear where this paper is positioned in relation to these existing reviews.
Response: We appreciate this comment from the reviewer. Accordingly, we have revised the title of the manuscripts to "**Computer vision-based phenotyping for improvement of plant productivity**", to more clearly describe the context of our review, which focuses on recent advances in computer vision-based phenotyping techniques aiming to discover

genes for improvement of plant productivity.

-Table 1 suggests a comprehensive summary of methods. Rather, these are a few sensible, but hand-picked examples of techniques. It is much better to present them as such (perhaps labeling them "Examples") rather than suggest these are the best/only approaches. It may even be better to remove the table and simply add the techniques to the literature review section.

Response: We thank the reviewer for this helpful comment and agree with the reviewer's suggestion. Accordingly, we have removed Table I and modified the section titled "Segmentation".

- There is a limited amount of narrative in the review. By which I mean, many of the papers seem to be presented one after another, without being linked together in some way. This process would help the reader form a more accurate picture of the landscape, rather than being presented with effectively a list of papers. I would recommend creating more of a narrative to structure the review. This comes back to my introductory point: what is the focus of this review, or who is it targeted towards? Knowing the answer to this will help form a strong structure to the narrative.

Response: We appreciate this comment from the reviewer. To improve the narrative in this review, we reorganized the structure of the manuscript and subheadings. Specifically, the revised structure follows the analytical process generally used in computer vision-based phenotyping described in Figure 1: image correction, preprocessing, segmentation, and classification. We also addressed recent examples of plant phenotyping that are particularly significant for plant gene discovery and highlighted the application of ML-based approaches. Next, we explained outcomes from recent attempts in plant phenotyping to identify genes related to plant traits.

-Subheadings: Some subheadings are used which is helpful, but I feel more could be used to break up the sections. This should tie in with a narrative structure mentioned in the above point.

Response: We thank the reviewer for this comment. To improve the structure of our manuscript, we revised the subheading of the main text as follows:

- High-throughput image collection for large-scale plant phenotyping: sensors and platforms
    - Sensors
    - Platforms

- Computer vision-based plant phenotyping
    - Preprocessing
    - Segmentation
    - Feature extraction
    - Classification
        - Taxonomic classification
        - Classification of plant physiological states
- Application of computer-vision assisted plant phenotyping for gene discovery
    - Autoscreening of mutants
    - Phenotyping for genetic mapping and prediction of agronomic traits
- Datasets and software tools for plant phenotyping
    - Datasets
    - Software tools
- Conclusions and perspectives

-Some of the review does not directly relate to machine learning, e.g. under the section starting 'L331 New tools and resources for plant phenomics' there are many items which do not seem related to machine learning, including some direct calculation of vegetative indices, e.g. NDVI. Do they belong in a machine learning review? If there is indeed a machine learning element to them, this needs to come across more clearly.

Response: We appreciate this helpful comment from the reviewer. When revising the manuscript, we have modified the text to include a section titled “High-throughput image collection for large-scale plant phenotyping” in order to discuss sensors and platforms for plant phenotyping and to introduce various indices from different sensors. We also summarize methods for data collection followed by image processing.

-Table 3 is inadequate, missing many important tools. Please see http://www.plant-image-analysis.org/ for a more comprehensive list.

Response: We thank the reviewer for this insightful suggestion. Accordingly, we revised Table 3 to describe more important software tools with ML-based algorithms; we believe this is more relevant to the context of the review. Moreover, to comprehensively evaluate the evolving ecosystem of software tools for plant image analysis, we added Figure 2 to explain the relationships between software tools in the plant-image-analysis database and target plant organs.

Some other minor comments:

L69 imagery->image, L72 aircrafts->aircraft, L76 thereby removing, L88 Do you need to also consider legislation of use for UAVs etc?, L89 *A* fixed-wing UAV..., L99 Beyond *the* latter

Response: We thank the reviewer for this comment. We have revised the manuscript and corrected these errors.

L106 I don't agree with your definition that ML is where algorithm design is improved automatically.

Response: We thank the reviewer for this comment. We corrected the definition of ML in the revised manuscript, as follows: "Machine learning (ML), an area of computer science, offers us data-driven prediction in various applications, including image analysis, which can aid typical steps of image analysis (i.e., preprocessing, segmentation, feature extraction, and classification)".

L129 What is meant by a commanded mobile phone?, L134 wording: keeping up with the growth of... ?, L137 361 field images from across China in the period 2010-2015, L138 'turned out to' - informal, consider rewording, L130 are these errors rates good or bad? Always best to present the context too, so the reader can decide, L142 'This paper' - which paper? The one you are writing, or a cited one - if so, which one?

Response: We appreciate this comment from the reviewer. We have revised the text accordingly to address these issues.

L169 - 171 this is an usual decision to make for a literature review? Perhaps it needs rewording, or reconsidering? Perhaps focusing the review would mean you don't need to write this.

Response: We thank the reviewer for this comment. We revised the text at the beginning of the taxonomic classification part as follows (L359–367 in the revised manuscript): "Computer vision-based taxonomic classification plays an essential role in plant phenotyping to automatically distinguish target species for phenotyping from other plants, which is particularly important for images from real fields. Wäldchen and Mäder have thoroughly summarized the literature on computer vision-based species identification published by 2016 [22]. In recent years, because techniques for computer vision-based species identification have shown dramatically improved accuracy and expanded applications for various plant groups through hand-crafted feature-based and CNN-based approaches, we highlight studies describing plant taxonomic classification by means of these two distinctive approaches (Table 2)."

L181 to classify

Response: We appreciate this comment from the reviewer. Accordingly, we have revised the text to clarify this point (L371 in the revised manuscript).

L254 detect*ing*

Response: We thank the reviewer for this comment. We have revised the text accordingly (L439 in the revised manuscript).

L257 is bag of visual words really often used? If so I think you need a number of references supporting this statement.

Response: We appreciate this comment from the reviewer. We have added references to support the use of the phrase “bag of visual words” (L339 in the revised manuscript).
