# Supplementary material for: Computer vision-based phenotyping for improvement of plant productivity: a machine learning perspective
Source: Gigascience. 2018 Dec 6;8(1):giy153. doi: 10.1093/gigascience/giy153 (PMC6312910; doi:10.1093/gigascience/giy153)
Supplement: Response_to_Reviewer_Comments_Original_Submission.pdf [file giy153_response_to_reviewer_comments_original_submission.pdf]

Author's Response To Reviewer Comments

Close

September 6, 2018

Dear Dr. Nogoy:

We wish to express our appreciation to the editor and reviewer for their insightful comments on our manuscript. Their suggestions helped us to significantly improve the quality of the manuscript. To address the reviewers’ concerns, we modified the structure of the main text, which clearly describes recent advances in computer vision-based phenotyping techniques and supports machine learning-based approaches aiming to discover genes for improvement of plant productivity. We have addressed all the comments from the reviewer, as detailed in our point-by-point list below. We hope the revised manuscript is now acceptable for publication in GigaScience.

Thank you for your consideration.

Sincerely,
Keiichi Mochida
Center for Sustainable Resource Science, RIKEN

Reviewer #1: The paper is timely and interesting. However, I have some major concerns with the structure and organisation of the paper, as well as some issue with missing material. Substantial research has been done by the authors examining the literature, but I feel a stronger paper could be produced which perhaps focuses on a particular topic inside the broad remit given, or focuses on presenting to a particular audience. I hope my comments below are useful in this process.
Response: We appreciate the comments from the reviewer and agree with the reviewer’s suggestions. We have reorganized the manuscript accordingly and have made substantial revisions to improve the text.

-The title is very general. Although earlier reviews in this area are cited (e.g. https://www.sciencedirect.com/science/article/pii/S1360138515002630) it is not clear where this paper is positioned in relation to these existing reviews.

Response: We appreciate this comment from the reviewer. Accordingly, we have revised the title of the manuscripts to “Computer vision-based phenotyping for improvement of plant productivity”, to more clearly describe the context of our review, which focuses on recent advances in computer vision-based phenotyping techniques aiming to discover genes for improvement of plant productivity.

-Table 1 suggests a comprehensive summary of methods. Rather, these are a few sensible, but hand-picked examples of techniques. It is much better to present them as such (perhaps labeling them "Examples") rather than suggest these are the best/only approaches. It may even be better to remove the table and simply add the techniques to the literature review section.

Response: We thank the reviewer for this helpful comment and agree with the reviewer's suggestion. Accordingly, we have removed Table I and modified the section titled "Segmentation".

- There is a limited amount of narrative in the review. By which I mean, many of the papers seem to be presented one after another, without being linked together in some way. This process would help the reader form a more accurate picture of the landscape, rather than being presented with effectively a list of papers. I would recommend creating more of a narrative to structure the review. This comes back to my introductory point: what is the focus of this review, or who is it targeted towards? Knowing the answer to this will help form a strong structure to the narrative.

Response: We appreciate this comment from the reviewer. To improve the narrative in this review, we reorganized the structure of the manuscript and subheadings. Specifically, the revised structure follows the analytical process generally used in computer vision-based phenotyping described in Figure 1: image correction, preprocessing, segmentation, and classification. We also addressed recent examples of plant phenotyping that are particularly significant for plant gene discovery and highlighted the application of ML-based approaches. Next, we explained outcomes from recent attempts in plant phenotyping to identify genes related to plant traits.

-Subheadings: Some subheadings are used which is helpful, but I feel more could be used to break up the sections. This should tie in with a narrative structure mentioned in the above point.

Response: We thank the reviewer for this comment. To improve the structure of our manuscript, we revised the subheading of the main text as follows:
- High-throughput image collection for large-scale plant phenotyping: sensors and platforms
- Sensors
- Platforms
- Computer vision-based plant phenotyping
- Preprocessing
- Segmentation
- Feature extraction
- Classification
- Taxonomic classification
- Classification of plant physiological states
- Application of computer-vision assisted plant phenotyping for gene discovery
- Autoscreening of mutants
- Phenotyping for genetic mapping and prediction of agronomic traits
- Datasets and software tools for plant phenotyping
- Datasets
- Software tools
- Conclusions and perspectives

-Some of the review does not directly relate to machine learning, e.g. under the section

starting 'L331 New tools and resources for plant phenomics' there are many items which do not seem related to machine learning, including some direct calculation of vegetative indices, e.g. NDVI. Do they belong in a machine learning review? If there is indeed a machine learning element to them, this needs to come across more clearly.

Response: We appreciate this helpful comment from the reviewer. When revising the manuscript, we have modified the text to include a section titled "High-throughput image collection for large-scale plant phenotyping" in order to discuss sensors and platforms for plant phenotyping and to introduce various indices from different sensors. We also summarize methods for data collection followed by image processing.

-Table 3 is inadequate, missing many important tools. Please see http://www.plant-image-analysis.org/ for a more comprehensive list.

Response: We thank the reviewer for this insightful suggestion. Accordingly, we revised Table 3 to describe more important software tools with ML-based algorithms; we believe this is more relevant to the context of the review. Moreover, to comprehensively evaluate the evolving ecosystem of software tools for plant image analysis, we added Figure 2 to explain the relationships between software tools in the plant-image-analysis database and target plant organs.

Some other minor comments:
L69 imagery->image, L72 aircrafts->aircraft, L76 thereby removing, L88 Do you need to also consider legislation of use for UAVs etc?, L89 *A* fixed-wing UAV..., L99 Beyond *the* latter

Response: We thank the reviewer for this comment. We have revised the manuscript and corrected these errors.

L106 I don't agree with your definition that ML is where algorithm design is improved automatically.

Response: We thank the reviewer for this comment. We corrected the definition of ML in the revised manuscript, as follows: "Machine learning (ML), an area of computer science, offers us data-driven prediction in various applications, including image analysis, which can aid typical steps of image analysis (i.e., preprocessing, segmentation, feature extraction, and classification)".

L129 What is meant by a commanded mobile phone?, L134 wording: keeping up with the growth of... ?, L137 361 field images from across China in the period 2010-2015, L138 'turned out to' - informal, consider rewording, L130 are these errors rates good or bad? Always best to present the context too, so the reader can decide, L142 'This paper' - which paper? The one you are writing, or a cited one - if so, which one?

Response: We appreciate this comment from the reviewer. We have revised the text accordingly to address these issues.

L169 - 171 this is an usual decision to make for a literature review? Perhaps it needs rewording, or reconsidering? Perhaps focusing the review would mean you don't need to write this.

Response: We thank the reviewer for this comment. We revised the text at the beginning of the taxonomic classification part as follows (L359–367 in the revised manuscript): "Computer vision-based taxonomic classification plays an essential role in plant phenotyping to automatically distinguish target species for phenotyping from other plants, which is particularly important for images from real fields. Wäldchen and Mäder have thoroughly summarized the literature on computer vision-based species identification published by 2016 [22]. In recent years, because techniques for computer vision-based species identification have shown dramatically improved accuracy and expanded applications for various plant groups through hand-crafted feature-based and CNN-based approaches, we highlight studies describing plant taxonomic classification by means of these two distinctive approaches (Table 2)."

L181 to classify

Response: We appreciate this comment from the reviewer. Accordingly, we have revised the text to clarify this point (L371 in the revised manuscript).

L254 detect*ing*

Response: We thank the reviewer for this comment. We have revised the text accordingly (L439 in the revised manuscript).

L257 is bag of visual words really often used? If so I think you need a number of references supporting this statement.

Response: We appreciate this comment from the reviewer. We have added references to support the use of the phrase "bag of visual words" (L339 in the revised manuscript).

Close
