# Supplementary material for: Computer vision-based phenotyping for improvement of plant productivity: a machine learning perspective
Source: Gigascience. 2018 Dec 6;8(1):giy153. doi: 10.1093/gigascience/giy153 (PMC6312910; doi:10.1093/gigascience/giy153)
Supplement: Response_to_Reviewer_Comments_Revision_1.pdf [file giy153_response_to_reviewer_comments_revision_1.pdf]

Author's Response To Reviewer Comments

Close

The reviewer has a few minor final suggestions to improve the paper. We also agree with the suggested title change - in your revision, please edit the title to include some mention of machine learning, and suggest (like the reviewer) to change the title to read as "Computer vision-based phenotyping for improvement of plant productivity: a machine learning perspective".
Response: We appreciate this suggestion from the editor and agree with the recommended title. We have revised the title of our manuscript accordingly.

Reviewer #1: Hi
Thanks for considering my comments in your restructuring of the article
I have a few things to clear up below:
Response: We appreciate the additional suggestions from the reviewer.

1. The title: I suggested the last title was too broad, it is now even more broad! I think it does need a machine learning aspect in there somewhere. What about:

Computer vision-based phenotyping for improvement of plant productivity: a machine learning perspective
(This is a suggestion - happy to go with the Editor's opinion)
Response: We thank the reviewer for this suggestion. As recommended by the Editor and reviewer, we agree with the suggested title and have revised the title accordingly.

2. You state "ML-based algorithms often provide deeper insights into discriminative features". I am not sure this is true, does it really provide deeper *insights*? I think this is probably a wording issue, which needs resolving.
Response: We thank the reviewer for this comment. According to the reviewer's suggestion, we have revised the sentence as follows: "ML-based algorithms often provide discriminative features associated with outputs extracted through their training process".

3. Figure 1. Please state in the legend this is one typical example scenario (there are many potential pipelines possible - this is just one)
Response: We appreciate this comment from the reviewer. We have revised the legend as follows: "Schematic representation of a typical example scenario in computer vision-based plant phenotyping".
In the restructuring you focus on the "typical steps" of image analysis a few times (preprocessing, segmentation, feature extraction, and classification). It is worth mentioning in the article that with deep networks, these typical steps apply less (direct image classification or counting is possible, in an end-to-end framework).
Response: We appreciate this insightful suggestion. In the revised manuscript, we added a paragraph (beginning with "Deep neural network-based image analysis with end-to-end learning") in the last part of the "Computer vision-based plant phenotyping" section. In this paragraph, we briefly introduced recently proposed CNN-based frameworks enabling end-to-end training together with examples in plant phenotyping applications.

Close
