# Supplementary material for: Computer vision-based phenotyping for improvement of plant productivity: a machine learning perspective
Source: Gigascience. 2018 Dec 6;8(1):giy153. doi: 10.1093/gigascience/giy153 (PMC6312910; doi:10.1093/gigascience/giy153)
Supplement: Reviewer_1_Report_1_(Original_Submission) -- Andrew French — 7/3/2018 Reviewed [file giy153_reviewer_1_report_1_(original_submission).pdf]

Reviewer Report

Title: Computer vision-based phenotyping for improvement of plant productivity: a machine learning perspective

Version: Original Submission **Date:** 7/3/2018

Reviewer name: Andrew French

Reviewer Comments to Author:

The paper is timely and interesting. However, I have some major concerns with the structure and organisation of the paper, as well as some issue with missing material. Substantial research has been done by the authors examining the literature, but I feel a stronger paper could be produced which perhaps focuses on a particular topic inside the broad remit given, or focuses on presenting to a particular audience. I hope my comments below are useful in this process.

-The title is very general. Although earlier reviews in this area are cited (e.g. https://www.sciencedirect.com/science/article/pii/S1360138515002630) it is not clear where this paper is positioned in relation to these existing reviews.

-Table 1 suggests a comprehensive summary of methods. Rather, these are a few sensible, but hand-picked examples of techniques. It is much better to present them as such (perhaps labeling them "Examples") rather than suggest these are the best/only approaches. It may even be better to remove the table and simply add the techniques to the literature review section.

- There is a limited amount of narrative in the review. By which I mean, many of the papers seem to be presented one after another, without being linked together in some way. This process would help the reader form a more accurate picture of the landscape, rather than being presented with effectively a list of papers. I would recommend creating more of a narrative to structure the review. This comes back to my introductory point: what is the focus of this review, or who is it targeted towards? Knowing the answer to this will help form a strong structure to the narrative.

-Subheadings: Some subheadings are used which is helpful, but I feel more could be used to break up the sections. This should tie in with a narrative structure mentioned in the above point.

-Some of the review does not directly relate to machine learning, e.g. under the section starting 'L331 New tools and resources for plant phenomics' there are many items which do not seem related to machine learning, including some direct calculation of vegetative indices, e.g. NDVI. Do they belong in a machine learning review? If there is indeed a machine learning element to them, this needs to come across more clearly.

-Table 3 is inadequate, missing many important tools. Please see http://www.plant-image-analysis.org/ for a more comprehensive list.

Some other minor comments:

L69 imagery->image
L72 aircrafts->aircraft
L76 thereby removing
L88 Do you need to also consider legislation of use for UAVs etc?
L89 *A* fixed-wing UAV...

L99 Beyond *the* latter
L106 I don't agree with your definition that ML is where algorithm design is improved automatically.
L129 What is meant by a commanded mobile phone?
L134 wording: keeping up with the growth of... ?
L137 361 field images from across China in the period 2010-2015
L138 'turned out to' - informal, consider rewording
L130 are these errors rates good or bad? Always best to present the context too, so the reader can decide.
L142 'This paper' - which paper? The one you are writing, or a cited one - if so, which one?
L169 - 171 this is an usual decision to make for a literature review? Perhaps it needs rewording, or reconsidering? Perhaps focusing the review would mean you don't need to write this.
L181 to classify
L254 detect*ing*
L257 is bag of visual words really often used? If so I think you need a number of references supporting this statement.

Level of Interest

Please indicate how interesting you found the manuscript: Choose an item.

Quality of Written English

Please indicate the quality of language in the manuscript: Choose an item.

Declaration of Competing Interests

Please complete a declaration of competing interests, considering the following questions:

- Have you in the past five years received reimbursements, fees, funding, or salary from an organisation that may in any way gain or lose financially from the publication of this manuscript, either now or in the future?
- Do you hold any stocks or shares in an organisation that may in any way gain or lose financially from the publication of this manuscript, either now or in the future?
- Do you hold or are you currently applying for any patents relating to the content of the manuscript?
- Have you received reimbursements, fees, funding, or salary from an organization that holds or has applied for patents relating to the content of the manuscript?
- Do you have any other financial competing interests?
- Do you have any non-financial competing interests in relation to this paper?

If you can answer no to all of the above, write 'I declare that I have no competing interests' below. If your reply is yes to any, please give details below.

I declare I have no competing interestes.

I agree to the open peer review policy of the journal. I understand that my name will be included on my report to the authors and, if the manuscript is accepted for publication, my named report including any

attachments I upload will be posted on the website along with the authors' responses. I agree for my report to be made available under an Open Access Creative Commons CC-BY license (http://creativecommons.org/licenses/by/4.0/). I understand that any comments which I do not wish to be included in my named report can be included as confidential comments to the editors, which will not be published.

I agree to the open peer review policy of the journal

To further support our reviewers, we have joined with Publons, where you can gain additional credit to further highlight your hard work (see: https://publons.com/journal/530/gigascience). On publication of this paper, your review will be automatically added to Publons, you can then choose whether or not to claim your Publons credit. I understand this statement. Yes
