# Supplementary material for: Computer vision-based phenotyping for improvement of plant productivity: a machine learning perspective
Source: Gigascience. 2018 Dec 6;8(1):giy153. doi: 10.1093/gigascience/giy153 (PMC6312910; doi:10.1093/gigascience/giy153)
Supplement: Reviewer_1_Report_1_Revision_1 -- Andrew French — 9/25/2018 Reviewed [file giy153_reviewer_1_report_1_revision_1.pdf]

Reviewer Report

Title: Computer vision-based phenotyping for improvement of plant productivity: a machine learning perspective

Version: Original Submission **Date:** 9/25/2018

Reviewer name: Andrew French

Reviewer Comments to Author:

Hi

Thanks for considering my comments in your restructuring of the article

I have a few things to clear up below:

1. The title: I suggested the last title was too broad, it is now even more broad! I think it does need a machine learning aspect in there somewhere. What about:

Computer vision-based phenotyping for improvement of plant productivity: a machine learning perspective
(This is a suggestion - happy to go with the Editor's opinion)

2. You state "ML-based algorithms often provide deeper insights into discriminative features". I am not sure this is true, does it really provide deeper *insights*? I think this is probably a wording issue, which needs resolving.

3. Figure 1. Please state in the legend this is one typical example scenario (there are many potential pipelines possible - this is just one)

In the restructuring you focus on the "typical steps" of image analysis a few times (preprocessing, segmentation, feature extraction, and classification). It is worth mentioning in the article that with deep networks, these typical steps apply less (direct image classification or counting is possible, in an end-to-end framework).

Thank you.

Level of Interest

Please indicate how interesting you found the manuscript: Choose an item.

Quality of Written English

Please indicate the quality of language in the manuscript: Choose an item.

Declaration of Competing Interests

Please complete a declaration of competing interests, considering the following questions:

- Have you in the past five years received reimbursements, fees, funding, or salary from an organisation that may in any way gain or lose financially from the publication of this manuscript, either now or in the future?
- Do you hold any stocks or shares in an organisation that may in any way gain or lose financially from the publication of this manuscript, either now or in the future?
- Do you hold or are you currently applying for any patents relating to the content of the manuscript?
- Have you received reimbursements, fees, funding, or salary from an organization that holds or has applied for patents relating to the content of the manuscript?
- Do you have any other financial competing interests?
- Do you have any non-financial competing interests in relation to this paper?

If you can answer no to all of the above, write 'I declare that I have no competing interests' below. If your reply is yes to any, please give details below.

I declare I have no competing interestes.

I agree to the open peer review policy of the journal. I understand that my name will be included on my report to the authors and, if the manuscript is accepted for publication, my named report including any attachments I upload will be posted on the website along with the authors' responses. I agree for my report to be made available under an Open Access Creative Commons CC-BY license (http://creativecommons.org/licenses/by/4.0/). I understand that any comments which I do not wish to be included in my named report can be included as confidential comments to the editors, which will not be published.

I agree to the open peer review policy of the journal

To further support our reviewers, we have joined with Publons, where you can gain additional credit to further highlight your hard work (see: https://publons.com/journal/530/gigascience). On publication of this paper, your review will be automatically added to Publons, you can then choose whether or not to claim your Publons credit. I understand this statement. Yes
